# Police programmes that seek to increase community connectedness for reducing violent extremism behaviour, attitudes and beliefs

**DOI:** 10.1002/cl2.1111

**Published:** 2020-09-08

**Authors:** Lorraine Mazerolle, Elizabeth Eggins, Adrian Cherney, Lorelei Hine, Angela Higginson, Emma Belton

**Affiliations:** ^1^ Faculty of Humanities and Social Sciences, School of Social Science, St Lucia Campus University of Queensland St Lucia Queensland Australia; ^2^ Faculty of Law, School of Justice Research, Gardens Point Campus Queensland University of Technology Brisbane Queensland Australia

## Abstract

**Background:**

Police can play a role in tackling violent extremism through disrupting terrorist plots and by working with communities to identify individuals at risk of radicalisation. Police programmes to tackle violent extremism can involve a range of approaches and partnerships. One approach includes efforts to improve community connectedness by working to address social isolation, belonging, economic opportunities and norms and values that may lead people to endorse or support violent extremist causes and groups. The assumption is that the risk of an individual being radicalised in the community can be reduced when police work in pothe international legal ordersitive ways with community members and groups to mobilise and support activities that help generate a sense of belonging and trust. Police programmes that build a sense of belonging and trust may help ensure individuals are not influenced by activities that violent extremists use to attract support for their cause.

**Objectives:**

The review aimed to systematically examine whether or not police programmes that seek to promote community connectedness are effective in reducing violent extremist behaviours, attitudes and beliefs. The review also sought to identify whether effectiveness varied by the intervention type and location.

**Search Methods:**

Using terrorism‐related terms, we searched the Global Policing Database to identify eligible published and unpublished evaluations between January 2002 and December 2018. We supplemented this with comprehensive searches of relevant terrorism and counter‐terrorism websites and research repositories, reference harvesting of eligible and topic‐relevant studies, forward citation searches of eligible studies, hand‐searches of leading journals and consultations with experts.

**Selection Criteria:**

Eligible studies needed to include an initiative that involved the police, either through police initiation, development, leadership or where the police were receivers of the programme (such as a training programme) or where the police delivered or implemented the intervention. The initiative also needed to be some kind of a strategy, technique, approach, activity, campaign, training, programme, directive or funding/organisational change that involved police in some way to promote community connectedness. Community connectedness was defined as being community consultation, partnership or collaboration with citizens and/or organisational entities. Eligible outcomes included violent extremism, along with radicalisation and disengagement which are considered to be attitudinal and belief‐based components of violent extremism. These outcomes could be measured via self‐report instruments, interviews, observations and/or official data. To be included, studies could utilise individuals, micro‐ or macroplaces as the participants. Finally, studies needed to provide a quantitative impact evaluation that utilised a randomised or quasi‐experimental design with a comparison group that either did not receive the intervention, or that received “business‐as‐usual” policing, no intervention or an alternative intervention.

**Data Collection and Analysis:**

The systematic search identified 2,273 records (after duplicate removal). After systematic screening across two stages (title/abstract and full‐text), just one study (reported in two documents) met the review eligibility criteria. Standardised mean differences (SMD) were used to estimate intervention effects for this single study and risk of bias was assessed using the Cochrane Risk of Bias in Non‐Randomised Studies‐Interventions tool (ROBINS‐I).

**Results:**

The single eligible study (*n* = 191) was a quasi‐experimental evaluation of the Muslim‐led intervention—World Organisation for Resource Development Education (WORDE)—conducted in the United States in 2015. The intervention comprised three components: community education, enhancing agency networks and multicultural volunteerism activities. Self‐report data were collected from youth and adults who were civically engaged, sensitised to issues of violent extremism and who had existing cooperative relationships with law enforcement and social services. The comparison group comprised matched participants who had not engaged with the WORDE programme. The outcomes most closely aligned with conceptual definitions of deradicalization, specifically levels of acceptance and/or engagement with cultural and religious differences or pluralistic views and modification of group or personal identity. Based on single survey items, the SMD ranged from small to medium in favour of the treatment group aside from one item which favoured the comparison group (“*I make friends with people from other races*”, SMD = −0.51, 95% CI: −0.82, −0.19). However, of the nine SMDs calculated, six had confidence intervals including zero. These effects should be interpreted with caution due to the study's overall serious risk of bias. It is important to note that it is not explicitly clear whether the evaluation participants in the treatment group were all directly exposed to the two intervention components that involved police. Hence, these evaluation outcomes may not be direct measures of how effect police were at countering violent extremism by promoting community connectiveness.

**Conclusions:**

The aim of this systematic review was to examine whether or not police programmes that seek to promote community connectedness are effective in reducing violent extremist behaviours, attitudes and beliefs. There is insufficient evidence available to ascertain whether such interventions achieve these outcomes. This finding is the result of the fact that interventions that have been evaluated tend to be characterised by evaluation designs that do not adopt experimental or quasi‐experimental approaches or use outcomes that are outside of scope for this review. While the volume of studies identified provide support for the assertion that police can play a role in tackling violent extremism by participating in, and implementing, programmes that promote community connectedness, it is unclear at this time if such approaches work in reducing violent extremism. Whilst we conclude that investment needs to be made in more robust methods of evaluation to test for programme effectiveness, we acknowledge that conducting evaluation and research in the area of counter‐terrorism/violent extremism is challenging.

## 1 PLAIN LANGUAGE SUMMARY

### 1.1 There is limited evidence of how police programmes to generate community connectedness affect violent extremist behaviours, attitudes and beliefs

Police programmes to generate community connectedness are assumed to help reduce risk factors that lead individuals to radicalise to violent extremism. There is no robust body of evaluation evidence to verify this claim. This lack of evidence is because programme funders have not sufficiently invested in impact evaluations of policing programmes that aim to counter violent extremism by promoting community connectedness.

### 1.2 What is this review about?

Community connectedness and efforts to engage communities may help to mitigate the risk of individuals radicalising to violent extremism. Police, under some circumstances, can play a key role in programmes aimed at tackling violent extremism. This includes working with communities and other agencies to tackle social isolation, economic opportunity and norms and beliefs that lead individuals and groups to radicalise and support extremist causes.

This review looked at whether or not strategies involving police in the initiation, development or implementation of programmes aimed at community connectedness had an impact on reducing violent extremist beliefs and behaviours.

**What is the aim of this review?**

This Campbell systematic review examines whether police programmes aiming to improve community connectedness have an effect on violent extremist behaviour, attitudes and beliefs. It summarises evidence from one study that met the inclusion criteria and references others that describe types of interventions, but that have not been rigorously evaluated.

### 1.3 What studies are included?

The review includes studies that evaluated programmes aimed at countering violent extremism by promoting community connectedness. The interventions included in the review needed to have a police focus, where the intervention involved police as the receivers of an intervention and/or partners in the development, initiation and implementation of a programme. The intervention could be focused on individuals, places (e.g., schools), neighbourhoods or larger geographical locations.

Although the systematic search captured 2,273 potential studies, only one study met the review inclusion criteria. This study was conducted in 2015 in the USA.

### 1.4 What are the findings of this review?

The one included study was a Muslim community‐led initiative involving police that aimed to counter violent extremism through a community‐based education and awareness programme. The programme aimed to improve referral networks for agencies/third parties to help assist individuals identified as at‐risk of radicalisation.

Evidence from this study showed mixed small‐to‐medium effects on self‐reported deradicalization measures in favour of the treatment group. Eight out of nine calculated effect sizes favoured the intervention, though six of these were statistically insignificant. One survey item favoured the comparison group: “I make friends with people from other races”. However, these results need to be interpreted with caution due to the study limitations.

Given the low number of studies identified, the authors have also provided a summary of a small sample of studies reporting on interventions that aligned with the review topic but did not meet the inclusion criteria due to weak evaluation designs. These studies illustrate a range of approaches being used by the police, such as recreation and sports activities, and community education and engagement around countering violent extremism and related topics.

### 1.5 What do the findings of this review mean?

There is currently insufficient evidence to establish whether police programmes aimed at countering violent extremism by promoting community connectedness are effective. Although the evidence identified by this review shows that such programmes are being implemented, they have not yet been rigorously evaluated. Future research should aim to rigorously evaluate such initiatives.

### 1.6 How up‐to‐date is this review?

The review authors searched for studies up to December 2018.

## 2 BACKGROUND

### 2.1 The problem, condition or issue

Community engagement and connectedness are identified as potential mitigating factors for those at risk of engaging in violent extremism (Cherney & Hartley, 2017). A focus on inclusion, social connectedness and positive cultural norms is essential for prevention efforts designed to build inclusive communities and weaken the influence of extremist messages and recruiters (Grossman, Peucker, Smith, & Dellal, 2016; Van Den Bos, 2018; Schanzer, Kurzman, Toliver, & Miller, 2016). Growing research suggests that cohesive communities are resilient against violent extremist influences; for example, it is argued that a greater sense of belonging and acceptance can reduce extremist behaviour, attitudes and beliefs (Cherney, Bell, Leslie, Cherney, & Mazerolle, 2018; Grossman et al., 2016; Van Den Bos, 2018).

As frontline practitioners, police are well placed to promote social inclusion and social connectedness, and thereby preventing violent extremism. In the general policing literature, when police engagement with the public is undertaken in an inclusive and fair manner, police can be instrumental in fostering a deep understanding of the local communities they police, creating opportunities for improving community relations (Gill, Weisburd, Telep, Vitter, & Bennett, 2014). Strong police‐community relations are likely an important foundation for police being in a position to identify individuals who might be at risk of radicalisation and violent extremism and then work with community leaders to counter the influence of a variety of different types of violent extremist groups including those from the far right, the far left, environmental extremism, political and religious extremism groups. Police can, therefore, be key agents in promoting community connectedness, working with community members to build trust, minimise social distancing—particularly amongst culturally diverse communities—and strengthen a sense of belonging by showing that they have the interests of the community at heart (Cherney & Hartley, 2017; Murray, Mueller‐Johnson, & Sherman, 2015).

Increasingly, police are working with a range of different agencies, together actively engaging the community to reduce social isolation, improve economic opportunity and aim to create social and cultural norms that prevent violent extremism (Schanzer et al., 2016). Yet it is unclear whether or not the range of police initiatives that foster community connectedness are able to reduce violent extremism. Thus, it is essential to understand the effectiveness of policing programmes aimed at promoting community connectedness and their impact on reducing violent extremism.

### 2.2 The intervention

This review aimed to include any policing intervention that aims to promote community connectedness, which was defined by the presence of two components. First, the intervention must have had a *policing* focus, defined as some kind of a strategy, technique, approach, activity, campaign, training, programme, directive or funding/organisational change that involves police in some way (other agencies or organisations can be involved; Higginson, Eggins, Mazerolle, & Stanko, 2015). Police involvement was broadly defined as follows.

- Police initiation, development or leadership of the intervention.
- Police are receivers of the intervention (such as being the receivers of training programmes or on the receiving end of initiatives aimed to engage police in the activities of another agency) or the intervention is related, focused or targeted to police practices.
- Delivery or implementation of the intervention by police.

Second, the policing intervention must have *aimed to promote community connectedness*. For the purposes of this review, we defined the promotion of community connectedness to mean an intention to increase prosocial linkages or prosocial ties between either community members themselves, community members and police, or community members and people in businesses, houses of worship, schools or any other community‐based organisation. Other terminology that may be used to represent connectedness in the literature includes (Thomas, 2019) the following.

- Promotion of common values, norms and/or reciprocity.
- Promotion of social networks, collective efficacy, social cohesion or social capital.
- Promotion of shared problem solving or citizen engagement.

We anticipated that policing interventions aiming to promote community connectedness would likely overlap with initiatives labelled community policing (see Gill et al., 2014) or other policing approaches that often aim to enhance community connectedness (e.g., neighbourhood policing or legitimacy approaches). However, we specifically defined a policing intervention that aims to promote community connectedness as being characterised by community consultation, partnership or collaboration with citizens and/or organisational entities. Specific strategies may have included the following:

- community meetings or forums;
- developing partnerships with specific organisations (Fox, 2012);
- police liaison programmes involving community members (Cherney et al., 2018);
- police work with community leaders to enhance personal skills (e.g., self‐identity, self‐awareness and resilience), employment skills (e.g., teamwork and self‐awareness), or leadership skills (Thomas, 2019);
- routine police work (such as beat policing, foot patrols, community intelligence initiatives) that explicitly seek to promote community connectedness;
- specific initiatives—such as neighbourhood policing teams—that seek to promote community connectedness; or
- police legitimacy enhancing programmes that seek to promote a sense of belonging and inclusion within local communities.

### 2.3 How the intervention might work

Police programmes that seek to reduce violent extremist behaviours and beliefs through improving community connectedness aim to generate an impact by promoting an increased sense of prosocial belonging and inclusion amongst at‐risk groups. This causal pathway is underpinned by key perspectives in the literature that argue how people are treated by institutional authorities, such as police, has an impact on their sense of identity and belonging by making them feel accepted by broader society (Mazerolle et al., 2014).

Social identity theory and the group value model (see Tyler & Lind, 1992) demonstrates that the ways that police engage with citizens will differentially affect the way that people perceive the police and thereby their willingness to comply with directives (see also Bradford, Murphy, & Jackson, 2014; Huo, Smith, Tyler, & Lind, 1996).

Some research shows that procedural fairness is more important for those on the margins (De Cremer & Sedikides, 2005; Murphy, 2013). Other research finds that the procedural justice, social identity and legitimacy pathway is found amongst both those with high and low group identifications (Bradford *et al*. 2014). Outcomes of police initiatives that build a sense of belonging and inclusion are, therefore, assumed to act as protective factors against radicalisation by ensuring individuals are not influenced by the messaging and grievance narratives that violent extremists use to attract support. We acknowledge, however, Nagin and Telep's (2017) review of the evidence challenging the causal relationship between perceptions of procedurally just treatment of citizens by agents of the criminal justice system and perceptions of police legitimacy. These authors conclude that perceptions of procedurally just treatment are associated with perceptions of police legitimacy and legal compliance (see also Donner, Maskaly, Fridell, & Jennings, 2015; Jackson, Hough, Bradford, & Kuha, 2015; Tyler 2004; Tyler, Goff, & MacCoun, 2015), yet that these associations are not necessarily reflective of causal connections.

As it relates specifically to tackling violent extremism, the quality of police engagement with different types of communities—such as the Muslim community—has been shown to influence the degree to which people are willing to partner with police to tackle terrorism (Cherney & Murphy, 2017). Police are seen as legitimate authorities and representatives of the state and when they work with community groups. This helps builds police legitimacy and has a spill over effect on people's sense of belonging and inclusion.

### 2.4 Why it is important to do the review

Community engagement approaches have become a key component of police counter‐terrorism efforts (Cherney & Hartley, 2017). These strategies have emphasised community engagement and outreach to identify potential violent extremism threats. This has involved police programmes that aim to promote collaborative problem solving between police and community members to tackle radicalisation, such as through identifying youth at risk of radicalising to violent extremism (Cherney, 2018). For example, following 9/11, police units in Australia, the United Kingdom, the United States, and Canada were established to undertake outreach with particular community groups (e.g., Muslim communities), with the aim of tackling violence extremism by enhancing relations and connectedness between police and these communities, and also between community members (Cherney, 2018; Ramirez, 2012). However, to date, there has been no systematic synthesis of the evaluation evidence for these policing approaches and their impact on violent extremism.^1^ Therefore, the current review is necessary to ascertain whether policing interventions that seek to promote community connectedness are effective for reducing violent extremism behaviour, attitudes and beliefs. In addition, the results from this review can be used to inform future decision making relating to both the design and evaluation of police programmes by identifying gaps in the evidence‐base and level of investment needed in evaluation of primary studies.

## 3 OBJECTIVES

The primary objective of this review was to answer the question: how effective are police programmes that seek to increase community connectedness for reducing violent extremism attitudes, beliefs and behaviours? A secondary objective was to also examine whether the effectiveness of these interventions vary by the following factors: geographical location, target population and type of policing strategy used to promote connectedness.

## 4 METHODS

### 4.1 Criteria for considering studies for this review

#### 4.1.1 Types of studies

This review includes quantitative impact evaluations that utilise a randomised experimental (e.g., RCTs) or a quasi‐experimental design with a comparison group that does not receive the intervention. We will include studies where the comparison group receives “business‐as‐usual” policing, no intervention or an alternative intervention (treatment–treatment designs).

Although not as robust as RCTs, “strong” quasi‐experiments can be used to provide causal inference when there are elements of the design that aim to minimise threats to internal validity (see Farrington, 2003; Shadish, Cook, & Campbell, 2002). Minimising threats to internal validity can include: controlling case assignment to treatment and control groups (regression discontinuity), matching characteristics of the treatment and control groups (matched control), statistically accounting for differences between the treatment and control groups (designs using multiple regression analysis) or providing a difference‐in‐difference analysis (parallel cohorts with pre‐test and post‐test measures). Therefore, we included the following “strong” quasi‐experimental designs in this review:

- cross‐over designs;
- regression discontinuity designs;
- designs using multivariate controls (e.g., multiple regression);
- matched control group designs with or without preintervention baseline measures (propensity or statistically matched);
- unmatched control group designs without preintervention measures where the control group has face validity;
- unmatched control group designs with pre‐post intervention measures that allow for difference‐in‐difference analysis;
- short interrupted time‐series designs with control group^2^ (less than 25 pre‐ and 25 postintervention observations; Glass, 1997);
- long interrupted time‐series designs with or without a control group (≥25 pre‐ and postintervention observations; Glass, 1997).

Weaker quasi‐experimental designs can be used to demonstrate the magnitude of the relationship between an intervention and an outcome. However, we excluded the following weaker quasi‐experimental designs due to their limitations in establishing causality.

- Raw unadjusted correlational designs where the variation in the level of the intervention is compared to the variation in the level of the outcome.
- Single group designs with pre‐ and postintervention measures.

#### 4.1.2 Types of participants

We included studies that considered the impact of community connectedness policing interventions on the following population subjects:

1. individuals of any age, gender or ethnicity;
2. microplaces (e.g., street corners, buildings, police beats, street segments);
3. macroplaces (e.g., neighbourhoods or larger geographies).

This review aimed to include programmes focused on individuals and groups identified as at‐risk violent extremism due to beliefs and or associations, as well as those who have acted on those beliefs. This was to ensure we captured police programmes tackling different levels of violent extremism. However, to be included in the review, participants did not need to be classified at‐risk or have displayed extremist behaviours. We placed no limits on the geographical region reported in the study. Specifically, we included studies conducted in high‐, low‐ and middle‐income countries in the review.

### 4.2 Types of interventions

This review aimed to include any policing intervention that aims to promote community connectedness. Specifically, to be included in the review, a study must have met the following two intervention criteria.

1. Report on a *policing intervention*, defined as some kind of a strategy, technique, approach, activity, campaign, training, programme, directive or funding/organisational change that involves police in some way (other agencies or organisations can be involved; Higginson et al., 2015). Police involvement is broadly defined as:

- police initiation, development or leadership;
- police are receivers of the intervention or the intervention is related, focused or targeted to police practices; or
- delivery or implementation of the intervention by police.

2. Report on a policing intervention that *aims to promote community connectedness*. For the purposes of this review, we define the promotion of community connectedness to mean an intention to increase linkages or ties between either the community members themselves, or community members and police. Other terminology that may be used to represent connectedness in the literature includes (Thomas, 2019):

- promotion of common values, norms and/or reciprocity;
- promotion of social networks, collective efficacy, social cohesion and/or social capital; or
- promotion of shared problem solving or citizen engagement.

We anticipated that policing interventions aiming to promote community connectedness will include, more generally, community consultation, partnership or collaboration with citizens and/or organisational entities. Specific strategies may include (but are not limited to) the following.

- Community meetings or forums.
- Developing partnerships with specific organisations (Fox, 2012).
- Police liaison programmes involving community members (Cherney et al., 2018).
- Police work with community leaders to enhance personal skills (e.g., self‐identity, self‐awareness and resilience), employment skills (e.g., teamwork and self‐awareness) or leadership skills (Thomas, 2019).

#### 4.2.1 Types of outcome measures

##### Primary outcomes

Terrorism is one outcome of violent extremism, which constitutes both a cognitive and behavioural component. In the literature, a distinction is made between radicalisation as constituting beliefs, while violent extremism is the behavioural outcome of those beliefs.

Hence, this review aimed to include studies where the measured outcome was violent extremist attitudes, beliefs and behaviour. For the purposes of this review, violent extremism was defined as “advocating, engaging in, preparing, or otherwise supporting ideologically motivated or justified violence to further social, economic, and political objectives” (Barker, 2015; Horgan, 2009; Khalil and Zeuthen 2016; United States Agency for International Development, 2016).

It is important to note that violent extremism is defined and captured differently across countries (e.g., Barker, 2015; Government Offices of Sweden, 2011; Lowe, 2017; Norwegian Ministry of Justice and Public Security, 2014; Public Safety Canada, 2018). In order to capture research on violent extremism across global contexts, we used either the study authors' definitions of outcomes or, in the case of ambiguity, consulted definitions used in each country's associated terrorism legislation or policies to guide the inclusion of studies with potentially eligible outcomes.

The review also aimed to include studies where the outcome was disengagement and/or deradicalization, which are often encompassed within conceptualisations of violent extremism (Klausen, Campion, Needle, Nguyen, & Libretti, 2016). Disengagement generally captures the behavioural aspect of extremism and refers to reducing or ceasing physical involvement in violent or radical activities (Horgan, 2009). In contrast, deradicalization is defined as the psychological shift in attitudes or beliefs (Horgan & Braddock, 2010). This can encompass a variety of ideologies, including: Islamist (or jihadist), far‐right (right‐wing), far‐left (left‐wing) and single issue (anti‐abortion, animal liberationists; National Consortium of the Study of Terrorism and Responses to Terrorism [START], 2018).

We included outcome data that were measured through self‐report instruments, interviews, observations and/or official data (e.g., arrests or convictions). Some examples of how violent extremism attitudes, beliefs and behaviour can be measured include the following.

- Official data taken from the Profiles of Radicalized Individuals in the United States (PIRUS),^3^ which includes: active participation in operational plots intending to cause causalities (e.g., gathering weapons, choosing targets), recruiting individuals to an official or unofficial extremist group and providing material/financial support to extremist organisations (START, 2018).
- Level of disillusionment with, disappointment with, or renouncement of extremist group members, extremist leaders and/or radical ideology (Barrelle, 2015; Berger, 2016; San, 2018).
- Willingness to engage in violence (San, 2018).
- Modification of group and personal social identity (Barrelle, 2015).
- Level of acceptance and/or engagement with cultural and religious differences or pluralistic views (Barrelle, 2015).
- Number and/or strength of ties with extremist social networks, extremist recruiters (Perliger & Pedahzur, 2011).
- Amount of extremist activity (San, 2018).

##### Secondary outcomes

No secondary outcomes were included in this review.

#### 4.2.2 Duration of follow‐up

Studies were included regardless of the length of follow‐up after the intervention. If the length of follow‐up varied across studies, we had planned to group and synthesise studies with similar follow‐up durations. For example, short (e.g., 0–3 months postintervention), medium (>3 months, <6 months) and long‐term follow‐up (>6 months post intervention). However, no studies with follow‐up data were located by the review.

#### 4.2.3 Types of settings

We aimed to include studies reporting on an impact evaluation of an eligible intervention using eligible participants, outcome(s) and an eligible research design in any setting. Where there were multiple conceptually distinct settings, we planned to synthesise the studies within the settings separately. However, there werer no variations in study settings.

We included studies written in any language that were identified by the search. We used Google Translate for the title and abstract screening stage to identify whether a non‐English language study was potentially eligible for review. Due to resource limitations, potentially eligible studies that were published in a language other than English were not translated in full to enable screening them for final eligibility. However, we used Google Translate to translate as much of the document as possible to determine final eligibility. For transparency, we include the references to any language‐other‐than‐English study that could not be unequivocally excluded using this approach in the “References to studies awaiting classification”. We included studies published between 2002 and 2018 in the review.

### 4.3 Search methods for identification of studies

The full search record for this review is provided in Appendix A in the Supporting Information Material. Searches were conducted between November 2019 and March 2020 and captured research between January 2002 and December 2018. Due to search functionalities of some websites, there was no ability to restrict searches to this date range, and so research from all publications years was assessed for eligibility.

#### 4.3.1 Electronic searches

The search for this review was led by the Global Policing Database (GPD) research team at the University of Queensland (Elizabeth Eggins, Lorelei Hine and Lorraine Mazerolle) and the Queensland University of Technology (Angela Higginson). The University of Queensland is home to the GPD (www.gpd.uq.edu.au), which served as the main search location for this review. The GPD is a web‐based and searchable database designed to capture all published and unpublished experimental and quasi‐experimental evaluations of policing interventions conducted since 1950. There are no restrictions on the type of policing technique, type of outcome measure or language of the research (Higginson et al., 2015). The GPD is compiled using systematic search and screening techniques, which are reported in Higginson et al. (2015) and summarised in Appendices B and C in the Supporting Information Material. Broadly, the GPD search protocol includes an extensive range of search locations to ensure that both published and unpublished research is captured across criminology and allied disciplines.

Because the GPD includes experimental and quasi‐experimental studies that evaluate interventions relating to police or policing, with no limits on outcome measures, we used a broad search to capture studies for the review. Specifically, we searched the title and abstracts of the corpus of GPD full‐text documents that have been classified as reporting on a quantitative impact evaluation of a policing intervention between 2002 and 2018 using the following search terms: *terror* OR extrem* OR *radical*.

#### 4.3.2 Searching other resources

We also employed additional strategies to extend the GPD search. This included the following.

- Searching trial registries (those not indexed by WHO, but listed on the Office for Human Research Protections website https://www.hhs.gov/ohrp/international/clinical-trial-registries/index.html.
- Searching counter‐terrorism organisation websites (see Table 1).
- Conducting reference harvesting on existing reviews, eligible studies and studies deemed as closely eligible for the current review.
- Forward citation searching for all eligible documents.
- Liaising with the Five Country Research and Development Network (5RD), and the Department of Homeland Security Advisory Board network for the Campbell Collaboration grants, to enquire about eligible studies that may not be publicly available.
- Personally contacting prominent scholars in the field and authors of eligible studies to enquire about eligible studies not yet disseminated or published.
- Hand‐searching the following journals to identify eligible documents published in the 12 months prior to the systematic search date that may not have been indexed in academic databases.
  a. Critical Studies on Terrorism
  b. Dynamics of Asymmetric Conflict
  c. Intelligence and Counter Terrorism
  d. International Journal of Conflict and Violence
  e. Journal for Deradicalization
  f. Journal of Policing
  g. Perspectives on Terrorism
  h. Police Quarterly
  i. Policing—An International Journal of Police Strategies and Management
  j. Policing and Society
  k. Sciences of Terrorism and Political Aggression
  l. Studies in Conflict and Terrorism
  m. Terrorism and Political Violence

**Table 1:** Grey literature search locations

Organisation	Website
Global Terrorism Research Centre (Monash University)	http://artsonline.monash.edu.au/gtrec/publications/
Triangle Centre on Terrorism and Homeland Security	https://sites.duke.edu/tcths/#
Department of Homeland Security	https://www.dhs.gov/topics
Public Safety Canada	https://www.publicsafety.gc.ca/index-en.aspx
National Consortium for the Study of Terrorism and Responses to Terrorism (START)	https://www.start.umd.edu/
Terrorism Research Centre	http://www.terrorism.org/
Global Centre on Cooperative Security	https://www.globalcentre.org/publications/
Hedayah	http://www.hedayahcentre.org/publications
RAND Corporation	https://www.rand.org/topics/terrorism.html?content-type=research
Radicalisation Awareness Network (RAN)	https://ec.europa.eu/home-affairs/what-we-do/networks/radicalization_awareness_network_en
RadicalizationResearch	https://www.radicalizationresearch.org/
Royal United Services Institute (RUSI)	https://rusi.org/
Impact Europe	http://impacteurope.eu/
National Criminal Justice Reference Service	https://www.ncjrs.gov/App/AbstractDB/AbstractDBSearch.aspx

### 4.4 Data collection and analysis

#### 4.4.1 Selection of studies

##### Title and abstract screening

After removal of duplicates and ineligible documents types (e.g., book reviews, blog posts), all records captured by the systematic search were imported into review management software, *SysReview* (Higginson & Neville, 2014). Two review authors (EE and LH) screened the titles and abstracts for all records identified by the search according to the following exclusion criteria.

1. Ineligible document type.
2. Record is not unique (i.e., duplicate).
3. Record is not about policing terrorism, radicalisation or extremism.

Prior to screening the entire corpus of search results, both screeners assessed the same small set of records and compared their judgements to verify consistent decision making. Although all efforts were made to remove ineligible document types and duplicates prior to screening, automated and manual cleaning can be less than perfect. As such, the first two exclusion criteria were used to remove ineligible document types and duplicates prior to screening each record on substantive content relevance.

Most records indexed in the GPD have a pre‐existing full‐text document. However, records from the additional searches that were deemed as potentially eligible at the title and abstract screening stage progressed to literature retrieval, where attempts were made to locate the full‐text document. Where full‐text documents could not be retrieved via existing university resources, they were ordered through the review authors' university libraries. All potentially eligible records then progressed to full‐text eligibility screening. If the full‐text document could not be located, the abstract was used to assess whether the study met full‐text eligibility criteria. Where a decision could not unequivocally made about eligibility based on the abstract, the record was categorised as a study awaiting classification (see “References to studies awaiting classification” section).

##### Full‐text eligibility screening

The same two review authors (EE and LH) also screened the full‐text for potentially records for final eligibility according to the following exclusion criteria.

1. Ineligible document type.
2. Document does not evaluate a policing intervention that aims to promote community connectedness.
3. The evaluation does not report violent extremism attitudes, beliefs or behaviour as an outcome.

All efforts were made to remove ineligible document types in earlier stages. However, sometimes these types of records can progress into later stages of screening (e.g., where duplicate records are not adjacent during screening or where screeners cannot unambiguously determine whether a record is ineligible based on the title and abstract). Therefore, the first two exclusion criteria were used to remove ineligible document types and duplicates.

#### 4.4.2 Data extraction and management

Two review authors (EE and LH) independently coded and extracted data from eligible studies within *SysReview*, using the coding form provided in Appendix D in the Supporting Information Material. Disagreements or inconsistencies were resolved by discussion with a third review author (AH). Broadly, studies were coded according to the following domains.

1. General study characteristics (e.g., document type and study location).
2. Participants (e.g., sample characteristics by condition).
3. Intervention (e.g., intervention components, intensity andf setting).
4. Outcomes (e.g., conceptualisation, mode of measurement and time‐points).
5. Research methodology (e.g., design, unit and type of assignment).
6. Effect size data.
7. Risk of bias.

#### 4.4.3 Assessment of risk of bias in included studies

Two review authors (EE and LH) assessed risk of bias using the Cochrane Risk of Bias in Non‐Randomised Studies‐Interventions tool (ROBINS‐I), which guides rating across seven domains to determine low, moderate, serious, or critical risk of bias, or no information to make a judgement (Sterne et al., 2016). The *confounding* domain assesses whether the study accounts for the baseline and/or time‐varying prognostic factors (e.g., socioeconomic status). The *selection* domain refers to biases internal to the study in terms of the exclusion of some participants, outcome events, for follow‐up of some participants that is related to both intervention and outcome. The *classification of interventions* domain refers to differential (i.e., related to the outcome) or nondifferential (i.e., unrelated to the outcome) misclassification of the intervention status of participants. The *measurement of outcomes* domain assesses whether bias was introduced from differential (i.e., related to intervention status) or nondifferential (i.e., unrelated to intervention status) errors in the measurement of outcome data (e.g., if outcome measures were assessed using different methods for different groups). The *deviations from intended interventions* refers to differences arising in intended and actual intervention practices that took place within the study. The *missing data* domain measures bias due to the level and nature of missing information (e.g., from attrition, or data missing from baseline or outcome measurements). Finally, the *selection of reported results* domain is concerned with reporting results in a way that depends on the findings (e.g., omitting findings based on statistical significance or direction of effect). The results of the risk of bias assessment are provided in a written summary and table. If future updates of the review identify additional eligible studies, the results of the risk of bias assessment will also be depicted in a risk of bias summary figure.

#### 4.4.4 Measures of treatment effect

There was only one eligible study, in which all outcome data were collected from individual participants, using continuous measures. The specific data required to calculate effect sizes were not included in the eligible study reports, but was provided by the study authors. Means, standard deviations and sample size for each group was used to calculate standardised mean differences (SMD) in *RevMan*, along with 95% confidence intervals.

#### 4.4.5 Unit of analysis issues

Unit of analysis issues can arise where (a) multiple documents report on a single empirical study; (b) multiple conceptually similar outcomes are reported in the one document; (c) data are reported for multiple time‐points; and/or (d) studies have clustering in their research design. Based on the research included in this review, none of these issues were relevant when quantifying and synthesising treatment effects. Although the single included study was reported in two documents, only one document reported quantitative data and sufficient detail to enable data extraction. For future updates of this review, our approach for handling these issues is specified in the review protocol (Mazerolle et al., 2020).

#### 4.4.6 Dealing with missing data

Where data were missing in relation to coding categories, study authors were contacted by email to obtain the data. The results section also specifies which data were obtained from published reports of a study and which data were obtained directly from study authors (not available in the public domain).

#### 4.4.7 Assessment of heterogeneity

Due to the limited research included in this review, we were unable to assess heterogeneity. However, for updates of this review, our approach for handling these issues is specified in the review protocol.

#### 4.4.8 Assessment of reporting biases

Due to the limited research included in this review, we were unable to assess reporting/publication biases. However, for updates of this review, our approach for handling these issues is specified in the review protocol.

#### 4.4.9 Data synthesis

Due to the limited research included in this review, we were unable to conduct meta‐analyses. Rather, estimates of treatment effects for eligible outcomes are presented as single SMDs, with their corresponding confidence intervals. For updates of this review, our approach for handling these issues is specified in the review protocol.

#### 4.4.10 Subgroup analysis and investigation of heterogeneity

We had planned to use subgroup analyses to assess whether the impact of the intervention varied by the following factors: geographical location, target population and type of policing strategy used to promote connectedness. However, due to the limited research included in this review, we were unable to conduct subgroup analyses. For updates of this review, our approach for conducting subgroup analyses is specified in the review protocol.

#### 4.4.11 Sensitivity analysis

We had planned to use sensitivity analyses to assess the impact of risk of bias on estimates of the treatment effect. However, due to the limited research included in this review, we were unable to conduct these analyses. For updates of this review, our approach for conducting sensitivity analyses is specified in the review protocol.

### 4.5 Deviations from the protocol

Our review made six minor deviations from the protocol. First, despite intending to translate potentially eligible documents written in languages other than English, resources did not permit this approach. However, for transparency, the “References to studies awaiting classification” section lists all studies written in another language that could not be unequivocally excluded on their titles and/or abstracts or by attempts to translate documents via Google Translate.

Our second deviation was changing the hand searches of journals to 12 months prior to the end search date within the GPD and grey literature sources (i.e., December 2018). The protocol stated that the hand search would encompass the most recent four issues of each of the listed journals; however, conducting the hand search in this way would have included a nonequivalent sample of research (i.e., 2019 data, which was not captured by the remainder of the systematic search).

Our third deviation was using the full‐text eligibility criteria to screen the titles and/or abstracts of records that were retained after title and abstract screening, but where a full‐text could not be sourced to determine final eligibility. Records were only excluded in this way if an unequivocal decision could be made, otherwise, they were retained and included in the “References to studies awaiting classification” section.

Our fourth deviation was not contacting study authors where there was either missing information or “unclear” ratings during the risk of bias assessment. We chose this approach as the additional information would not have changed the overall risk of bias result. However, for updates of the review, we will follow the original protocol.

The fifth deviation was slightly changing the wording of the title and abstract screening criteria. In the protocol, the third criterion was “Document is not about terrorism or extremism”. While this wording was appropriate for records extracted from the GPD search because all records were impact evaluations relating to police or policing, this approach was overly sensitive for results captured by the grey literature and hand searching. Therefore, we changed the third title and abstract screening criterion to “Document is not about policing terrorism, radicalisation or extremism”.

The final deviation to the protocol is adding additional search steps in light of the low number of eligible studies identified by the systematic search and screening process. Specifically, we harvested the reference lists of excluded studies that were deemed to almost meet inclusion criteria (e.g., eligible intervention, no evaluation or ineligible outcomes) or that contained substantive content that was closely aligned with the review topic.

## 5 RESULTS

### 5.1 Description of studies

#### 5.1.1 Results of the search

**Figure 1:**
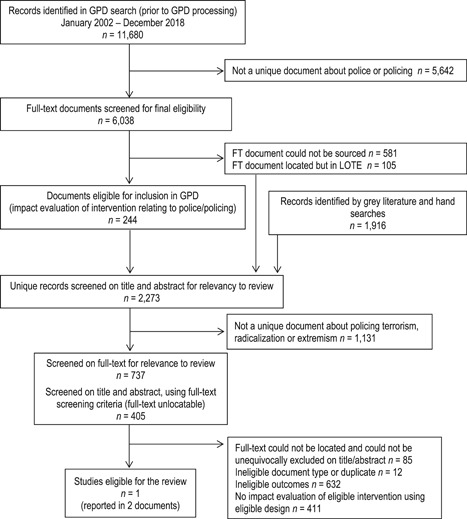
Preferred Reporting Items for Systematic Reviews and Meta‐Analyses flowchart

The results of search and subsequent screening are summarised in Figure 1. The search within the overall GPD systematic search identified 11,680 prior to any systematic processing that underpins the GPD (see Appendices B and C in the Supporting Information Material). Of these 6,038 were screened as being potentially about police or policing on their titles and abstract. A total of 5,352 full‐text English documents were located, with 686 unable to be located or written in a language other than English. Of the located full‐texts, 244 were screened as reporting on a quantitative impact evaluation of an intervention relating to police or policing, and deemed eligible for the GPD. For completeness, the GPD search component of this review includes both these GPD‐eligible studies and the records that were screened as being about police or policing on their title and abstract, but where a full‐text could not be located or was written in a language other than English. The GPD search results were combined with the records identified by the grey literature search, hand searches, reference harvesting and forward citation searching (*n* = 1,916) to generate a corpus of 2,273 records (after preliminary duplicate removal).

A total of 1,142 records were screened on their title and/or abstract as being potentially about policing terrorism, radicalisation or extremism. We obtained the full‐text of 737 of these records via institutional libraries or correspondence with document authors, of which 71 were written in a language other than English. The full‐text for the remaining 405 records could not be located via institutional libraries or through contact with document authors (including *n* = 50 written in a language other than English). Of these, almost half were conference presentations or magazine articles, with the rest of evenly distributed amongst the following categories of document types: journal articles, books or book chapters, reports or working papers (government and technical), and theses. We handled the processing of records with no full‐text in two stages. First, we screened their titles and abstracts using the more specific full‐text screening criteria, which resulted in the exclusion of 313 records. These are recorded separately to the studies excluded on their full‐text in the “References to excluded studies” reference list. Second, we attempted to contact the authors of the remaining 92 records where we could not unequivocally exclude the record by screening the title and abstract using the full‐text screening criteria. Seven authors responded without providing a full‐text document, but confirmed that their research did not meet the review inclusion criteria. For the remaining 85 records: (a) authors could not provide the full‐text or recall whether the document met inclusion criteria; (b) no response was received from document authors; or (c) contact details for document authors could not be found. These documents are reported in the “References to studies awaiting classification” list. Of the 1,057 studies screened on the full‐text screening criteria, only one study met the review eligibility criteria (reported in two documents).

#### 5.1.2 Included studies

The sole eligible study by Williams, Horgan, and Evans (2016) was conducted in Montgomery County, Maryland, United States in 2015. The study evaluated the impact of the World Organisation for Resource Development Education (WORDE) programme on violent extremism attitudes, beliefs and behaviours. This programme was a nonprofit organisation funded by the National Institute of Justice and led by a group of Muslim scholars and community leaders. The group also worked with experts in the areas of policy analysis, theology, academia and development. The programme was multipronged with three “interlocking” components. First, the programme provided community education with a town hall meeting component that included dialogue opportunities for public officials and members of the public. The educational component covered topics such as family support, youth engagement and conflict resolution. Second, the programme sought to enhance the capacity of agencies to create a referral network to identify and help individuals who are at risk of becoming radicalised or are at risk of committing violent offences. Agencies involved in this component of the intervention included law enforcement, community organisations and social services (e.g., social workers, psychologists). Third, the programme provided community members the opportunity to participate in organised volunteerism and/or multicultural activities, such as art projects around social change and work to assist the homeless. This component of the intervention did not explicitly involve law enforcement.

Participants were eligible for inclusion within this study if they resided in Montgomery County, Maryland. The WORDE programme targeted youth and adults who were civically engaged, sensitised to issues of violent extremism and who had existing cooperative relationships with law enforcement and social services. The programme aimed to foster and maintain these networks for participants. Treatment participants (*n* = 133) were invited to participate in the study by WORDE programme staff and a pre‐screening questionnaire was used by researchers to generate a stratified random sample of participants. Comparison participants (*n* = 58) were recruited through (a) interfaith partners not involved in WORDE; (b) public school list‐serves; and (c) electronic bulletin boards (e.g., Craigslist, Google Groups). The comparison group were considered to be engaged with other volunteerism or multicultural events and activities, but not with the specific WORDE programming.

The authors report that the groups were matched using propensity score matching using demographic characteristics age, race, religion and educational level; however, it is unclear whether this was implemented as intended. The groups were predominantly male (treatment = 75.9%; comparison = 51.7%) and aged in their midtwenties (treatment *M* = 26.54 years, comparison *M* = 27.33), and Caucasian (treatment = 91.7%, comparison = 57.9%).

Williams et al. (2016) assessed the WORDE intervention on behavioural outcomes (e.g., increased coping skills), attitudes (e.g., towards different religions or ethnicities) and knowledge of out‐group cultures. Self‐report surveys captured these outcomes at one time point (after an unknown period of engagement with the programme^4^) using a 14‐item measure constructed by the authors, the *Brief Volunteer Program Outcome Assessment*. Items were measured on a seven‐point Likert scale (“completely disagree” to “completely agree”) with higher mean values equating to more agreement with the statement. The specific items were as follows.

“Thinking of when you volunteer, please rate your level of agreement with the following statements:

- I feel welcome*
- I feel a part of something bigger than myself*
- I feel a sense of teamwork*
- I make friendships that are active beyond the event*
- I make friends with people from other races*
- I feel useful
- I have responsibilities
- I have leadership responsibilities
- I feel a sense of purpose
- I feel free of peer pressure*
- I feel accepted*
- I wouldn't feel lonely
- I wouldn't feel afraid to talk to others*
- I learn about cultures other than my own*” (p. 157)

Of the above items, nine were deemed as eligible outcomes (marked with asterisk) and were considered to fall under the general banner of deradicalization. Specifically, these items align with Barrelle's (2015) conceptualisation that is defined as the level of acceptance and/or engagement with cultural and religious differences or pluralistic views and modification of group or personal identity. It is important to note that it is not explicitly clear whether the evaluation participants in the treatment group were all directly exposed to the two intervention components that involved police. Hence, these evaluation outcomes may not be direct measures of how effective police were at countering violent extremism by promoting community connectiveness.

#### 5.1.3 Excluded studies

Due to the number of records screened for final eligibility (*n* = 1,057), we are unable to describe the full body of excluded studies (see “References to excluded studies” section). Rather, this section describes a small set of studies that almost met the review inclusion criteria or described interventions closely aligned with the review topic (*n* = 29). A total of 24 were documents that contained substantive content about community‐oriented policing approaches for countering terrorism, extremism or radicalisation but that did not report on an original impact evaluation (Barclay, 2011; Warnes, 2016; McGarrell, Freilich, & Chermak, 2007; Miller, Toliver, & Schanzer, 2016; Radicalisation Awareness Network, 2016a, 2016b; Pisoiu, 2018; Public Safety Canada, 2013, 2018; Helmus et al., 2017; Hofman & Sutherland, 2018; Lenos & Keltjens, 2016a, 2016b, Schanzer et al., 2016; Schwartz, 2015; U.S. Department of Homeland Security, 2011, 2015a, 2015b; Appelboom, 2015; Briggs, 2010; Silk, Spalek, & O'Rawe, 2013; Weine, Polutnik, & Younis, 2015; Cherney et al., 2018; DuBois & Alem, 2017).

Example intervention approaches included the following.

- *Connect Training Programme*: Police and community members participate together in training around understanding community policing from the other's perspective and building positive relationships (Irvine, 2013, reported in Silk et al., 2013).
- Framing activities around COMPLETE Public Safety (COMmunity Partnerships with Law Enforcement To Enhance Public Safety), rather than countering violent extremism (Schanzer et al., 2016).
- *Living Safe Together*: Provided government funding for multiple community activities, including camps for Muslim youth involving police, and community leaders whereby they received educational talks as well as opportunities to form relationships with both the police officers and their peers (Cherney et al., 2018).
- *More than a Game*: Community‐based resilience partnership between police and an Australian Rules football club, which targeted Muslim youth. Comprised sporting activities (e.g., football, cricket, surfing), police mentoring around social skills and leadership, and police workshops (on topics such as cyberbullying, counter‐terrorism, conflict resolution; DuBois & Alem, 2017).
- *Edmonton Resiliency Project*: Collaborative intervention delivered by City of Edmonton, Edmonton Police Service and the Organisation for the Prevention of Violence. Conducts education, engagement and offline and online interventions (Public Safety Canada, 2018).
- Multiple community policing activities in Amhara, Ethiopia, focus on trust‐building, including organising watch patrols, adjudicating minor disputes, literacy training, public health and family education (Schwartz, 2015).
- Hotlines or online reporting for community to notify police of concerns relating to possible terrorist activity (Briggs, 2010).
- Engaging the community in dialogue around countering violent extremism via social media (Schanzer et al., 2016).

In addition, two studies reported on impact evaluations of eligible interventions but used ineligible outcomes to assess the intervention's effectiveness. For example, Aksu (2014) evaluated the impact of nine community policing activities (e.g., family visits, sporting events, visits to police units) within a counter‐terrorism context in Turkey using an unmatched control group on citizen perceptions of police procedural justice and legitimacy (see also Cherney & Murphy, 2017).

A sample of three studies also examined eligible intervention approaches but did not evaluate these using eligible research designs (Boyd‐MacMillan, 2016; Weine, Eisenman, Kinsler, Glik, & Polutnik, 2017; Pickering, Wright‐Neville, McCullouch, & Lentini, 2007). For example, Boyd‐MacMillan (2016) evaluated Being Muslim Being Scottish, a multi‐session skills course with participants from both the community and practitioners (e.g., police, social workers) using a single group pre/post research design on participants’ integrative complexity regarding views towards their self‐reported own and opposed communities. Alternatively, (Weine et al. 2015; Weine, Younis, & Polutnik, 2017) conducted a process evaluation of countering violent extremism tailored community policing activities conducted by the Los Angeles Police Department, which included a biannual police‐Muslim community organisation forum.

### 5.2 Risk of bias in included studies

Table 2 summarises the degree of bias and specific reasons for this rating across the seven domains, based on the standardised questions provided by the ROBINS‐I tool. Specifically, the study was rated as having serious risk of bias for the confounding, selection, classification of interventions, and measurement of outcomes domains. A lack of information or ambiguity in the available reports for the study or data provided by study authors led to a rating of “no information” for the deviations from intended interventions and the missing data domains. Finally, the selection of reported results was rated as having a moderate risk of bias. Overall, these ratings suggest that the single included study included in the review has a relatively serious risk of bias.

**Table 2:** Results for risk of bias assessment

Risk of bias domain	Rating	Rationale
Confounding	Serious	No baseline assessment of outcome measures or other potential confounders that might influence engagement with the treatment or comparison condition activities. In addition, it is unclear if authors verified if comparison participants had no interaction with the treatment (World Organisation for Resource Development Education [WORDE] programme), which raises the risk of time‐varying confounding because participants may have received both interventions. While the authors state that propensity score matching was used, their published report and data provided by the authors for the purposes of this review do not allow for independent assessment of the propensity score matching. Specifically, the variables the authors use to “match” participants vary within the report are not clear. For example, one section states participants were matched on demographics and another section states the participants were matched on nine measures including: trust in police, political extremism, racism, resiliency and coping, emotional stability, amped political extremism, historical loss, religiosity, and religious dogmatism. The time of measurement for these potential confounders is also not clear (e.g., whether at the beginning and end of the intervention).
Selection	Serious	Participants were invited to participate in the evaluation after they had already begun engagement with the WORDE programme (treatment) and the treatment group was based on a stratified random sample from those who expressed interest in the study. One of the variables influencing selection included the frequency of attendance. Although authors sampled across regular, infrequent and “one‐timer” attendees, it is unclear how attendance might impact estimates of treatment effect.
Classification of interventions	Serious	Intervention and comparison group not clearly defined. Specifically, it is unclear whether authors verified whether comparison group participants had any contact or engagement with the WORDE programme. The specific treatment received by comparison group participants is not clearly articulated aside from other multicultural events or volunteerism activities. It is also unclear whether the information used to define the groups was recorded at the start of the intervention or after the intervention and evaluation was underway.
Deviations from intended interventions	No information	The intended intervention and actual implementation of intervention were reported by study authors. The authors outlined the aims and activities of WORDE programme, but do not discuss implementation challenges or changes made to implementation. The reported variation in attendance for WORDE participants is suggestive of adherence issues, but this is not unexpected in community interventions and the content of the intervention (countering violent extremism).
Missing data	No information	The published report of the study does not provide participant demographics. Data supplied by study authors suggests there was no attrition, but this could be not be independently verified.
Measurement of outcomes	Serious	Outcome measurement appears to be equivalent across groups. However, study authors developed the outcome measures based on interviews with WORDE participants (treatment group) and it is not clear whether the same participants are used for the evaluation and design of the instrument. In addition, the way participants answered the survey questions may have been influenced by their knowledge of and self‐selection into the treatment and evaluation.
Selection of reported result	Moderate	No prospectively published protocol for the study exists. The published report for the study does not provide the results of the between group analyses, but rather reports data for the treatment group and a statement that there were no statistically significant differences between the treatment and comparison group. Given this statement about lack of differences, it is unlikely that the reported effects or data provided by study authors for this review are selected on the basis of the results from multiple outcome measurements within the one domain, multiple analyses of the intervention‐outcome relationship, or different subgroups. Data was provided by authors upon request.

### 5.3 Synthesis of results

Table 3 provides the standardised mean differences (SMDs) and their associated 95% Confidence Intervals for each eligible self‐report survey item from the Williams et al. (2016) study. All effect sizes were calculated with RevMan using data provided directly by the study authors, specifically: propensity‐matched means, standard deviations, and number of participants in each group. Overall, the effect sizes were small to medium in size and favoured the treatment group, except for one item which favoured the comparison group (survey item: “*I make friends with people from other races*”). However, all but three of the SMDs have confidence intervals that include zero, which indicates a lack of statistically significant differences between treatment and comparison group participants for most survey items.

**Table 3:** Impact of World Organisation for Resource Development Education programme on self‐report deradicalization outcomes

Survey item	Standardised mean difference	95% Confidence interval
I feel welcome	0.47	0.15 to 0.78
I feel part of something bigger than myself	0.11	−0.20 to 0.42
I feel a sense of teamwork	0.24	−0.07 to 0.55
I make friendships that are active beyond the event	0.21	−0.10 to 0.52
I make friends with people from other races	−0.51	−0.82 to −0.19
I feel free of peer pressure	0.88	0.56 to 1.20
I feel accepted	0.44	0.13 to 0.75
I wouldn't feel afraid to talk to others	0.44	0.13 to 0.75
I learn about cultures other than my own	0.25	−0.06 to 0.56

## 6 DISCUSSION

### 6.1 Summary of main results

Police programmes to tackle violent extremism can involve a range of approaches and partnerships. One approach includes efforts to improve community connectedness by working to address social isolation, belonging, economic opportunities, and norms and values that may lead people to endorse or support violent extremist causes and groups. The assumption is that the risk of an individual being radicalised in the community can be reduced when police work with community members and groups to mobilise and support activities that help generate a sense of belonging and trust. Police programmes that build a sense of belonging and trust may help ensure individuals are not influenced by activities that violent extremists use to attract support for their cause.

Our review identified 24 studies that nearly met the inclusion criteria but were ultimately excluded because they did not use rigorous experimental or quasi‐experimental evaluation methods. These studies are summarised in this report and indicate a wide array of practices that are being adopted by police in their efforts to improve community connectedness as a way to tackle violent extremism.

Our review only identified one study that met our inclusion criteria. The included study was conducted in the United States by a Muslim‐led organisation in partnership with police and other agencies. The programme aimed to counter violent extremism through an education and awareness campaign and by improving networks for agencies/third parties to help assist individuals identified as at‐risk of radicalisation. The awareness raising activities aimed to increase knowledge and understanding about violent extremism risk factors that would help to facilitate problem solving between police and community groups to better to identify risks within the community. Increasing prosocial linkages or ties between agencies, community members and police was aimed at ensuring these stakeholders could better work together when identifying and coming into contact with radicalised individuals. The purpose was to help generate greater involvement in responses to terrorism across various stakeholders. The included study used a quasi‐experimental design to compare programme participants to non‐participants on a range of self‐report measures comprised of items representative of deradicalization. Of the nine effect sizes calculated, all favoured the treatment group except for one which favoured the comparison group (survey item: “*I make friends with people from other races*”). The effect sizes were small to medium in size and only three did not have confidence intervals that included zero. It is important to note that it is not explicitly clear whether the evaluation participants in the treatment group were all directly exposed to the two intervention components that involved police. Hence, these evaluation outcomes may not be direct measures of how effective police were at countering violent extremism by promoting community connectiveness. Overall, our review demonstrates that there is a global lack of rigorous evaluation evidence to conclusively determine whether or not police‐involved programmes to tackle violent extremism through promoting community connectedness are effective.

### 6.2 Overall completeness and applicability of evidence

Just one study was identified that met our inclusion criteria. This study was undertaken in State of Maryland in the United States, which significantly limits our capacity to draw any generalisable conclusions from the results nor conduct any analysis of publication bias. The fact that we only identified one study also prevented our capacity to answer our secondary research question which was to identify whether effectiveness varied by the intervention type and location. It was not possible to assess the differential impact of policing interventions that target either individuals or places (either micro or macro).

The outcome measures included in the one eligible study were limited to self‐report attitudinal measures, which also limits our capacity to examine the full breadth of outcomes, including: direct behavioural outcomes representative of violent extremism, different components theorised to fall within radicalisation and disengagement, and outcomes measures in different modalities such as official administrative data. In addition, self‐reporting measures may increase the potential for bias specifically in relation to radical attitudes, behaviours or beliefs. Due to the high‐risk nature of this behaviour participants are less likely to answer on a truthful basis, especially if they do espouse radical beliefs and attitudes that could escalate to violence. Finally, the single included study was a multicomponent intervention whereby not all intervention components explicitly had police involvement. Therefore, any effects of the intervention on the measured outcomes may not be directly attributable to police efforts to enhance community connectedness.

### 6.3 Quality of the evidence

The single included study in this review was rated as having serious risk of bias, using the ROBINS‐I tool. The overall quality of evidence on the effects of police programmes to tackle violent extremism through efforts to improve community connectedness is weak and highly limited. Existing studies do not adopt randomised or rigorous experimental methods. While tackling the problem of violent extremism through improving community connectedness is argued as having merit and value in the existing literature (Cherney & Hartley, 2017; Murray et al. 2015; Pickering, McCulloch, & Wright‐Neville, 2008; Ramiriz, Quinlan, Malloy, & Shutt, 2013), the current review did not locate sufficient evidence to substantiate that police efforts in this area have an impact. We note that more randomised and rigorous quasi‐experiments with matched control groups/areas would improve the quality of evidence on police programmes to counter violent extremism by improving community connectedness.

Methods for evaluating counter‐terrorism initiatives can be particularly challenging due to the hard‐to‐reach nature of the population. In addition to the lack of formal evaluations, programmes that target violent extremism attitudes, beliefs and behaviours often lack appropriate methods and practices to measure impact or effectiveness. Programmes that adopt clear inclusions criteria and collect baseline data (Holdaway & Simpson, 2018) could increase the likelihood of formal evaluations on the impact of police and community connectedness on violent extremism. The existing body of evidence is largely a function of the fact few programmes in this area have been subject to any type of formal evaluation.

### 6.4 Limitations and potential biases in the review process

In general, there are no specific limitations or biases in the systematic review process. The use of the GPD is a significant asset to the current review. The GPD is a web‐based and searchable database designed to capture all published and unpublished experimental and quasi‐experimental evaluations of policing interventions conducted since 1950. There are no restrictions on the type of policing technique, type of outcome measure or language of the research (Higginson et al., 2015). The GPD is compiled using systematic search and screening techniques, which are reported in Higginson et al. (2015) and summarised in Appendices B and C in the Supporting Information Material. Broadly, the GPD search protocol includes an extensive range of search locations to ensure that both published and unpublished research is captured across criminology and allied disciplines. The systematic search and screening underpinning the GPD thus provides a robust basis for expediting systematic reviews related to police and policing. This particular review was enhanced further by additional search steps to identify grey literature or studies that may not have been indexed in the GPD.

One limitation of the review is that the review includes research published by December 31, 2018. The evaluation of policing interventions aimed at enhancing community connectedness to counter violent extremism may be a rapidly moving area of research, which may mean this review omits eligible studies conducted in 2019. Therefore, it will be important that this review is updated within 2–3 years to capture any new research, which will also facilitate more concrete conclusions about the effectiveness of policing interventions aimed at enhancing community connectedness to counter violent extremism.

### 6.5 Agreements and disagreements with other studies or reviews

Due to the limited nature of evaluation and review literature on the effectiveness of policing approaches aimed at enhancing community connectedness to counter violent extremism, the findings of this review do not reaffirm or contradict any existing review. However, this review does provide a framework for assessing future research, which will be captured in updates of this review.

## 7 CONCLUSIONS

Currently there exists little evidence to evaluate whether police efforts to counter violent extremism through promoting community connectedness are effective. However, this conclusion needs to be considered in light of the fact that programmes to counter violent extremism are very challenging to implement and evaluate, and the outcomes reported in this review are the result of the low investment made in rigorous evaluations, a problem recognised in the field more generally (Hofman & Sutherland, 2018).

### 7.1 Implications for practice and policy

Despite the findings of this review, further research is needed to ascertain whether police efforts to enhance community connectedness can counter violent extremism attitudes and beliefs. With the lack of an existing evidence base, a key question to be raised is the role of the police in such community‐based efforts. For instance, existing scholarship draws attention to the downside of counter‐terrorism community engagement with the Muslim community, which has seen police prioritise intelligence gathering on terrorist threats over more collaborative efforts that aim to address issues of concern to Muslim community members (Cherney & Hartley, 2017). Moreover, since 9/11, the war on terror has sometimes led to a deterioration in relations between the police and the Muslim community due to perceptions of police unfairly targeting Muslims (Cherney & Murphy, 2017). This erodes trust towards and cooperation with the police. This does not mean police should play no role in strategies to enhance community connectedness to tackle violent extremism. Yet it does raise the issue as to what precisely should be the role of police in managing complex issues such as violent extremism within their own communities (see Meares & Tyler, 2020).

Police often come into contact with individuals only after they have engaged in radical actions or behaviours. This suggests that police could be well placed to cultivate communities of “intimates” around those that are exhibiting radical characteristics (see Grossman, Stephenson, Street, & Zhang, 2015). Family members, close friends and community insiders are best placed to detect signs of change in thinking and behaviour and as Grossman and her colleagues (Grossman et al., 2016; Thomas, Grossman, Miah, & Christmann, 2017) suggest, there is much to be gained by police doing much more than they presently do to encourage community reporting of violent extremism. Hence, police involvement in efforts to build trust and connectedness within communities through organisations such as places of worship and educational institutions, can inspire trust and promote the willingness of friends, family members or known members of the community to work with police.

This highlights the importance of promoting community‐based approaches. However, currently we have limited evidence to help guide policy and practice around what types of interventions work to reduce violent extremist behaviours, attitudes and beliefs. Whilst we know that community policing strategies have positive effects on citizen satisfaction, perceptions of disorder, and police legitimacy (see Gill et al., 2014) our review demonstrates the need for more investment in the careful design and evaluation of the types of policing initiatives that might help promote social connectedness to reduce violent extremism behaviour, attitudes, and beliefs. This type of investment is needed to better inform policymakers and practitioners on how to both generate evidence‐based knowledge and inform the translation and implementation of best‐practises in countering violent extremism.

### 7.2 Implications for research

Conducting evaluation research in the area of countering violent extremism is particularly difficult (Koehler, 2017; Romaniuk & Chowdhury Fink, 2012). This review clearly demonstrates that far more rigorous evaluation research is required to ascertain whether policing programmes aiming to promote community connectedness are an effective approach for countering violent extremism behaviours, attitudes, and beliefs.

## CONFLICT OF INTERESTS

Three of the review authors have internal roles within the Campbell Collaboration Crime and Justice Group. Lorraine Mazerolle is the Co‐Chair of the Crime and Justice Coordinating Group (CJCG), Angela Higginson is the Editor of the CJCG and Elizabeth Eggins is the Managing and Associate Editor of the CJCG. Consequently, Lorraine Mazerolle, Angela Higginson and Elizabeth Eggins were not involved in any editorial or internal Campbell Collaboration communications about this review. In addition, Adrian Cherney has published research that is closely linked with the review topic. To minimise potential bias, Adrian Cherney was not involved in the screening or coding of any studies for this review. The other authors declare that there are no conflict of interests.

## AUTHOR CONTRIBUTIONS

All the authors developed the content. E. E., A. H., and L. H. contributed to systematic review methods. E. E. and A. H. contributed to statistical analysis. E. E., A. H., and L. H. contribted to information retrieval. Lorraine Mazerolle is a Professor of Criminology in the School of Social Science at the University of Queensland and current Co‐Chair of the Campbell Collaboration Crime and Justice Coordinating Group. She has won numerous US and Australian competitive research grants, including systematic reviews, on topics such as third‐party policing, police engagement with high‐risk people and disadvantaged communities, community regulation, problem‐oriented policing, police technologies, civil remedies, street‐level drug enforcement and policing public housing sites. Elizabeth Eggins is the Managing and Associate Editor of the Campbell Collaboration Crime and Justice Coordinating Group. Her research focuses on intervention evaluation through randomised experiments and systematic reviews, with a broad focus on crime and justice and social welfare. She has coauthored and managed numerous systematic reviews and she has particular expertise in systematic review methodology, analysis and information retrieval. Adrian Cherney is a Professor in the School of Social Science at the University of Queensland and an Australian Research Council (ARC) Future Fellow. His current research focuses on the evaluation of programmes aimed at countering violent extremism and he has undertaken research on the supervision of terrorist offenders in Australian who have been released into the community on parole. His ARC Future Fellowship aims to develop and test metrics and methods to evaluate case‐managed interventions targeting individuals who have been charged for a terrorist offence or have been identified as at risk of radicalising to violent extremism. He has secured both international and national competitive grants. Lorelei Hine is a Senior Research Assistant in the School of Social Science at the University of Queensland. She has assisted with the project management of several systematic reviews and comanages the Global Policing Database. This has provided her with expertise in both systematic review methodology and substantive content in relation to criminal justice interventions. Angela Higginson is a Senior Lecturer in the School of Justice, Faculty of Law, QUT and current editor of the Campbell Collaboration Crime and Justice Coordinating Group. She is an ARC Discovery Early Career Research Award (DECRA) fellow for 2018–2020, and her DECRA project examines the correlates and consequences of ethnically‐motivated youth hate crime in Australia. Much of her work has focused on policing and community processes for crime control, with a particular expertise in evaluation through systematic reviews and meta‐analysis. Emma Belton is a PhD student and Research Assistant in the School of Social Science at the University of Queensland. She has coauthored and worked on projects in the area of countering violent extremism (CVE), including the collection and analysis of data and evaluations of programmes aimed CVE.

## Supporting information

Supporting information

## Footnotes

1 We conducted a search of the literature using the following terms to identify existing reviews: terroris* OR extremis* OR radicali*. Searches of the Campbell Collaboration library, Cochrane Collaboration library, PROSPERO register and Google Scholar did not identify any existing systematic reviews (completed or ongoing) on the specific review.

2 We will include all short interrupted time series designs with control group, as long as the design includes a minimum of one preintervention observation for each of the treatment and comparison groups. This approach is consistent with the inclusion of unmatched control group designs with pre‐post intervention measures. For studies with extremely short preintervention time series (less than 4 pre‐ and 4 post‐intervention observations), the data will be collapsed and treated as pre‐post averages, rather than as true time series data.

3 We use the term “official data” loosely here as there is limited official data or direct measures of violent extremism. PIRUS stipulates strict inclusion criteria of individuals who have been radicalised or radicalised to violence.

4 Information provided via personal correspondence with study authors.

## References

[cl21111-bib-0001] Ahmed, K. , Belanger, P. , & Szmania, S. (2018). Community‐focused counter‐radicalization and counter‐terrorism projects: Experiences and lessons learned. Lanham, MD: Lexington Books.

[cl21111-bib-0002] Mirahmadi, H. (2016). Building resilience against violent extremism: A community‐based approach. The Annals of the American Academy of Political and Social Science, 668(1), 129–144. 10.1177/0002716216671303

[cl21111-bib-0003] Williams, M. J. , Horgan, J. G. , & Evans, W. P. (2016). Evaluation of a multi‐faceted, US community‐based, Muslim‐led CVE program (Report No. 249936). Washington, DC: U.S. Department of Justice. Retrieved from https://www.ncjrs.gov/pdffiles1/nij/grants/249936.pdf

[cl21111-bib-0004] Akchurina, V. , & Lavorgna, A. (2014). Islamist movements in the Fergana Valley: A new threat assessment approach. Global Crime, 15(3‐4), 320–336. 10.1080/17440572.2014.924406

[cl21111-bib-0005] * Aksu, G. (2014). *Winning hearts and minds in counterterrorism through community policing and procedural justice: Evidence from Turkey* (Doctoral dissertation). Retrieved from ProQuest Dissertations and Theses Global database (UMI No. 3629006).

[cl21111-bib-0006] Aldrich, D. P. (2014). First steps towards hearts and minds? USAID's countering violent extremism policies in Africa. Terrorism and Political Violence, 26(3), 523–546. 10.1080/09546553.2012.738263

[cl21111-bib-0007] Alex, P. S. (2013). *Radicalisation, de‐radicalisation, counter‐radicalisation: A conceptual discussion and literature review* (ICCT Research Paper). The Hague, The Netherlands: International Centre for Counter‐Terrorism.

[cl21111-bib-0008] Alison, L. J. , Alison, E. , Noone, G. , Elntib, S. , & Christiansen, P. (2013). Why tough tactics fail and rapport gets results: Observing Rapport‐Based Interpersonal Techniques (ORBIT) to generate useful information from terrorists. Psychology, Public Policy, and Law, 19(4), 411–431. 10.1037/a0034564

[cl21111-bib-0009] Alison, L. , Alison, E. , Noone, G. , Elntib, S. , Waring, S. , & Christiansen, P. (2014a). The efficacy of rapport‐based techniques for minimizing counter‐interrogation tactics amongst a field sample of terrorists. Psychology, Public Policy, and Law, 20(4), 421–430. 10.1037/law0000021

[cl21111-bib-0010] Alison, L. , Alison, E. , Noone, G. , Elntib, S. , Waring, S. , & Christiansen, P. (2014b). Whatever you say, say nothing: Individual differences in counter interrogation tactics amongst a field sample of right wing, AQ inspired and paramilitary terrorists. Personality and Individual Differences, 68, 170–175. 10.1016/j.paid.2014.04.031

[cl21111-bib-0011] Allison, K. , & Harris, C. T. (2018). Predicting bias homicide across victim groups: A county‐level analysis. Social Science Research, 74, 108–119. 10.1016/j.ssresearch.2018.05.003 29961478

[cl21111-bib-0012] Altuntas, E. O. (2012). The rise of totalitarian‐police state as a natural phase of capitalism and the U.S. Case. Uluslararasi Iliskiler, 9(35), 35–60.

[cl21111-bib-0013] Amjad, N. , & Wood, A. M. (2009). Identifying and changing the normative beliefs about aggression which lead young Muslim adults to join extremist anti‐Semitic groups in Pakistan. Aggressive Behavior, 35(6), 514–519. 10.1002/ab.20325 19790255

[cl21111-bib-0014] Amnesty International . (2004). Guns & police: Standards to prevent misuse. New York, NY: Author.

[cl21111-bib-0015] Anarumo, M. C. (2005). *What are we really afraid of? The practitioner view of the terrorist threat in the United States* (Doctoral dissertation). Retrieved from ProQuest Dissertations and Theses Global database. (UMI No. 3170744).

[cl21111-bib-0016] Andreopoulos, G. (Ed.). (2013). Policing across borders: Law enforcement networks and the challenges of crime control. New York: Springer Science+Business Media.

[cl21111-bib-0017] Andriyanov, V. N. (2017). Constitutional and legal framework for countering extremism. Russian Journal of Criminology, 11(1), 32–42. 10.17150/2500-4255

[cl21111-bib-0018] Appelboom, P. (2015). *The role of the community officer in the signalling of religious affiliated radicalisation within the Netherlands* (Master's thesis). Retrieved from https://www.scriptiebank.be/sites/default/files/Thesis%20-%20Pieter%20Appelboom.pdf

[cl21111-bib-0019] Argomaniz, J. , & Vidal‐Diez, A. (2015). Examining deterrence and backlash effects in counter‐terrorism: The case of ETA. Terrorism and Political Violence, 27(1), 160–181. 10.1080/09546553.2014.975648

[cl21111-bib-0020] Arnold, L. H. , O'Gwin, C. W. , & Vickers, J. S. (2010). *Small town insurgency: The struggle for information dominance to reduce gang violence* (Master's thesis). Naval Postgraduate School, Monterey, CA.

[cl21111-bib-0021] Asal, V. , Rethemeyer, R. K. , & Young, J. (2015). *Insurgency BAAD: Dynamics of terrorism and counterterrorism campaigns* (Research Brief). College Park, MD: National Consortium for the Study of Terrorism and Responses to Terrorism.

[cl21111-bib-0022] Asongu, S. A. , & Nwachukwu, J. (2019). Mitigating externalities of terrorism on tourism: Global evidence from police, security officers and armed service personnel. Current Issues in Tourism, 22(20), 2466–2471. 10.1080/13683500.2018.1527825

[cl21111-bib-0023] Association of Chief Police Officers, Prevent Delivery Unit . (2009). Channel: A partnership approach to support individuals vulnerable to recruitment by violent extremists. Retrieved from http://www.acpo.police.uk/documents/TAM/Channel.pdf

[cl21111-bib-0024] Australian Federal Police (2005). *AFP economic and social goods 2003‐04 (Research Note No. 8)*. AFP Research Note Series.

[cl21111-bib-0025] Australian Research Council Centre of Excellence in Policing and Security . (2012). COAG review of counter‐terrorism legislation. Canberra, ACT, Australia: Australian Research Council.

[cl21111-bib-0026] Aziz, S. F. (2014). Policing terrorists in the community. Harvard National Security Journal, 5, 147–224.

[cl21111-bib-0027] Babin, S. , Koslicki, W. , Vogel, R. , Contestabile, J. , & Makin, J. (2017). Resilient communications project: Body worn camera perception study phase 1 memorandum report. Laurel, MD: Johns Hopkins Applies Physics Laboratory. Retrieved from https://www.dhs.gov/sites/default/files/publications/971_OIC_AOS-17-1302_Body-Worn-Camera-Perception-Study-Phase-1_171117-508.pdf

[cl21111-bib-0028] Backes, U. (2011). Mobilisations electorales d'extreme droite et violences politiques en Allemagne [Electoral mobilisation of the extreme right wing and political violence in Germany]. Revue des Sciences Sociales, 46, 44–53.

[cl21111-bib-0029] Bailey, A. C. (2009). *An assessment of organizational changes in local Michigan law enforcement agencies in preparation for possible terrorist events* (Doctoral dissertation). Retrieved from ProQuest Dissertations and Theses Global database. (UMI No. 3387329).

[cl21111-bib-0030] Bailey, A. C. , & Cree, L. (2011). Terrorism preparation by Michigan law enforcement agencies. American Journal of Criminal Justice, 36(4), 434–447. 10.1007/s12103-011-9126-2

[cl21111-bib-0031] Bak, T. (2012). Cross‐border cooperation in counteracting the threat of terrorism in the aspect of Euro 2012. Internal Security, 4(2), 101–117.

[cl21111-bib-0032] Baker, A. H. (2011). *Prevent, violent extremists vs. non‐violent extremist and shared values—The debate continues*.

[cl21111-bib-0033] Bank robberies on rise as FBI shifts focus. (2002). *Police*, 26(6), 15.

[cl21111-bib-0034] * Barclay, J. (2011). *Strategy to Reach, Empower, and Educate Teenagers (STREET): A case study in government‐community partnership and direct intervention to counter violent extremism* (Policy Brief). London, UK: Center on Global Counterterrorism Cooperation.

[cl21111-bib-0035] Barros, C. , Caporale, G. , & Gil‐Alana, L. (2009). Basque terrorism: Police action, political measures and the influence of violence on the stock market in the Basque Country. Defence and Peace Economics, 20(4), 287–301.

[cl21111-bib-0036] Bartlett, J. , & Miller, C. (2011). *Should Britain work with ‘extremists’ to prevent terrorism?* Retrieved from https://www.opendemocracy.net/en/opendemocracyuk/should-britain-work-with-extremists-to-prevent-terrorism-where/

[cl21111-bib-0037] Baruch, B. , Ling, T. , Warnes, R. , & Hofman, J. (2018). Evaluation in an emerging field: Developing a measurement framework for the field of counter‐violent‐extremism. Evaluation, 24(4), 475–495. 10.1177/1356389018803218

[cl21111-bib-0038] Bayley, D. H. , & Weisburd, D. (2009). Cops and spooks: The role of the police in counterterrorism. In D. Weisburd , T. Feucht , I. Hakimi , L. Mock & S. Perry (Eds.), To Protect and to serve: Policing in an age of terrorism (pp. 81–99). New York, NY: Springer.

[cl21111-bib-0039] * Beaghley, S. , Helmus, T. C. , Matthews, M. , Ramchand, R. , Stebbins, D. , Kadlec, A. , & Brown, M. A. (2017). Development and pilot test of the RAND Program Evaluation Toolkit for countering violent extremism. Santa Monica, CA: RAND Corporation.

[cl21111-bib-0040] Behlendorf, B. , Belur, J. , & Kumar, S. (2016). Peering through the kaleidoscope: Variation and validity in data collection on terrorist attacks. Studies in Conflict and Terrorism, 39(7‐8), 641–667. 10.1080/1057610X.2016.1141004

[cl21111-bib-0041] Bejan, V. , & Parkin, W. S. (2015). Examining the effect of repressive and conciliatory government actions on terrorism activity in Israel. Economics Letters, 133, 55–58. 10.1016/j.econlet.2015.05.016

[cl21111-bib-0042] Bell, J. (2002). Policing hatred: Law enforcement, civil rights, and hate crime. New York, NY: New York University Press.

[cl21111-bib-0043] * Bellasio, J. , Hofman, J. , Ward, A. , Nederveen, F. , Knack, A. , Meranto, A. S. , & Hoorens, S. (2018). *Counterterrorism evaluation: Taking stock and looking ahead*. Santa Monica, CA: RAND Corporation. Retrieved from https://www.rand.org/content/dam/rand/pubs/research_reports/RR2600/RR2628/RAND_RR2628.pdf

[cl21111-bib-0044] Belli, R. (2012). *Effects and effectiveness of law enforcement intelligence measures to counter homegrown terrorism: A case study on the Fuerzas Armadas de Liberación Nacional (FALN)*. College Park, MD: National Consortium for the Study of Terrorism and Responses to Terrorism.

[cl21111-bib-0045] Bénézech, M. , & Estano, N. (2016). À la recherche d'une âme: Psychopathologie de la radicalisation et du terrorisme [The quest for a soul: Psychology of radicalization and terrorism]. Annales Médico‐psychologiques, Revue Psychiatrique, 174(4), 235–249. 10.1016/j.amp.2016.01.001

[cl21111-bib-0046] Benner, D. N. (2018). *ICE parole and law enforcement programs unit case management systems (DHS/ICE/PIA‐049)*. Washington, DC: U.S. Department of Homeland Security. Retrieved from https://www.dhs.gov/sites/default/files/publications/privacy-pia-ice-plepucms-december2018.pdf

[cl21111-bib-0047] Beutel, A. (2010). Building bridges to strengthen America: Forging an effective counterterrorism enterprise between Muslim Americans and law enforcement. Washington, DC: Muslim Public Affairs Council.

[cl21111-bib-0048] Beutel, A. , & Weinberger, P. (2016). *Public‐private partnerships to counter violent extremism: Field principles for action* (Report to the US Department of State). College Park, MD: National Consortium for the Study of Terrorism and Responses to Terrorism.

[cl21111-bib-0049] Bhulai, R. , & Fink, N. C. (2016). strengthening regional cooperation to prevent and counter violent extremism in South Asia: What role for civil society? Washington DC: Global Center on Cooperative Security.

[cl21111-bib-0050] Bigo, D. , Bonelli, L. , Guittet, E.‐P. , & Ragazzi, F. (2014). Preventing and countering youth radicalisation in the EU. Brussels, Belgium: European Parliament.

[cl21111-bib-0051] Binner, R. , & Junge, M. (2002). 'S etoi publikoi tseremonit'sia ne sleduet' Die Zielgruppen des Befehls Nr. 00447 und der Gro?e Terror aus der Sicht des Befehls Nr. 00447 [The target groups of Order No. 00447 and the Great Terror in the light of Order No. 00447]. Cahiers du Monde Russe, 43(1), 181–228.

[cl21111-bib-0052] Birt, Y. (2009). Promoting virulent envy? Reconsidering the UK's terrorist prevention strategy. The RUSI Journal, 154(4), 52–58. 10.1080/03071840903216460

[cl21111-bib-0053] Biryukov, S. Y. , & Trishina, N. T. (2018). Some aspects of the criminalistic characteristics of crimes committed in the sphere of the military‐industrial complex of Russia in the context of the national security. Pravovaya Paradigma, 17(2), 51–60.

[cl21111-bib-0054] Blackledge, C. (2011). *A motion capture based analysis of the effects of body armor on shooting posture* (Doctoral dissertation). Retrieved from ProQuest Dissertations and Theses Global database. (UMI No. 1502706).

[cl21111-bib-0055] Blakemore, B. , & Awan, I. (2016). Extremism, counter‐terrorism and policing. Abingdon, UK: Routledge.

[cl21111-bib-0056] Boateng, F. D. (2018). Police legitimacy in Africa: A multilevel multinational analysis. Policing & Society, 28(9), 1105–1120. 10.1080/10439463.2017.1280034

[cl21111-bib-0057] Boothman, N. (2016). *How to cooperate with religious organisations and communities within the local approach to radicalisation?* Paper presented at RAN LOCAL, Brussels, Belgium.

[cl21111-bib-0058] Boyd‐Macmillan, E. M. (2016). Increasing cognitive complexity and collaboration across communities: Being Muslim Being Scottish. Journal of Strategic Security, 9(4), 79–110. 10.5038/1944-0472.9.4.1563

[cl21111-bib-0059] Bradford, T. , Pietra, P. , & Callahan, M. E. (2009). *Privacy impact assessment for e‐law enforcement officer logbook program (e‐logbook)*. Washington, DC: U.S. Department of Homeland Security. Retrieved from https://www.dhs.gov/sites/default/files/publications/privacy_pia_tsa_elogbook_0.pdf

[cl21111-bib-0060] Braga, A. A. , & Weisburd, D. (2006). Conclusion: Police innovation and the future of policing. In D. Weisburd & A. A. Braga (Eds.), Police innovation: Contrasting perspectives (pp. 399–352). Cambridge, UK: Cambridge University Press.

[cl21111-bib-0061] Bransford, S. D. (2012). *An examination of factors affecting information sharing among law enforcement agencies* (Doctoral dissertation). Retrieved from ProQuest Dissertations and Theses Global database. (UMI No. 3514665).

[cl21111-bib-0062] Braziel, R. , Straub, F. , Watson, G. , & Hoops, R. (2015). *Bringing calm to chaos: A critical incident review of the San Bernardino public safety response to the December 2, 2015 terrorist shooting incident at the Inland Regional Center*. Washington, DC: Community Oriented Policing Services, U.S. Department of Justice.

[cl21111-bib-0063] * Brett, J. (2012). *Recent Danish counterradicalization initiatives: A case study on the Danish Security and Intelligence Service's dialogue forum (Policy Brief)*. London, UK: Center on Global Counterterrorism Cooperation.

[cl21111-bib-0064] Brewer, N. , Weber, N. , Wootton, D. , & Lindsay, D. S. (2012). Identifying the bad guy in a lineup using confidence judgments under deadline pressure. Psychological Science, 23(10), 1208–1214. 10.1177/0956797612441217 22933457

[cl21111-bib-0065] Briggs, R. (2010). Community engagement for counterterrorism: Lessons from the United Kingdom. International Affairs, 86(4), 971–981. 10.1111/j.1468-2346.2010.00923.x

[cl21111-bib-0066] Briggs, R. , Fieschi, C. , & Lownsbrough, H. (2006). Bringing it home: Community‐based approaches to counter‐terrorism. London, UK: Demos.

[cl21111-bib-0067] Brinser, K. L. , & King, W. R. (2016). Organizational permeability to environmental conditions: Local police agency assessments of threats posed by disasters, accidents, and terrorism. Police Quarterly, 19(4), 387–409. 10.1177/1098611115626409

[cl21111-bib-0068] Broadbent, R. (2013). Using grass roots community programs as an anti‐extremism strategy. Australian Journal of Adult Learning, 53(2), 187–210.

[cl21111-bib-0069] Brooks, A. (2015). *Policing and the likelihood of terrorism: A community structural approach to an uncertain relationship* (Doctoral dissertation). Retrieved from http://scholarworks.uark.edu/cgi/viewcontent.cgi?article=2095&context=etd

[cl21111-bib-0070] Brown, B. (2007). Community policing in post‐September 11 America: A comment on the concept of community‐oriented counterterrorism. Police Practice and Research, 8(3), 239–251. 10.1080/15614260701450716

[cl21111-bib-0071] Brown, I. , & Cowls, J. (2015). Check the web: Assessing the ethics and politics of policing the internet for extremist material. Dublin, Ireland: VOX‐Pol Network of Excellence.

[cl21111-bib-0072] Buesa, M. , & Baumert, T. (2016). Hit the core or weaken the periphery? Comparing strategies to break the circle of violence with an embryonic terrorist group: The case of Galician Resistance. Terrorism and Political Violence, 30(3), 475–502. 10.1080/09546553.2016.1182

[cl21111-bib-0073] Bug, M. (2016). Terrorismusbekampfung als waffe gegen alltagskriminalitat — argumentation und wirklichkeit der vorratsdatenspeicherung in Deutschland [Counter‐terrorism as a toll against small‐scale crime — reason and reality of retaining communication data in Germany]. Zeitschrift fur Parlamentsfragen, 47(3), 670–692.

[cl21111-bib-0074] Bull, R. , Valentine, T. & Williamson, T. (Eds.). (2009). Handbook of psychology of investigative interviewing: Current developments and future directions. Chichester, UK: Wiley‐Blackwell.

[cl21111-bib-0075] Bunker, R. J. (2005). Networks, terrorism and global insurgency. London, UK: Routledge.

[cl21111-bib-0076] Bureau of Justice Association (2013). *National Criminal Intelligence Sharing Plan. Building a national capability for effective criminal intelligence development and the nationwide sharing of intelligence and information: The next step in the evolution of the National Criminal Intelligence Sharing Plan*. Washington, DC: Bureau of Justice Association.

[cl21111-bib-0077] Burruss, G. W. , Schafer, J. A. , Giblin, M. J. , & Haynes, M. R. (2012). *Homeland security in small law enforcement jurisdictions: Preparedness, efficacy, and proximity to big‐city peers* (Document No. 252137). Retrieved from https://www.ncjrs.gov/pdffiles1/nij/grants/239466.pdf

[cl21111-bib-0078] Butkevich, S. (2013). Concept of combating terrorism in Ukraine. Organizational and legal aspects. Internal Security, 5(2), 141–152.

[cl21111-bib-0079] Butt, R. , & Tuck, H. (2014). *European counter‐radicalisation and de‐radicalisation: A comparative evaluation of approaches in the Netherlands. Sweden, Denmark and Germany*. London, UK: Institute for Strategic Dialogue.

[cl21111-bib-0080] CACP Prevention of Radicalization Study Group (2008). *Building community resilience to violent ideologies*. Ottawa, ON, Canada: Canadian Association of Chiefs of Police.

[cl21111-bib-0081] Caleta, D. (2009). Stalisca pripadnikov slovenske vojske do mednarodnega terorizma [The standpoints of members of the armed forces regarding international terrorism]. Teorija in Praksa, 46(1‐2), 126–145.

[cl21111-bib-0082] Campbell, J. (2016). *Federal and state cooperation: The role of state law enforcement in the enforcement of illegal immigration following 9/11* (Doctoral dissertation). Retrieved from ProQuest Dissertations and Theses Global database. (UMI No. 10111465).

[cl21111-bib-0083] Canfield, P. (2016). An evaluation of the police response to gang‐related violence and future security threats. In A. J. Masys (Ed.), Exploring the Security Landscape: Non‐Traditional Security Challenges (pp. 101–139). Cham, Switzerland: Springer.

[cl21111-bib-0084] Carlile, A. (2011). *Report to the Home Secretary of independent oversight of prevent review and strategy*. Retrieved from https://assets.publishing.service.gov.uk/government/uploads/system/uploads/attachment_data/file/97977/lord-carlile-report.pdf

[cl21111-bib-0085] Carter, J. G. (2013). Intelligence‐led policing: A policing innovation. El Paso, TX: LFB Scholarly Publishing LLC.

[cl21111-bib-0086] Carter, J. G. (2015). Intelligence analysis within U.S. law enforcement agencies: Empirical insights from a national sample. Journal of Intelligence Analysis, 22(3), 1–24.

[cl21111-bib-0087] Carter, J. G. , Carter, D. L. , & Chermak, S. (2013). Intelligence training. Law Enforcement Executive Forum, 13(2), 1–18.

[cl21111-bib-0088] Carter, J. G. , & Phillips, S. W. (2015). Intelligence‐led policing and forces of organisational change in the USA. Policing & Society, 25(4), 333–357. 10.1080/10439463.2013.865738

[cl21111-bib-0089] Carter, J. G. , Phillips, S. W. , & Gayadeen, S. M. (2014). Implementing intelligence‐led policing: An application of loose‐coupling theory. Journal of criminal justice, 42, 433–442. 10.1016/j.jcrimjus.2014.08.002

[cl21111-bib-0090] Center on Global Counter‐Terrorism Cooperation (2007). Implementing the United Nations General Assembly's Global Counter‐Terrorism Strategy in the Asia‐Pacific. New York, NY: Center on Global Counter‐Terrorism Cooperation.

[cl21111-bib-0091] Center on Global Counterterrorism Cooperation (2012). Fighting terror through justice: Implementing the IGAD framework for legal cooperation against terrorism . IGAP Security Sector Program, Retrieved from https://www.globalcenter.org/wp-content/uploads/2012/06/TaskForce_Report_May20121.pdf

[cl21111-bib-0092] Center on Global Counterterrorism Cooperation . (2013). *To protect and prevent: Outcomes of a global dialogue to counter terrorist abuse of the nonprofit sector*. Retrieved from https://www.globalcenter.org/wp-content/uploads/2013/06/CGCC_Prevent-Protect-Report_pgs.pdf

[cl21111-bib-0093] Centre International Pour la Prévention de la Criminalité (CIPC) (2015). Comment prévenir la radicalisation: Une revue systématique. Montréal, QC, Canada: CIPC.

[cl21111-bib-0094] Chalk, P. , & Rosenau, W. (2004). *Confronting the "enemy within": Security intelligence, the police, and counterterrorism in four democracies*. Santa Monica, CA: RAND Corporation.

[cl21111-bib-0095] Challgren, J. , Kenyon, T. , Kervick, L. , Scudder, S. , Walters, M. , Whitehead, K. , & Flynn, C. R. (2016). *Countering violent extremism: Applying the public health model*. Washington, DC: Georgetown University Center for Security Studies.

[cl21111-bib-0096] Chandra, A. , Acosta, J. , Stern, S. , Uscher‐Pines, L. , Williams, M. V. , Yeung, D. , & Meredith, L. S. (2011). *Building community resilience to disasters: A way forward to enhance national health security* (Technical Report). Santa Monica, CA: RAND Corporation. 10.7249/rhq-01-01-06 PMC4945213 28083162

[cl21111-bib-0097] Chappell, A. T. , & Gibson, S. A. (2009). Community policing and homeland security policing: Friend or foe? Criminal Justice Policy Review, 20(3), 326–343. 10.1177/0887403409333038

[cl21111-bib-0098] Chappell, G. , & Callahan, M. E. (2009). *Privacy impact assessment for the marine information for safety and law enforcement (MISLE) system*. Washington; DC: U.S. Department of Homeland Security. Retrieved from https://www.dhs.gov/sites/default/files/publications/privacy_pia_008_uscg_misle_2009.pdf

[cl21111-bib-0099] Chermak, S. M. , Freilich, J. D. , & Caspi, D. (2009). Policymakers and law enforcement must consider the unintended consequences of their proposed responses to extremist and terrorist groups. In N. A. Frost , J. D. Freillich & T. R. Clear (Eds.), Contemporary issues in criminal justice policy: Policy proposals from the American Society of Criminology Conference (pp. 151–158). Belmont, CA: Wadsworth, CENGAGE Learning.

[cl21111-bib-0100] Chermak, S. M. , Freilich, J. D. , & Shemtob, Z. (2009). Law enforcement training and the domestic far right. Criminal Justice and Behavior, 36, 1305–1322. 10.1177/0093854809345630

[cl21111-bib-0101] Chermak, S. M. , Freilich, J. D. , & Simone, J. Jr. (2010). Surveying American state police agencies about lone wolves, far‐right criminality, and far‐right and Islamic jihadist criminal collaboration. Studies in Conflict & Terrorism, 33(11), 1019–1041. 10.1080/1057610X.2010.514698

[cl21111-bib-0102] Cherney, A. , & Murphy, K. (2013). Policing terrorism with procedural justice: The role of police legitimacy and law legitimacy. Australian and New Zealand Journal of Criminology, 46(3), 403–421. 10.1177/0004865813485072

[cl21111-bib-0103] * Cherney, A. , & Murphy, K. (2017). Police and community cooperation in counterterrorism: Evidence and insights from Australia. Studies in Conflict and Terrorism, 40(12), 1023–1037. 10.1080/1057610X.2016.1253987

[cl21111-bib-0104] Cherney, A. , & Murphy, K. (2019). Support for terrorism: The role of beliefs in Jihad and institutional responses to terrorism. Terrorism and Political Violence, 31(5), 1049–1069. 10.1080/09546553.2017.1313735

[cl21111-bib-0105] Cherney, A. , Sweid, R. , Grossman, M. , Derbas, A. , Dunn, K. , Jones, C. , & Barton, G. (2018). Local service provision to counter violent extremism: perspectives, capabilities and challenges arising from an Australian service mapping project. Behavioral Sciences of Terrorism and Political Aggression, 10(3), 187–206. 10.1080/19434472.2017.1350735

[cl21111-bib-0106] Chestnut, S. (2007). Illicit activity and proliferation—North Korean smuggling networks. International Security, 32(1), 80–111. 10.1162/isec.2007.32.1.80

[cl21111-bib-0107] Chong, A. , & Lopez‐De‐Silanes, F. (2015). Money laundering and its regulation. Economics & Politics, 27(1), 78–123. 10.1111/ecpo.12051

[cl21111-bib-0108] Chongruksa, D. , Pansomboon, C. , Prinyapol, P. , & Sawatsri, S. (2011). Efficacy of eclectic group counseling in addressing stress among Thailand police officers in terrorist situations. European Journal of Psychotraumatology, 2(1), 79.

[cl21111-bib-0109] Chongruksa, D. , Parinyapol, P. , Sawatsri, S. , & Pansomboon, C. (2012). Efficacy of eclectic group counseling in addressing stress among Thai police officers in terrorist situations. Counselling Psychology Quarterly, 25(1), 83–96. 10.1080/09515070.201

[cl21111-bib-0110] Choudhury, T. (2009). *Stepping out: Supporting exit strategies from violence and extremism*. London, UK: Institute for Strategic Dialogue.

[cl21111-bib-0111] Choudhury, T. , & Fenwick, H. (2011). The impact of counter‐terrorism measures on Muslim communities. International Review of Law. Computers & Technology, 25(3), 151–181. 10.1080/13600869.2011.617491

[cl21111-bib-0112] Chowdhury Fink, N. , Romaniuk, P. , Millar, A. , & Ipe, J. (2014). *Blue sky II: Progress and opportunities in implementing the UN Global Counter‐Terrorism Strategy*. Goshen, IN: Global Center on Cooperative Security.

[cl21111-bib-0113] Chrismas, R. (2013). Canadian policing in the 21st century: A frontline officer on challenges and changes. Montreal, QC, Canada: McGill‐Queen's University Press.

[cl21111-bib-0114] Christensen, D. A. , & Aars, J. (2017). Nordmenns holdninger til telefonavlytting: Resultater fra et surveyeksperiment [Norwegian attitudes to wiretapping: Results from a survey experiment]. Tidsskrift for Samfunnsforskning, 58(2), 191–209.

[cl21111-bib-0115] Christiansen, P. , Alison, L. , & Alison, E. (2018). Well begun is half done: Interpersonal behaviours in distinct field interrogations with high‐value detainees. Legal and Criminological Psychology, 23(1), 68–84. 10.1111/lcrp.12111

[cl21111-bib-0116] Christmann, K. (2012). *Preventing religious radicalisation and violent extremism: A systematic review of the research evidence*. London, UK: Youth Justice Board.

[cl21111-bib-0117] Ciabuca, A. E. (2014). Evaluarea imaginii publice a poliţiei prin intermediul Diferenţiatorului Semantic, între real şi deziderat [The evaluation of the perceived and the projected public image of the police using the Semantic Differentiator scale.]. Psihologia Resurselor Umane, 12, 159–173.

[cl21111-bib-0118] Ciftci, I. (2013). *The role of soft‐line governmental policy interventions towards terrorist organizations during democratization period: A comparative case study between the PKK and ETA* (Doctoral dissertation). Newark, NJ, Rutgers University.

[cl21111-bib-0119] Clare, J. , Brantingham, P. J. , & Brantingham, P. L. (2012). Approches de maintien de l'ordre axé sur la résolution des problèmes en ce qui concerne la culture extérieure du cannabis. Ottawa, ON, Canada: Sécurité Publique Canada.

[cl21111-bib-0120] Clarke, P. (2007). Learning from experience: Counter‐terrorism in the UK since 9/11. London, UK: Policy Exchange.

[cl21111-bib-0121] Claybo, D. B. (2011). *Engagement, partnership, or security? Clarifying the role of community policing in Afghanistan's counterinsurgency* (Master's thesis). Retrieved from http://hdl.handle.net/2429/38530

[cl21111-bib-0122] Cockayne, J. , Millar, A. , Cortright, D. , & Romaniuk, P. (2012). *Reshaping United Nations counterterrorism efforts: Blue‐sky thinking for global counterterrorism cooperation 10 years after 9/11*. New York, NY: Center on Global Counterterrorism Cooperation.

[cl21111-bib-0123] Coles, J. B. , & Zhuang, J. (2014). Decisions in disaster recovery operations: A game theoretic perspective on organization cooperation. In A. B. Badiru & L. Racz (Eds.), Handbook of emergency response (pp. 465–480). Boca Raton, FL: CRC Press.

[cl21111-bib-0124] Comens, C. L. (2016). *Terrorism preparedness: A law enforcement perspective* (Doctoral dissertation). Retrieved from ProQuest Dissertations and Theses Global database. (UMI No. 10129393).

[cl21111-bib-0125] *Commission of Inquiry into the Investigation of the Bombing of Air India Flight 182*. (2011). Air India Inquiry Action Plan, Progress report. Retrieved from http://www.publicsafety.gc.ca/lbrr/archives/cn27667-eng.pdf

[cl21111-bib-0126] Correia, P. M. A. R. , & de Jesus, I. O. A. (2016). Combate às transferências bancárias ilegítimas pela Internet no direito português: Entre as experiências domésticas e políticas globais concertadas [Combating illegitimate bank transfers through the internet in Portuguese law: Between domestic experiences and concerted global policies]. Revista Direcito Gv, 12(2), 542–563.

[cl21111-bib-0127] Cortright, D. & Lopez, G. A. (Eds.). (2007). Uniting against terror: Cooperative nonmilitary responses to the global terrorist threat. Cambridge, MA: MIT Press.

[cl21111-bib-0128] Counter‐Terrorism & National Security Committee (2010). Annual report. Ottawa, ON, Canada: Canadian Association of Chiefs of Police.

[cl21111-bib-0129] *Counterterrorism: Investigator task list*. (2013). Ottawa, ON, Canada: Police Sector Council.

[cl21111-bib-0130] Coyne, J. , & Bell, P. (2015). The role of strategic intelligence in law enforcement: Policing transnational organized crime in Canada, the United Kingdom and Australia. Basingstoke, UK: Palgrave, Macmillan.

[cl21111-bib-0131] Crenshaw, M. (Ed.). (2010). The consequences of counterterrorism. New York, NY: Russell Sage Foundation.

[cl21111-bib-0132] Crutcher, N. , & Budak, M. (2005). *The Anti‐Terrorism Act and security measures in Canada: Public views, impacts and travel experiences* (Report no. rr05‐11e). Ottawa, ON, Canada: Research and Statistics Division, Department of Justice Canada.

[cl21111-bib-0133] Czeszejko‐Sochacka, K. (2013). The offence of taking a hostage as an offence against public order. Panstwo i Prawo, 8, 67–79.

[cl21111-bib-0134] Dahl, E. J. (2014). Local approaches to counterterrorism: The New York Police Department model. Journal of Policing, Intelligence and Counter Terrorism, 9(2), 81–97. 10.1080/18335330.2014.940815

[cl21111-bib-0135] Dandurand, Y. (2005). *Strategies and practical measures to strengthen the capacity of prosecution services in dealing with transnational organized crime, terrorism and corruption*. Working paper presented at the 2nd World Summit of Attorneys General, Prosecutors General and Chief Prosecutors, Doha, Qatar.

[cl21111-bib-0136] Dandurand, Y. (2009). Handbook on criminal justice responses to terrorism. Vienna, Austria: United Nations Office on Drugs and Crime.

[cl21111-bib-0137] Dandurand, Y. , & Chin, V. (2004). *Links between terrorism and other forms of crime*. Vancouver, BC, Canada: International Centre for Criminal Law Reform and Criminal Justice Policy.

[cl21111-bib-0138] Danish Ministry of Refugee Immigration and Integration Affairs . (2010). The challenge of extremism: Examples of deradicalisation and disengagement programmes in the EU. Copenhagen, Denmark: Danish Government.

[cl21111-bib-0139] Daun, A. (2005). Intelligence—Strukturen fur die multilaterale Kooperation europaischer Staaten. Integration, 28(2), 136–149.

[cl21111-bib-0140] David, L. M. , Riley, K. J. , Ridgeway, G. , Pace, J. , Cotton, S. K. , Steinberg, P. S. , … Smith, B. L. (2004). *When terrorism hits home: How prepared are state and local law enforcement?* Santa Monica, CA: RAND Corporation.

[cl21111-bib-0141] Davies, W. A. (2018). Counterterrorism effectiveness to Jihadists in Western Europe and the United States: We are losing the war on terror. Studies in Conflict and Terrorism, 41(4), 281–296. 10.1080/1057610X.2017.1284447

[cl21111-bib-0142] Davies, M. , Warnes, R. , & Hofman, J. (2017). *Exploring the transferability and applicability of gang evaluation methodologies to counter‐violent radicalisation*. Santa Monica, CA: RAND Corporation.

[cl21111-bib-0144] Davis, P. K. (2012). Toward an analytic basis for influence strategy in counterterrorism. In A. Wenger & A. Wilner (Eds.), Deterring Terrorism: Theory and Practice (pp. 67–94). Stanford, CA: Stanford University Press.

[cl21111-bib-0145] Davis, L. M. , Helmus, T. C. , Hunt, P. , Payne, L. A. , Jahedi, S. , & Tsang, F. (2016). *Assessment of the State and Local Anti‐Terrorism Training (SLATT) Program*. Santa Monica, CA: RAND Corporation.

[cl21111-bib-0146] Davis, P. K. , Oerry, W. L. , Manheim, D. , & Hollywood, J. (2015). *Using causal models in heterogeneous information fusion to detect terrorists*. Paper presented at the 2015 Winter Simulation Conference, Huntington Beach, CA.

[cl21111-bib-0147] Davis, L. M. , Pollard, M. , Ward, K. , Wilson, J. M. , Varda, D. M. , Hansell, L. , & Steinberg, P. (2010). *Long‐term effects of law enforcement's post‐9/11 focus on counterterrorism and homeland security*. Santa Monica, CA: RAND Corporation.

[cl21111-bib-0148] Davis, L. M. , Riley, J. , Ridgeway, G. , Pace, J. , Cotton, S. K. , Steinberg, P. S. , … Smith, B. L. (2004). *When terrorism hits home: How prepared are state & local law enforcement*. Santa Monica, CA: RAND Corporation.

[cl21111-bib-0149] Dawson, E. , Hartwig, M. , Brimbal, L. , & Denisenkov, P. (2017). A room with a view: Setting influences information disclosure in investigative interviews. Law and Human Behavior, 41(4), 333–343. 10.1037/lhb0000244 28459264

[cl21111-bib-0150] Delice, M. , & Daglar, M. (2011). *Alternative tactics for combating terrorism: Citizens police academy* (Working Paper No. 29). Geneva, Switzerland: Geneva Centre for the Democratic Control of Armed Forces, International Police Executive Symposium.

[cl21111-bib-0151] * Deloughery, K. , & Owens, R. (2016). *Countering violent extremism—Developing a research roadmap: Literature review*. RTI International. Retrieved from https://www.dhs.gov/sites/default/files/publications/OPSR_TP_CVE-Developing-Research-Roadmap_Literature-Review_180411-508.pdf

[cl21111-bib-0152] Demant, F. , & de Graaf, B. (2010). How to counter radical narratives: Dutch deradicalization policy in the case of Moluccan and Islamic radicals. Studies in Conflict & Terrorism, 33(5), 408–428. 10.1080/10576101003691549

[cl21111-bib-0153] Demme, N. (2007). *Government expectations and the role of law enforcement in a biological incident* (Master's thesis). Retrieved from https://apps.dtic.mil/dtic/tr/fulltext/u2/a467116.pdf

[cl21111-bib-0154] Denemark, D. (2012). Trust, efficacy and opposition to anti‐terrorism police power: Australia in comparative perspective. Australian Journal of Political Science, 47(1), 91–113. 10.1080/10361146.2011.643163

[cl21111-bib-0155] Desta, T. A. (2013). *The anti‐money laundering and countering terrorist financing regime in Ethiopia* (Working Paper). Goshen, IN: Center on Global Counterterrorism Cooperation

[cl21111-bib-0156] Di Tella, R. , & Schargrodsky, E. (2004). Do police reduce crime? Estimates using allocations of police forces after a terrorist attack. American Economic Review, 94(1), 115–133.

[cl21111-bib-0157] Dinkis, J. , & Callahan, M. E. (2011). *Privacy compliance review of the ICE pattern analysis and information collection law enforcement intelligence sharing service*. Washington, DC: U.S. Department of Homeland Security. Retrieved from https://www.dhs.gov/sites/default/files/publications/privacy_privcomrev_ice-analysis.pdf

[cl21111-bib-0158] Dion‐Schwartz, C. , Ryan, N. , Thompason, J. A. , Silfersten, E. , & Paoli, G. P. (2018). *Olympic‐caliber cybersecurity: Lessons for safeguarding the 2020 Games and other major events*. Santa Monica, CA: RAND Corporation.

[cl21111-bib-0159] Disley, E. , Pardal, M. , Weed, K. , & Reding, A. (2016). *Using multi agency public protection arrangements to manage and supervise terrorist offenders: Findings from an exploratory study*. Santa Monica, CA: RAND Corporation.

[cl21111-bib-0160] Donner, C. M. , Maskaly, J. , Piquero, A. R. , & Jennings, W. G. (2017). Quick on the draw: Assessing the relationship between low self‐control and officer‐involved police shootings. Police Quarterly, 20(2), 213–234. 10.1177/1098611116688066

[cl21111-bib-0161] Draca, M. , Machin, S. J. , & Witt, R. (2008a). *Panic on the streets of London: Police, crime and the July 2005 terror attacks* (CEP Discussion Paper No. 852). London, UK: Centre for Economic Performance.

[cl21111-bib-0162] Draca, M. , Machin, S. , & Witt, R. (2008b). *Panic on the streets of London: Police, crime and the July 2005 terror attacks* (IZA Discussion Paper No. 3410). IZA: Bonn, Germany.

[cl21111-bib-0163] Dremliuga, R. I. , Korobeev, A. I. , & Fedorov, A. V. (2017). Cyberterrorism in China: Criminal law and criminological aspects. Russian Journal of Criminology, 11(3), 607–614. 10.17150/2500-4255

[cl21111-bib-0164] Dubber, M. D. (2009). *The War on Terror and U.S. criminal law*. Rochester, NY: Social Science Research Network.

[cl21111-bib-0165] DuBois, D. L. , & Alem, F. (2017). *Mentoring and domestic radicalization*. Chicago, IL: National Mentoring Resource Center.

[cl21111-bib-0166] Dugan, L. , Lafree, G. , & Piquero, A. R. (2005). Testing a rational choice model of airline hijackings. Criminology, 43(4), 1031–1065.

[cl21111-bib-0167] Durakovic, A. (2014). Zasjeda kao operativno‐taktička radnja i kao taktika kriminalaca [Ambush as police and criminal's tactics]. Polemos, 17(33‐34), 11–27.

[cl21111-bib-0168] Earl, J. , & Soule, S. A. (2010). The impacts of repression: The effect of police presence and action on subsequent protest rates. Research in Social Movements, 30, 75–113. 10.1108/S0163-786X(2010)0000030006

[cl21111-bib-0169] Earl, J. , Soule, S. A. , & McCarthy, J. D. (2003). Protest under fire? Explaining the policing of protest. American Sociological Review, 69(4), 581–606. 10.2307/1519740

[cl21111-bib-0170] Economic and Social Council (2012). *Assistance in implementing the international conventions and protocols related to terrorism*. Report of the Secretary‐General. Vienna, Austria: United Nations Office on Drugs and Crime.

[cl21111-bib-0171] Economic and Social Council (2014). *Assistance in implementing the international conventions and protocols related to terrorism: Report of the Secretary‐General*. Vienna, Austria: United Nations Office on Drugs and Crime.

[cl21111-bib-0172] Eijkman, Q. , & Roodnat, J. (2017). Beware of branding someone a terrorist: Local professionals on person‐specific interventions to counter extremism. Journal for Deradicalization, 10, 175–202.

[cl21111-bib-0173] El Sayed, L. & Barnes, J. (Eds.). (2017). Contemporary P/CVE research and practice. New York, NY: Hedayah.

[cl21111-bib-0174] Ellis, J. (2014). Fundamentals of homeland security: An operations perspective. Springfield, IL: Charles C Thomas.

[cl21111-bib-0175] Ellis, J. W. (2007). Police analysis and planning for homicide bombings: Prevention, defense, and response. Springfield, IL: Charles C Thomas Publishing.

[cl21111-bib-0176] * Ellis, A. , Cleary, A. , Innes, M. , & Zeuthen, M. (2011). *Monitoring and evaluation tools for counterterrorism program effectiveness* (Policy Brief). London, UK: Center on Global Counterterrorism Cooperation.

[cl21111-bib-0177] El‐Said, H. (2012). *De‐radicalising Islamists: Programmes and their impact in Muslim majority states*. London, UK: The International Centre for the Study of Radicalisation and Political Violence.

[cl21111-bib-0178] El‐Said, H. (2015). New approaches to countering terrorism: Designing and evaluating counter radicalization and de‐radicalization programs. London, UK: Palgrave Macmillan.

[cl21111-bib-0179] Eterno, J. (2003). Policing within the law: A case study of the New York City Police Department. London, UK: Praeger.

[cl21111-bib-0180] *Executive Office of the President of the United States . (2016). Strategic implementation plan for empowering local partners to prevent violent extremism in the United States. Retrieved from https://www.dhs.gov/sites/default/files/publications/2016_strategic_implementation_plan_empowering_local_partners_prev.pdf

[cl21111-bib-0181] Falciola, L. (2013). Gli apparati di polizia di fronte al movimento del 1977: Organizzazione e dinamiche interne [Italian police and the 1977 movement: Organization and internal dynamics]. Ricerche di Storia Politica, 2, 161–182.

[cl21111-bib-0182] Fantrov, P. P. , & Shinkaruk, V. M. (2018). The regional strategy of the national security of the south of Russia: Confrontation of civil associations with criminal threats. Pravovaya Paradigma, 17(2), 33–40. 10.15688/lc.jvolsu

[cl21111-bib-0183] Feddes, A. R. , & Gallucci, M. (2015). A literature review on methodology used in evaluating effects of preventive and de‐radicalisation interventions. Journal for Deradicalization, 5, 1–27.

[cl21111-bib-0184] Feng, J. , Zhou, J. , & Jain, A. K. (2013). Orientation field estimation for latent fingerprint enhancement. IEEE Transactions on Pattern Analysis and Machine Intelligence, 35(4), 925–940. 10.1109/TPAMI.2012.155 22826508

[cl21111-bib-0185] Fernández, A. M. D. (2010). La construcción de una capacidad de inteligencia durante las presidencias españolas de la Unión Europea. Revista CIDOB d'Afers Internacionals, 91, 147–171. 10.2307/25822750

[cl21111-bib-0186] Fernandez, L. , & Merzer, M. (2003). Jane's crisis communications handbook. Alexandria, VA: Jane's Information Group.

[cl21111-bib-0187] Ferzan, I. H. (2009). *The perceptions of Turkish police captains about the use of techniques of neutralization (neutralization theory) by Turkish offenders* (Doctoral dissertation). Retrieved from ProQuest Dissertations and Theses database. (UMI No. 3360050).

[cl21111-bib-0188] Feve, S. , & Elshimi, M. (2018). *Planning for prevention: A framework to develop and evaluate national action plans to prevent and counter violent extremism*. London, UK: Center on Global Counterterrorism Cooperation.

[cl21111-bib-0189] Fink, N. C. , Romaniuk, P. , & Barakat, R. (2013). *Evaluating countering violent extremism programming: Practice and progress*. Goshen, IN: Center on Global Counterterrorism Cooperation.

[cl21111-bib-0190] Fischer, M. G. (2004). Terrorismusbekämpfung durch die Bundeswehr im Inneren Deutschlands? JuristenZeitung, 59(8), 376–384. 10.2307/20827260

[cl21111-bib-0191] Fishering, S. (2018). *Improvements, updates, and support for the TEVUS portal: Report to the U.S. Department of Homeland Security Science and Technology Directorate*. National Consortium for the Study of Terrorism and Responses to Terrorism, Retrieved from https://www.dhs.gov/sites/default/files/publications/984_OPSR_TP_TEVUS_Improvements-Updates-Support-TEVUS-Portal_2018June-508.pdf

[cl21111-bib-0192] Fleming, J. , & Wood, J. (2006). Fighting crime together: The challenges of policing and security networks. Sydney, NSW, Australia: University of New South Wales Press.

[cl21111-bib-0193] Florio, K. E. (2014). *National origin disparities in the New York city police department's stop‐and‐frisk practices since 9/11* (Doctoral dissertation). Retrieved from ProQuest Dissertations and Theses Global database. (UMI No. 1554555).

[cl21111-bib-0194] Foley, F. (2009). Reforming counterterrorism: Institutions and organizational routines in Britain and France. Security Studies, 18(3), 435–478. 10.1080/09636410903132920

[cl21111-bib-0195] Foley, F. (2013). Countering terrorism in Britain and France: Institutions, norms, and the shadow of the past. Cambridge, NY: Cambridge University Press.

[cl21111-bib-0196] Freilich, J. D. , & Chermak, S. M. (2009). Preventing deadly encounters between law enforcement and American far‐rightists. In J. D. Freilich & G. R. Newman (Eds.), Reducing terrorism through situational crime prevention (pp. 141–172). New York, NY: Criminal Justice Press.

[cl21111-bib-0197] Freilich, J. D. , Chermak, S. M. , & Simone, J., Jr. (2009). Surveying American state police agencies about terrorism threats, terrorism sources, and terrorism definitions. Terrorism and Political Violence, 21(3), 450–475. 10.1080/09546550902950324

[cl21111-bib-0198] Frenett, R. , & Dow, M. (2014). *One to one interventions: A pilot CVE methodology*. London, UK: Institute for Strategic Dialogue and Curtain University.

[cl21111-bib-0199] Friedrichs, J. (2007). *Fighting terrorism and drugs: Europe and international police cooperation*. Abingdon, UK: Routledge.

[cl21111-bib-0200] Friemert, B. , Franke, A. , Bieler, D. , Achatz, A. , Hinck, D. , & Engelhardt, M. (2017). Treatment strategies for mass casualty incidents and terrorist attacks in trauma and vascular surgery: Presentation of a treatment concept. Der Chirurg, 88, 856–862. 10.1007/s00104-017-0490-4 28801785

[cl21111-bib-0201] Fröhlich, P. D. S. (2011). Deutschlands rolle in der EU und NATO beim konfliktmanagement in Afghanistan [Germany's conflict management in Afghanistan within the scope of the EU and NATO]. Zeitschrift für Außen‐und Sicherheitspolitik, 4(1), 31–43. 10.1007/s12399-011-0205-8

[cl21111-bib-0202] Garcia‐Retamero, R. , & Dhami, M. K. (2013). On avoiding framing effects in experienced decision makers. The Quarterly Journal of Experimental Psychology, 66(4), 829–842. 10.1080/17470218.2012.727836 23098268

[cl21111-bib-0203] Geller, W. , & Stephens, D. (2003). Local government police management. Washington, DC: ICMA Press.

[cl21111-bib-0204] Gendarmerie royale du Canada (2007). *Les mesures prises par la GRC suite aux recommandations découlant du rapport sur les événements concernant Maher Arar* (la Commission O'Connor). West Ottawa, ON, Canada: Public Safety Canada Library.

[cl21111-bib-0205] Gendarmerie Royale du Canada (2009). *Démystifier la radicalisation/Enquêtes criminelles relatives à la sécurité nationale*. Ottawa, ON, Canada: Gendarmerie Royale du Canada.

[cl21111-bib-0206] Gerber, B. J. , Cohen, D. B. , Cannon, B. , Patterson, D. , & Stewart, K. (2005). On the front line: American cities and the challenge of homeland security preparedness. Urban Affairs Review, 41(2), 182–210. 10.1177/1078087405279900

[cl21111-bib-0207] Ghosh, P. , Warfa, N. , McGilloway, A. , Ali, I. , Jones, E. , & Bhui, K. (2013). Violent radicalisation and recruitment to terrorism: Perspectives of wellbeing and social cohesion of citizens of Muslim heritage. Sociology Mind, 3(4), 290–297. 10.4236/sm.2013.34039

[cl21111-bib-0208] Giannone, D. , & Robert, A. (2003). Enlisting community members in the fight against terrorism. The Police Chief, 70(3), 37–38.

[cl21111-bib-0209] Gibbs, J. C. (2012). *The relationship between legitimacy, terrorist attacks and police* (Doctoral dissertation). Retrieved from ProQuest Dissertations and Theses Global database. (UMI No. 3495332).

[cl21111-bib-0210] Gibbs, J. C. (2018). Terrorist attacks targeting the police: The connection to foreign military presence. Police Practice & Research, 19(3), 222–240. 10.1080/15614263.2017.1295245

[cl21111-bib-0211] Giblin, M. J. , Schafer, J. A. , & Burruss, G. W. (2009). Homeland security in the heartland: Risk, preparedness, and organizational capacity. Criminal Justice Policy Review, 20(3), 274–289. 10.1177/0887403408323762

[cl21111-bib-0212] Gill, P. (2012). Terrorist violence and the contextual, facilitative and casual qualities of group‐based behaviors. Aggression and Violent Behavior, 17, 565–574. 10.1016/j.avb.2012.08.002

[cl21111-bib-0213] Gill, P. , Piazza, J. A. , & Horgan, J. (2016). Counterterrorism killings and provisional IRA bombings, 1970‐1998. Terrorism and Political Violence, 28(3), 473–496. 10.1080/09546553.2016.1155932

[cl21111-bib-0214] Gillespie, R. (2009). *Issues in pursuit of intelligence: Mind games or minefield?* (Master's thesis). Athabasca, AB, Canada, Athabasca University.

[cl21111-bib-0215] Giorgio, O. , Stefano, G. , & Rossana, G. (2018). Fondamentalisme et radicalisation chez les deuxième et troisième générations d'immigrés en Italie et en Europe: Profil psychopathologique et études de cas [Fundamentalism and radicalisation among second and third generation immigrants in Italy and in Europe: Psychopathological profile and case studies]. Rivista di Criminologia, 12(2), 27–45.

[cl21111-bib-0216] Gjerland, A. , Pedersen, M. J. B. , Ekeberg, Ø. , & Skogstad, L. (2015). Sick‐leave and help seeking among rescue workers after the terror attacks in Norway, 2011. International Journal of Emergency Medicine, 8(31), 81–88. 10.1186/s12245-015-0081-4 26283071 PMC4539308

[cl21111-bib-0217] Glässer, U. , Tayebi, M. A. , Brantingham, P. L. , & Brantingham, P. (2012). Estimating possible criminal organizations from co‐offending data (Report No. 29). Ottawa, ON, Canada: Public Safety Canada.

[cl21111-bib-0218] Global Centre on Cooperative Security . (2015). *The next decade: Strengthening multilateral efforts for preventing and countering violent extremism*. Retrieved from https://www.globalcenter.org/wp-content/uploads/2015/07/Global-Center_The-Next-Decade_UN-and-CT-CVE_Project-Description.pdf

[cl21111-bib-0219] Glomseth, R. , & Gottschalk, P. (2009). Police personnel cultures: A comparative study of counter terrorist and criminal investigation units. Criminal Justice Studies, 22(1), 3–15. 10.1080/14786010902796457

[cl21111-bib-0220] Goktepe, F. (2009). *Preparedness of Kentucky State Police against terrorism* (Doctoral dissertation). Retrieved from ProQuest Dissertations and Theses Global database. (UMI No. 3371756).

[cl21111-bib-0221] Golba, L. W. (2013). *Decision making processes of informant handlers* (Doctoral dissertation). Retrieved from ProQuest Dissertations and Theses Global database. (UMI No. 3589760)

[cl21111-bib-0222] González‐Ordi, H. , Cano‐Vindel, A. , Miguel‐Tobal, J. J. , & Iruarrizaga, I. (2004). Efectos de la exposición a eventos traumáticos en personal de emergencias: Consecuencias psicopatológicas tras el atentado terrorista del 11‐M en Madrid. Ansiedad y Estrés, 10(2‐3), 207–217.

[cl21111-bib-0223] Goodrum, A. A. (2007). Watching what we read: Implications of law enforcement activity in libraries since 9/11. In T. Loendorf & G. D. Garson (Eds.), Patriotic information systems (pp. 91–127). Hershey, PA: IGI Global.

[cl21111-bib-0224] Goodwill, A. M. , Alison, L. , Lehmann, R. , Francis, A. , & Eyre, M. (2010). The impact of outcome knowledge, role, and quality of information on the perceived legitimacy of lethal force decisions in counter‐terrorism operations. Behavioral Sciences & the Law, 28, 337–350. 10.1002/bsl.897 19877173

[cl21111-bib-0225] Government of Denmark . (2009). *A common and safe future: An action plan to prevent extremist views and radicalization among young people*. Retrieved from https://ec.europa.eu/migrant-integration/librarydoc/a-common-and-safe-future-an-action-plan-to-prevent-extremist-views-and-radicalization-among-young-people

[cl21111-bib-0226] de Graaf, B. A. , & de Graaff, B. G. J. (2008). Counterterrorism in the Netherlands after 9/11: The 'Dutch approach'. In J. Moran & M. Phythia (Eds.), Intelligence, security and policing post‐9/11 (pp. 183–202). London, UK: Palgrave Macmillan. 10.1057/9780230583542_10

[cl21111-bib-0227] Graham, D. (2018). Marauding terrorist firearms’ attacks: A practitioner's view of the UK's Emergency Service initial response arrangements to deal with an active shooter. RUSI Journal, 163(2), 42–50. 10.1080/03071847.2018.1464628

[cl21111-bib-0228] Granhag, P. A. , Oleszkiewicz, S. , & Kleinman, S. (2016). Eliciting information from small cells of sources. Journal of Policing, Intelligence, and Counter Terrorism, 11(2), 143–162. 10.1080/18335330.2016.1215507

[cl21111-bib-0229] Granhag, P. A. , Rangmar, J. , & Strömwall, L. A. (2015). Small cells of suspects: Eliciting cues to deception by strategic interviewing. Journal of Investigative Psychology & Offender Profiling, 12(2), 127–141. 10.1002/jip.1413

[cl21111-bib-0230] Greene, J. R. (2011). Community policing and terrorism: Problems and prospects for local community security. In B. Forst , J. R. Greene & J. P. Lynch (Eds.), Criminologists on terrorism and homeland security (pp. 208–244). Cambridge, UK: Cambridge University Press.

[cl21111-bib-0231] Greenfield, V. A. , Willis, H. H. & LaTourrette, T. (Eds.) (2012). Assessing the benefits of US customs and border protection regulatory actions to reduce terrorism risks. Santa Monica, CA: RAND Corporation.

[cl21111-bib-0232] Gromoglasova, E. (2017). Assessment of counterterrorist strategies—A spatial approach. Mezhdunarodnye Protsessy, 15(4), 133–155. 10.17994/IT.2017.15.4.51.8

[cl21111-bib-0233] Groupe Intersol Inc (2009). *Atelier sur les pratiques exemplaires relatives aux recherches en matière de lutte contre le crime organisé: Rapport sommaire*. Ottawa, ON, Canada: Sécurité Publique Canada.

[cl21111-bib-0234] Gruenewald, J. , Dooley, K. M. G. , Suttmoeller, M. J. , Chermak, S. M. , & Freilich, J. D. (2016). A mixed‐method analysis of fatal attacks on police by far‐right extremists. Police Quarterly, 19(2), 216–245. 10.1177/1098611115623061

[cl21111-bib-0235] Gruenewald, J. , Klein, B. R. , Drawve, G. , Smith, B. L. , & Ratcliff, K. (2018). Suspicious preoperational activities and law enforcement interdiction of terrorist plots. Policing, 42(1), 89–107. 10.1108/PIJPSM-08-2018-0125

[cl21111-bib-0236] Gruenewald, J. , Parkin, W. S. , Smith, B. L. , Chermak, S. M. , Freilich, J. D. , Roberts, P. , & Klein, B. (2015a). *Validation of the nationwide Suspicious Activity Reporting (SAR) initiative (Research Brief)*. College Park, MD: National Consortium for the Study of Terrorism and Responses to Terrorism.

[cl21111-bib-0237] Gruenewald, J. , Parkin, W. S. , Smith, B. L. , Chermak, S. M. , Freilich, J. D. , Roberts, P. , & Klein, B. (2015b). *Validation of the Nationwide Suspicious Activity Reporting (SAR) initiative: Identifying suspicious activities from the Extremist Crime Database (ECDB) and the American Terrorism Study (ATS)*. College Park, MD: National Consortium for the Study of Terrorism and Responses to Terrorism.

[cl21111-bib-0238] Gssime, Y. , & Meines, M. (2018). *Tabletop exercises: Practicing multi‐agency cooperation*. Paper presented at the RAN Local, Dublin, Ireland.

[cl21111-bib-0239] Gusty, D. , Magnussen, W. , Endrulat, E. , Sopel, J. , & Perlstein, S. (2018). *Canada‐U.S. enhanced resiliency experiment series (cause v) binational after action report*. Washington, DC: U.S. Department of Homeland Security. Retrieved from https://www.dhs.gov/sites/default/files/publications/881_CAUSE-V_Binational-After-Action-Report_180514-508.pdf

[cl21111-bib-0240] Gutauskas, A. , Kalesnykas, R. , & Petrosius, D. (2004). Problem of terrorism prevention in Lithuania. Jurisprudencija, 63(55), 24–45.

[cl21111-bib-0241] Haanstra, W. (2017). *Engaging with communities: Collaboration between local authorities and communities in PVE*. Paper presented at RAN LOCAL and YF&C, Prague, Czech Republic.

[cl21111-bib-0242] * Haanstra, W. (2018). *Engaging with communities: Collaboration between local authorities and communities in PVE*. Paper presented at RAN Centre of Excellence, Prague, Czech Republic.

[cl21111-bib-0243] Haar, R. J. , Iacopino, V. , Ranadive, N. , Dandu, M. , & Weiser, S. D. (2017). Death, injury and disability from kinetic impact projectiles in crowd‐control settings: A systematic review. BMJ Open, 7(12), 018154. 10.1136/bmjopen-2017-018154 PMC5736036 29255079

[cl21111-bib-0244] Hackman, R. J. (2011). Collaborative intelligence: Using teams to solve hard problems. San Francisco, CA: Berrett‐Koehler Publishers, Inc.

[cl21111-bib-0245] Hamm, M. S. , & Van de Voorde, C. (2005). Crimes committed by terrorist groups: Theory, research, and prevention. Trends in Organized Crime, 9(2), 18–50.

[cl21111-bib-0246] Hansen, A. S. (2002). *From Congo to Kosovo: Civilian police in peace operations* (Adelphi Paper No. 343). London, UK: Routledge.

[cl21111-bib-0247] Harris‐Hogan, S. (2012). *Domestic terror raids: A timely reminder of a persistent threat*. The Conversation. Retrieved from https://theconversation.com/domestic-terror-raids-a-timely-reminder-of-a-persistent-threat-9556

[cl21111-bib-0248] Harris‐Hogan, S. (2013). *Gun control could help the fight against homegrown terrorism*. The Conversation. Retrieved from https://theconversation.com/gun-control-could-help-the-fight-against-homegrown-terrorism-11611

[cl21111-bib-0249] Hasan, N. , Hendriks, B. , Janssen, F. , & Meijer, R. (2012). *Counter‐terrorism strategies in Indonesia, Algeria and Saudi Arabia*. The Hague, The Netherlands: Netherlands Institute of International Relations.

[cl21111-bib-0250] Hasisi, B. , & Weisburd, D. (2014). Policing terrorism and police‐community relations: Views of the Arab minority in Israel. Police Practice and Research, 15, 158–172. 10.1080/15614263.2013.874173

[cl21111-bib-0251] Havlickova, B. (2018, April). *Changes in consumer behavior when armed soldier is present in front of Museum Louvre*. Paper presented at the 23rd International Conference of Theoretical and Practical Aspects of Public Finance, Prague, Czech Republic.

[cl21111-bib-0252] Hawkins, K. , & Bigelow, J. (2005). Dealing with terror. Security Solutions, 37, 54–64.

[cl21111-bib-0253] Haynes, C. , & Mangas, J. (2015). *Countering extremism: An understanding of the problem, the process and some solutions* (Master's thesis). Retrieved from http://hdl.handle.net/10945/45871

[cl21111-bib-0254] Hearne, E. B. , & Laiq, N. (2010). *A new approach? Deradicalization programs and counterterrorism* (Meeting Note). Amman, Jordan: International Peace Institute.

[cl21111-bib-0255] Heath‐Kelly, C. (2013). Counter‐terrorism and the counterfactual: Producing the ‘radicalisation’ discourse and the UK PREVENT strategy. The British Journal of Politics and International Relations, 15(3), 394–415. 10.1111/j.1467-856X.2011.00489.x

[cl21111-bib-0256] Heinrich, D. P. , Thornton, A. E. , Morgan, R. M. , & Bouhana, N. (2013). An analysis of forensic evidence used in the prosecution of terrorism cases in Britain between 1972 and 2008. Policing, 7(1), 96–108. 10.1093/police/pas064

[cl21111-bib-0257] Helbing, D. , Brockmann, D. , Chadefaux, T. , Donnay, K. , Blanke, U. , Woolley‐Meza, O. , … Perc, M. (2015). Saving human lives: What complexity science and information systems can contribute. Journal of Statistical Physics, 158(3), 735–781. 10.1007/s10955-014-1024-9 26074625 PMC4457089

[cl21111-bib-0258] Helfand, N. S. , & Osborne, D. L. (2003). *Asian organized crime and terrorist activity in Canada, 1999‐2002*. Washington, DC: Federal Research Division, Library of Congress.

[cl21111-bib-0259] Helmus, T. C. , & Klein, K. (2018). *Assessing outcomes of online campaigns countering violent extremism: A case study of the redirect method*. Santa Monica, CA: RAND Corporation. Retrieved from https://www.rand.org/content/dam/rand/pubs/research_reports/RR2800/RR2813/RAND_RR2813.pdf

[cl21111-bib-0260] * Helmus, T. C. , Matthews, M. , Ramchand, R. , Beaghley, S. , Stebbins, D. , Kadlec, A. , … Acosta, J. D. (2017). *RAND program evaluation toolkit for countering violent extremism*. Santa Monica, CA: RAND Corporation.

[cl21111-bib-0261] van Hemert, D. , van den Berg, H. , van Vliet, T. , Roelofs, M. , & Huis in 't Veld, M. (2014). *Synthesis report on the state‐of‐the‐art in evaluating the effectiveness of counter‐violent extremism interventions* (Report No. 312235). Retrieved from http://impacteurope.eu/wp-content/uploads/2015/02/D2.2-Synthesis-Report.pdf

[cl21111-bib-0262] Henderson, N. J. , Ortiz, C. W. , Sugie, N. F. , & Miller, J. (2006). *Law enforcement and Arab American community relations after September 11, 2001*. New York, NY: Vera Institute of Justice.

[cl21111-bib-0263] Henry, V. E. (2002). The COMPSTAT paradigm: Management accountability in policing, business and the public sector. Flushing, NY: Looseleaf Law Publications.

[cl21111-bib-0264] Henry, V. E. (2002). The need for a coordinated and strategic local police approach to terrorism: A practitioner's perspective. Police Practice and Research, 3(4), 319–336. 10.1080/1561426022000032088

[cl21111-bib-0265] Hirschfield, A. , Christmann, K. , Wilcox, A. , Rogerson, M. , & Sharratt, K. (2012). *Process evaluation of preventing violent extremism programmes for young people*. London, UK: Youth Justice Board for England and Wales.

[cl21111-bib-0266] Ho, J. D. , Heegaard, W. G. , Dawes, D. M. , Natarajan, S. , Reardon, R. F. , & Miner, J. R. (2009). Unexpected arrest‐related deaths in America: 12 months of open source surveillance. Western Journal of Emergency Medicine, 10(2), 68–73. PMC2691515 19561821

[cl21111-bib-0267] * Hofman, J. , & Sutherland, A. (2018). *Evaluating interventions that prevent or counter violent extremism: A practical guide*. Santa Monica, CA: RAND Corporation.

[cl21111-bib-0268] Hoff, S. , Hollister, B. A. , Hodges, H. , & Scalora, M. (2013). *Assessing religious content in threatening and harassing communications toward political figures*. Paper presented at the American Psychology‐Law Society Annual Conference, Portland, OR.

[cl21111-bib-0269] Hoffmann, P. , & Terplan, K. (2006). Intelligence support systems: Technologies for lawful intercepts. Boca Raton, FL: Taylor & Francis Group.

[cl21111-bib-0270] Hoile, R. , Walsh, S. J. , & Roux, C. (2007). Bioterrorism: Processing contaminated evidence, the effects of formaldehyde gas on the recovery of latent fingermarks. Journal of Forensic Science, 52(5), 1097–1102. 10.1111/j.1556-4029.2007.00539.x 17767655

[cl21111-bib-0271] Holmer, G. (2013). *Countering violent extremism: A peacebuilding perspective* (Special Report No. 336). Washington, DC: United States Institute of Peace.

[cl21111-bib-0272] Horgan, J. , & Altier, M. B. (2012). *Arc of terrorist involvement*. International Center for the Study of Terrorism, Retrieved from https://www.dhs.gov/sites/default/files/publications/947_OPSR_TP_Arc-of-Terrorism-Involvement-Overview_2012-508_0.pdf

[cl21111-bib-0273] Horgan, J. G. , Gill, P. , Bouhana, N. , Silver, J. , & Corner, E. (2016). *Across the universe? A comparative analysis of violent radicalization across three offender types with implications for criminal justice training and education* (Report No. 249937). Washington, DC: National Institute of Justice. Retrieved from https://www.ncjrs.gov/pdffiles1/nij/grants/249937.pdf

[cl21111-bib-0274] Horgan, J. G. , Williams, M. J. , Evans, W. P. , & Belanger, J. J. (2017). *Assessment report: Current capabilities of 2‐1‐1 call centers and local service providers*. Atlanta, GA: Georgia State University Research Foundation, Inc. Retrieved from https://www.dhs.gov/sites/default/files/publications/OPSR_TP_Assessment-Report_Current-Capabilities-211-Call-Centers_Local-Service-Providers_508.pdf

[cl21111-bib-0275] Hossain, L. , & Kuti, M. (2010). Disaster response preparedness coordination through social networks. Disasters, 34(3), 755–786. 10.1111/j.1467-7717.2010.01168.x 20345465

[cl21111-bib-0276] Howie, L. (2009). *Terrorism, the worker, and the city: Simulations and security in a time of terror*. Farnham, UK: Gower Publishing Limited.

[cl21111-bib-0277] Hughes, E. L. (Ed.). (2008). Report on the Sixth International Law Enforcement Forum for Minimal Force Options and Less‐Lethal Technologies. University Park, PA: Institute for Non‐Lethal Defence Technologies.

[cl21111-bib-0278] van der Hulst, R. C. (2008). Sociale netwerkanalyse en de bestrijding van criminaliteit en terrorisme [Theory and characteristics of social network analysis; implications for the combat of crime and terrorism]. Justitiële Verkenning, 34(5), 10–32.

[cl21111-bib-0279] Hunt, R. C. , Kapil, V. , Basavaraju, S. V. , Sasser, S. M. , McGuire, L. C. , & Sullivent, E. E. (2010). *Updated in a moment's notice: Surge capacity for terrorist bombings. Challenges and proposed solutions*. Atlanta, GA: Centers for Disease Control and Prevention.

[cl21111-bib-0280] Huq, A. Z. , Tyler, T. R. , & Schulhofer, S. J. (2011). Mechanisms for eliciting cooperation in counterterrorism policing: Evidence from the United Kingdom. Journal of Empirical Legal Studies, 8(4), 728–761. 10.1111/j.1740-1461.2011.01239.x

[cl21111-bib-0281] Hurwitz, J. , & Peffley, M. (2010). And justice for some: Race, crime, and punishment in the US criminal justice system. Canadian Journal of Political Science, 43(2), 457–479. 10.1017/S0008423910000120

[cl21111-bib-0282] Iacobucci, F. (2008). *Internal inquiry into the actions of Canadian officials in relation to Abdullah Almalki, Ahmad Abou‐Elmaati and Muayyed Nureddin*. Ottawa, ON, Canada: Public Works and Government Services Canada.

[cl21111-bib-0283] Iacobucci, F. (2010). *Internal inquiry into the actions of Canadian officials in relation to Abdullah Almalki, Ahmad Abou‐Elmaati and Muayyed Nureddin: Supplement to public report*. Ottawa, ON: Public Works and Government Services Canada.

[cl21111-bib-0284] IACP Committee on Terrorism, Countering Violent Extremism Working Group . (2012). *Community outreach and engagement principles*. Retrieved from http://www.theiacp.org/portals/0/pdfs/IACPCOT_CommPolicingPrinciples__FINALAug12.pdf

[cl21111-bib-0285] Institute for the Prevention of Crime . (n.d). *Making cities safer: International strategies and practices* (Report No. 1). Ottawa, ON, Canada.

[cl21111-bib-0286] Intelligence and Security Committee . (2009). *Could 7/7 have been prevented? Review of the intelligence on the London terrorist attacks on 7 July 2005*. Retrieved from http://www.fas.org/irp/world/uk/july7review.pdf

[cl21111-bib-0287] International Association of Chiefs of Police (2005). *Post 9‐11 policing: The crime control‐homeland security paradigm: Taking command of new realities*. Washington, DC: Bureau of Justice Assistance, Office of Justice Programs, U.S. Department of Justice.

[cl21111-bib-0288] International Association of Chiefs of Police (2014). *Online radicalization to violent extremism (Awareness Brief)*. Washington, DC: Community Oriented Policing Services.

[cl21111-bib-0289] International Association of Chiefs of Police (2014). *Using community policing to counter violent extremism: Five key principles for law enforcement*. Washington, DC: Community Oriented Policing Services US Department of Justice.

[cl21111-bib-0290] Jackson, B. A. (2014). *How do we know what information sharing is really worth?, Exploring methodologies to measure the value of information sharing and fusion efforts*. Santa Monica, CA: RAND Corporation. Retrieved from https://www.rand.org/pubs/research_reports/RR380.html

[cl21111-bib-0291] Jackson, B. A. , Faith, K. S. , & Willis, H. H. (2010). *Evaluating the reliability of emergency response systems for large‐scale incident operations*. Santa Monica, CA: RAND Corporation. 10.7249/rhq-02-03-08 PMC4945240 28083267

[cl21111-bib-0292] Jackson, B. A. , Peterson, D. J. , Bartis, J. T. , LaTourrette, T. , Brahmakulam, I. , Houser, A. , & Sollinger, J. (2002). *Protecting emergency responders: Lessons learned from terrorist attacks*. Santa Monica, CA: RAND Corporation.

[cl21111-bib-0293] Jaffrelot, C. (2010). La dialectique des terrorismes en Inde depuis 2001: la "main de l'étranger", les islamistes et les nationalistes hindous [The dialectics of terrorism in India since 2001: The 'foreign hand', Islamism and Hindu nationalists]. Critique Internationale, 2, 93–110.

[cl21111-bib-0294] Jaggers, B. (2008). *Anti‐terrorism control orders in Australia and the United Kingdom: A comparison*. Canberra, ACT, Australia: Parliamentary Library, Deptartment of Parliamentary Services.

[cl21111-bib-0295] Jaloszynski, K. (2013). Organisation of police negotiations in the Polish Police Service. Internal Security, 5(2), 153–161. 10.5604/20805268.1094136

[cl21111-bib-0296] James, L. , James, S. , & Vila, B. (2017). Does the “reverse racism effect” withstand the test of police officer fatigue? Policing, 40(2), 184–196. 10.1108/PIJPSM-01-2016-0006

[cl21111-bib-0297] Jefferson, K. A. (2015). *What's in a name: A comparative analysis of the United States’ Real ID Act and the United Kingdom's National Identity Scheme* (Master's thesis). Naval Postgraduate School, Monterey, CA.

[cl21111-bib-0298] Jenkins, B. M. , Liepman, A. , & Willis, H. H. (2014). *Identifying enemies among us: Evolving terrorist threats and the continuing challenges of domestic intelligence collection and information sharing*. Santa Monica, CA: RAND Corporation.

[cl21111-bib-0299] Jensen, M. , & LaFree, G. (2016). *Final report: Empirical assessment of domestic radicalization (EADR)* (Document No. 250481). Retrieved from https://www.ncjrs.gov/pdffiles1/nij/grants/250481.pdf

[cl21111-bib-0300] Joint Committee on Human Rights (2003). *Continuance in force of Sections 21 to 23 of the Anti‐terrorism, Crime and Security Act 2001* (Session 2002‐03 Report No. 5). London, UK: The Stationery Office.

[cl21111-bib-0301] Joint Committee on Human Rights (2004). Anti‐terrorism, crime and security act 2001: Statutory review and continuance of part 4 (Session 2003‐04 Report No. 6). London, UK: The Stationery Office.

[cl21111-bib-0302] Jonathan‐Zamir, T. , & Weisburd, D. (2009). *Does police performance increase in importance for the public during times of security threats, and do evaluations of procedural justice decline in importance? Findings from a quasi‐experimental study of antecedents of police legitimacy in Israel*. Jerusalem, Israel: Hebrew University.

[cl21111-bib-0303] Jonathan‐Zamir, T. , Weisburd, D. , & Hasisi, B. (2014). *Policing terrorism, crime control, and police‐community relations: Learning from the Israeli experience*. Cham, Switzerland: Springer International Publishing.

[cl21111-bib-0304] Jones, S. G. , & Libicki, M. C. (2008). *How terrorist groups end: Lessons for countering Al Qa'ida*. Santa Monica, CA: RAND Corporation.

[cl21111-bib-0305] Kada, J. , Dooley, K. , Linn, B. , & Mackanin, T. (2018). *Activation of body‐worn cameras without responder manipulation: Operational field assessment report*. New York, NY: National Urban Security Technology Laboratory (NUSTL). Retrieved from https://www.dhs.gov/sites/default/files/publications/1000_NUSTL-R-Tech_ABWC-OpExReport_1807-508.pdf

[cl21111-bib-0306] Karmani, A. (2009). *Reducing the influences of radicalisation for prisoners: An EU funded project in conjunction with London and West Yorkshire probation*. London, UK: ACPO.

[cl21111-bib-0307] Kaza, S. , Wang, Y. , & Chen, H. (2007). Enhancing border security: Mutual information analysis to identify suspect vehicles. Decision Support Systems, 43(1), 199–210. 10.1016/j.dss.2006.09.007

[cl21111-bib-0308] Kealey, G. S. (2017). *Spying on Canadians: The Royal Canadian Mounted Police security service and the origins of the long cold war*. Toronto, ON, Canada: University of Toronto Press.

[cl21111-bib-0309] * Kearns, E. M. (2018). Exploring officer views of community policing in counterterrorism. Police Practice and Research, 21(1), 18–32. 10.1080/15614263.2018.1428900

[cl21111-bib-0310] Kelly, R. W. (2010). The New York City Police Department's international perspective. Revue d'Etudes Constitutionnelles et Politiques, 132(1), 117–126.

[cl21111-bib-0311] Kennedy, J. W. , Lisanti, D. , & Fisher Lui, B. (2012). *Countermeasures against extremism and terrorism database: Data status and final findings: Final report to START*. College Park, MD: START.

[cl21111-bib-0312] Kerry, A. (2007). *2007‐2008 integrated summative evaluation of the chemical, biological, radiological and nuclear first responder training program*. Ottawa, ON, Canada: Canadian Emergency Management College, Public Safety Canada.

[cl21111-bib-0313] Khanov, T. A. , Sikhimbayev, M. R. , Birzhanov, B. K. , & Birzhanov, K. K. (2016). Genomic registration as a universal personal identifier in crime prevention: The research and prospects of introduction. Russian Journal of Criminology, 10(3), 544–553.

[cl21111-bib-0314] Khashu, A. , Busch, R. , & Latif, Z. (2005). *Building strong police‐immigrant community relations: Lessons from a New York City project*. Washington, DC: Community Oriented Policing Services, U.S. Department of Justice.

[cl21111-bib-0315] Khatab, S. (2008). *Towards a general counterterrorism strategy*. Paper presented at the 2008 GTReC International Conference, Melbourne, VIC, Australia.

[cl21111-bib-0316] Khrustaleva, N. S. , Rezler, M. I. , & Ishutkina, A. A. (2010). Training of negotiators for emergency situations. Vestnik Sankt‐Peterburgskogo Universiteta, 12, 55–58.

[cl21111-bib-0317] Kierstead, W. (2006). *Law enforcement safety handbook: Unusual weapons, concealment methods for contraband & things that make you wonder why you ever became a cop*. Brunswick, ME: Brunswick Police Department.

[cl21111-bib-0318] Kılıçlar, A. , Uşaklı, A. , & Tayfun, A. (2018). Terrorism prevention in tourism destinations: Security forces vs. civil authority perspectives. Journal of Destination Marketing & Management, 8, 232–246. 10.1016/j.jdmm.2017.04.006

[cl21111-bib-0319] Kimunguyi, P. (2010). *Terrorism and counter terrorism in East Africa*. Paper presented at the GTReC ARC Linkage Project on Radicalisation Conference 2010: Understanding Terrorism from an Australian Perspective, Caulfield, VIC, Australia.

[cl21111-bib-0320] Kirilenko, V. P. , & Alekseev, G. V. (2018). Actual problems of extremism crime counteraction. Russian Journal of Criminology, 12(4), 561–571. 10.17150/2500-4255.2018.12(4).561-571

[cl21111-bib-0321] Klick, J. , & Tabarrok, A. (2003). Using terror alert levels to estimate the effect of police on crime. Rochester, NY: Social Science Research Network.

[cl21111-bib-0322] Klick, J. , & Tabarrok, A. (2005). Using terror alert levels to estimate the effect of police on crime. Journal of Law & Economics, 48(1), 267–279. 10.1086/426877

[cl21111-bib-0323] Kochoi, S. M. (2018). Antiterrorism norms in the criminal code of the Russian federation and the practice of their enforcement. Russian Journal of Criminology, 12(2), 258–265. 10.17150/2500-4255.2018.12(2).258-265

[cl21111-bib-0324] Koestner, L. G. (2008). Law enforcement online. FBI Law Enforcement Bulletin, 77(10), 21.

[cl21111-bib-0325] Kolb, N. & Roberts, D. (Eds.). (2013). Special issue. *The Police Chief*, 80(6).

[cl21111-bib-0326] Koren, E. (2014). *Centres de fusion dans les pays sélectionnés* (Resume de recheche no. 24). Ottawa, ON, Canada: Sécurité Publique Canada.

[cl21111-bib-0327] Krafchik, M. , & Ryszkowska, Y. (2011). *Evaluation of Young and Safe project: London borough of Lambeth (Evaluation report)*. West Midlands, UK: Inspira Consulting.

[cl21111-bib-0328] Kurzman, C. (2011). *Muslim‐American terrorism since 9/11: An accounting*. Triangle Centre on Terrorism and Homeland Security. Retrieved from http://kurzman.unc.edu/files/2011/06/Kurzman_Muslim-American_Terrorism_Since_911_An_Accounting.pdf

[cl21111-bib-0329] Kurzman, C. (2012). *Muslim‐American terrorism in the decade since 9/11*. Triangle Centre on Terrorism and Homeland Security. Retrieved from http://kurzman.unc.edu/files/2011/06/Kurzman_Muslim-American_Terrorism_in_the_Decade_Since_9_11.pdf

[cl21111-bib-0330] Kurzman, C. (2013). *Muslim‐American terrorism declining further*. Triangle Centre on Terrorism and Homeland Security. Retrieved from https://kurzman.unc.edu/files/2011/06/Kurzman_Muslim-American_Terrorism_February_1_2013.pdf

[cl21111-bib-0331] Kurzman, C. (2014). *Despite Boston, number of Muslim‐American terrorists remains low*. Triangle Centre on Terrorism and Homeland Security. Retrieved from https://sites.duke.edu/tcths/files/2013/06/Kurzman_Muslim-American_Terrorism_in_20131.pdf

[cl21111-bib-0332] Kurzman, C. (2015). *Terrorism cases involving Muslim‐Americans, 2014*. Triangle Centre on Terrorism and Homeland Security. Retrieved from https://sites.duke.edu/tcths/files/2013/06/Kurzman_Terrorism_Cases_Involving_Muslim-Americans_2014.pdf

[cl21111-bib-0333] Kurzman, C. (2016). *Muslim‐American involvement in violent extremism in 2015*. Triangle Centre on Terrorism and Homeland Security. Retrieved from https://sites.duke.edu/tcths/files/2013/06/Kurzman_Muslim-American_Involvement_in_Violent_Extremism_2015.pdf

[cl21111-bib-0334] Kurzman, C. (2017). *Muslim‐American involvement with violent extremism, 2016*. Triangle Centre on Terrorism and Homeland Security. Retrieved from https://sites.duke.edu/tcths/files/2017/01/FINAL_Kurzman_Muslim-American_Involvement_in_Violent_Extremism_2016.pdf

[cl21111-bib-0335] Kurzman, C. (2018). *Muslim‐American involvement with extremism, 2017*. Triangle Centre on Terrorism and Homeland Security. Retrieved from https://sites.duke.edu/tcths/files/2018/01/Kurzman_Muslim-American_Involvement_with_Violent_Extremism_2017.pdf

[cl21111-bib-0336] Kurzman, C. , & Schanzer, D. (2015). *Law enforcement assessment of violent extremism threat*. Triangle Centre on Terrorism and Homeland Security. Retrieved from https://sites.duke.edu/tcths/files/2013/06/Kurzman_Schanzer_Law_Enforcement_Assessment_of_the_Violent_Extremist_Threat_final.pdf

[cl21111-bib-0337] Kwon, I.‐W. G. , & Baack, D. W. (2005). Focus on government intervention: The effectiveness of legislation controlling gun usage. A holistic measure of gun control legislation. American Journal of Economics and Sociology, 64(2), 533–547.

[cl21111-bib-0338] LaFree, G. (2012). Ideas in American policing: Policing terrorism. Police Foundation, 15, 1–12.

[cl21111-bib-0339] LaFree, G. , & Dugan, L. (2009). Research on terrorism and countering terrorism. Crime and Justice, 38(1), 413–477.

[cl21111-bib-0340] Lafree, G. , & Hendrickson, J. (2007). Build a criminal justice policy for terrorism. Criminology & Public Policy, 6, 781–790. 10.1111/j.1745-9133.2007.00471.x

[cl21111-bib-0341] Lai, C.‐P. (2007). *Through wall surveillance using ultrawideband random noise radar* (Doctoral dissertation). Retrieved from ProQuest Dissertations and Theses Global database. (UMI No. 3284959).

[cl21111-bib-0342] Lane, S. , Rahilly, L. , & Teufel, H., III (2008). *Privacy impact assessment for the law enforcement intelligence fusion system (IFS)*. Washington, DC: U.S. Department of Homeland Security. Retrieved from https://www.dhs.gov/sites/default/files/publications/ice-pia-007-IFS-2008.pdf

[cl21111-bib-0343] Lee, E. (2017). Hate perspective on terror: Domestic and international. In E. Dunbar , A. Blanco , D. A. Crèvecoeur‐MacPhail , C. Munthe , M. Fingerle & D. Brax (Eds.), The psychology of hate crimes as domestic terrorism: U.S. and global issues: Interventions, treatment, and management (pp. 37–61). Santa Barbara, CA: Praeger.

[cl21111-bib-0344] Legault, R. , & Deloughery, K. (2015). *Countering violent extremism—Developing a research roadmap: Stakeholder recruitment and engagement plan*. RTI International, Retrieved from https://www.dhs.gov/sites/default/files/publications/OPSR_TP_CVE-Developing-Research-Roadmap_Stakeholder-Recruitment-Engagement-Plan_201512-508.pdf

[cl21111-bib-0345] Lemieux, F. (2010). International police cooperation: Emerging issues, theory and practice. Cullompton, UK: Willan Publishing.

[cl21111-bib-0346] Lenos, S. , & Haanstra, W. (2017a). *The role of police officers in dealing with jihadist returnees*. Paper presented at RAN POL, Dusseldorf, Germany.

[cl21111-bib-0347] Lenos, S. , & Haanstra, W. (2017a). *Police, families and family workers: How to foster closer engagement with families and family workers, and why*. Paper presented at RAN POL, Lisbon, Portugal.

[cl21111-bib-0348] * Lenos, S. , & Keltjens, M. (2016a). *Preparing RAN POL's guide on training programmes for police officers in Europe*. Paper presented at RAN POL, Athens, Greece.

[cl21111-bib-0349] * Lenos, S. , & Keltjens, M. (2016b). RAN POL's Guide on training programmes for police officers in Europe . Paper presented at RAN POL, Athens, Greece.

[cl21111-bib-0350] Lenos, S. , & Keltjens, M. (2016c). The role for police officers in multi‐agency working and information sharing . Paper presented at RAN POL, Utrecht, The Netherlands.

[cl21111-bib-0351] Lenos, S. , & Keltjens, M. (2016d). The school needs partners . Paper presented at RAN EDU Meeting, Madrid, Spain.

[cl21111-bib-0352] Lenos, S. , & Wouterse, L. (2018a). *Police prevention and countering of far‐right and far‐left extremism*. Paper presented at RAN POL, Rome, Italy.

[cl21111-bib-0353] Lenos, S. , & Wouterse, L. (2018b). *Lessons from crime prevention: RAN POL engages at the annual international German Congress on Crime Prevention*. Paper presented at RAN POL, Dresden, Germany.

[cl21111-bib-0354] Lenos, S. , & Wouterse, L. (2018c). The role of police online in PVE and CVE It takes a network to defeat an extremist network. Paper presented at RAN POL, Oslo, Norway.

[cl21111-bib-0355] Lentini, P. (2007). Countering terrorism as if Muslims matter: Cultural citizenship and civic pre‐emption in anti‐terrorism. In L. Holmes (Ed.), Terrorism, organised crime and corruption: networks and linkages (pp. 42–59). Cheltenham, UK: Edward Elgar.

[cl21111-bib-0356] Lentini, P. (2008). Understanding and combating terrorism: Definitions, origins and strategies. Australian Journal of Political Science, 43, 133–140. 10.1080/10361140701842615

[cl21111-bib-0357] Lentini, P. (2010). *If they know who put the sugar it means they know everything: Understanding terrorist activity using Operation Pendennis wiretap (listening device and telephone intercept) transcripts*. Paper presented at the GTReC ARC Linkage Project on Radicalisation Conference, Caulfield, VIC, Australia.

[cl21111-bib-0358] Leuprecht, C. , & Speer, S. (2016). *From a mandate for change to a plan to govern: Defending freedoms by effectively countering terrorism* (Series No. 9). Retrieved from https://www.macdonaldlaurier.ca/files/pdf/MLICommentarySpeerLeuprecht-02-16-webready-v2.pdf

[cl21111-bib-0359] Levine, E. S. , Tisch, J. , Tasso, A. , & Joy, M. (2017). The New York City Police Department's domain awareness system. INFORMS Journal on Applied Analytics, 47(1), 70–84. 10.1287/inte.2016.0860

[cl21111-bib-0360] Levitt, M. (2017). *Defeating ideologically inspired violent extremism: A strategy to build strong communities and protect the U.S. homeland* (Policy note No. 37). Washington, DC: The Washington Institute for Near East Policy. Retrieved from https://www.washingtoninstitute.org/uploads/Documents/pubs/Transition2017-CVE-6.pdf

[cl21111-bib-0361] Liht, J. , & Savage, S. (2013). Preventing violent extremism through value complexity: Being Muslim being British. Journal of Strategic Security, 6(4), 44–66. 10.5038/1944-0472.6.4.3

[cl21111-bib-0362] Likar, B. (2010). Ne “mitingu resnice” [No to the rally of truth]. Varstvoslovje, 12(1), 72–85.

[cl21111-bib-0363] Lin, S.‐W. , Li, C. , & Ip, S. C. Y. (2017). A selection guide for the new generation 6‐dye DNA profiling systems. Forensic Science International: Genetics, 30, 34–42. 10.1016/j.fsigen.2017.05.010 28609758

[cl21111-bib-0364] Liu, B. F. , & Smarick, K. (2012). *Law enforcement efforts to counter violent extremism: Lessons learned from past cases*, National Consortium for the Study of Terrorism and Responses to Terrorism. Retrieved from https://start.umd.edu/news/research-brief-law-enforcement-efforts-counter-violent-extremism

[cl21111-bib-0365] Livermore, D. (2018). Detained Islamic fundamentalist extremism and the war on terror in Canada. Montreal, QC, Canada: McGill‐Queen's University Press.

[cl21111-bib-0366] L'Office des Nations Unies contre la drogue et le crime (2004). *Guide législatif sur les conventions et protocoles mondiaux contre le terrorisme*. New York, NY: United Nations.

[cl21111-bib-0367] Lowe, D. , Turk, A. & Das, D. (Eds.). (2014). Examining political violence. New York, NY: Routledge.

[cl21111-bib-0368] Lu, H. L. , & Bin Taylor, M. (2010). A comparative analysis of cybercrimes and governmental law enforcement in China and the United States. Asian Journal of Criminology, 5(2), 123–135. 10.1007/s11417-010-9092-5

[cl21111-bib-0369] Lučić, I. (2010). Croatian anticommunist guerilla in Bosnia‐Herzegovina, 1945‐1951. Casopis za Suvremenu Povijest, 42(3), 631–670.

[cl21111-bib-0370] Luke, T. J. , Hartwig, M. , Brimbal, L. , Chan, G. , Jordan, S. , Joseph, E. , … Granhag, P. A. (2013). Interviewing to elicit cues to deception: Improving strategic use of evidence with general‐to‐specific framing of evidence. Journal of Police and Criminal Psychology, 28(1), 54–62. 10.1007/s11896-012-9113-7

[cl21111-bib-0371] Lum, C. , & Kennedy, L. W. (2012). In support of evidence‐based approaches: A rebuttal to Gloria Laycock. Policing, 6(4), 317–323. 10.1093/police/pas041

[cl21111-bib-0372] Lum, C. , Kennedy, L. W. , & Sherley, A. (2006). Are counter‐terrorism strategies effective? The results of the Campbell systematic review on counter‐terrorism evaluation research. Journal of Experimental Criminology, 2(4), 489–516. 10.1007/s11292-006-9020-y

[cl21111-bib-0373] Lum, C. , Kennedy, L. W. , & Sherley, A. J. (2006). The effectiveness of counter‐terrorism strategies. Campbell Systematic Reviews, 2(2), 1–56. 10.4073/csr.2006.2

[cl21111-bib-0374] Lundberg, R. (2013). *Comparing homeland security risks using a deliberative risk ranking methodology* (Doctoral dissertation). The Pardee RAND Graduate School, Santa Monica, CA.

[cl21111-bib-0375] Lynn, P. (2005). *Mutual aid: Multijurisdictional partnerships for meeting regional threats* (Report No. NCJ210679). Washington, DC: Bureau of Justice Association.

[cl21111-bib-0376] MacFarlane, B. (2011). *Online violent radicalisation (OVeR): Challenges facing law enforcement agencies and policy stakeholders*. Paper presented at the GTReC ARC Linkage Project on Radicalisation Conference, Melbourne, VIC, Australia.

[cl21111-bib-0377] MacPherson, D. (2006). *Mapping security: A network analysis* (Master's thesis). Dalhousie University, Halifax, NS, Canada.

[cl21111-bib-0378] Madriaza, P. , Valendru, F. , Stock‐Rabbat, L. , Ponsot, A. S. , & Marion, D. (2017). *Dispositif d'intervention sur la radicalisation violente en milieu ouvert: Identification des difficultés et des besoins des professionnels des SPIP, aide à l'adaptation des pratiques*. Bordeaux, France: International Centre for the Prevention of Crime.

[cl21111-bib-0379] Maher, S. , & Frampton, M. (2009). *Choosing our friends wisely: Criteria for engagement with Muslim groups*. London, UK: Policy Exchange.

[cl21111-bib-0380] Maillard, J. , & Wyvekens, A. (2008). The Europe of interior security. Problèmes Politiques et Sociaux, 945, 5–119.

[cl21111-bib-0381] Making Manchester Safer . (2018). *RADEQUAL—Rethinking radicalisation programme of community engagement*. Retrieved from http://www.makingmanchestersafer.com/info/18/radequal

[cl21111-bib-0382] Malaver, A. P. , Poveda, M. V. P. , Cuéllar, F. J. P. , & Jiménez, J. S. P. (2018). Police service contribution to the conformation of the Nation‐State during the armed conflict in Colombia, from 1970 to 2006. Revista Criminalidad, 60(1), 59–78.

[cl21111-bib-0383] Marcoci, P. M. , & Grigore, F. D. (2012). Structuri informative românesti versus structuri informative ale unor state europene. Revista de Investigare a Criminalitatii, 5(1), 108–114.

[cl21111-bib-0384] Marion, N. , & Cronin, K. (2009). Law enforcement responses to Homeland Security Initiatives: The case of Ohio. Southwest Journal of Criminal Justice, 6(1), 4–24.

[cl21111-bib-0385] Marquis, G. (2015). *L’œil vigilant: les Services de police au Canada de la Confédération au 11 septembre [The vigilant eye: Policing in Canada to confederation to 9/11]*. Public Safety Canada Library.

[cl21111-bib-0386] Marrett, J.‐L. (2018). Dealing with violent extremist and terrorist offenders: Formalising cooperation among police, prison, probation and prosecution. Paper presented at RAN Policy and Practice Event, Paris, France.

[cl21111-bib-0387] Marret, J.‐L. , Bellasio, J. , van den Berg, H. , van Gorp, A. , van Hemert, D. , Leone, L. , … Wonderen, R. V. (2017). *Innovative methods and procedures to assess counter‐violent‐radicalisation techniques in Europe* (Report No. 312235). Impact Europe. Retrieved from http://impacteurope.eu/wp-content/uploads/2017/11/D6.2-Final-Synthesis-report.pdf

[cl21111-bib-0388] Masoumi, A. (2006). *The United Nations and international terrorism* (Master's thesis). Carleton University, Ottawa, ON, Canada.

[cl21111-bib-0389] Mastroe, C. (2016). Evaluating CVE: Understanding the recent changes to the United Kingdom's implementation of Prevent. Perspectives on Terrorism, 10(2), 50–60.

[cl21111-bib-0390] Mathieu, L. (2013). L'autre côté de la barricade: Perceptions et pratiques policières en mai et juin 1968 [The other side of the barricade: Police perceptions and practices in May and June 1968]. Revue Historique, 665(1), 145–172.

[cl21111-bib-0391] Mátyás, S. (2013). Disaster management, spatial planning and regional development. Tér és Társadalom, 27(3), 75–92.

[cl21111-bib-0392] Mayorkas, A. N. (2015). *Department policy regarding the use of cell‐site simulator technology*. Washington, DC: U.S. Department of Homeland Security. Retrieved from https://www.dhs.gov/sites/default/files/publications/Department%20Policy%20Regarding%20the%20Use%20of%20Cell-Site%20Simulator%20Technology.pdf

[cl21111-bib-0393] McCauley, C. (2009). Ware versus criminal justice in response to terrorism: The losing logic of torture. In W. Stritzke , S. Lewandowsky , D. Denemark , J. Clare & F. Morgan (Eds.), Terrorism and torture: An interdisciplinary perspective (pp. 63–85). Cambridge, UK: Cambridge University Press. 10.1017/CBO9780511581199.005

[cl21111-bib-0394] McDowell, D. (2009). Strategic intelligence: A handbook for practitioners, managers, and users. Lanham, MD: Scarecrow Press, Inc.

[cl21111-bib-0395] * McGarrell, E. F. , Freilich, J. D. , & Chermak, S. (2007). Intelligence‐led policing as a framework for responding to terrorism. Journal of Contemporary Criminal Justice, 23, 142–158. 10.1177/1043986207301363

[cl21111-bib-0396] McGoldrick, S. K. & McCardle, A. (Eds.). (2006). Uniform behavior: Police localism and national politics. New York, NY: Palgrave MacMillan.

[cl21111-bib-0397] McNamara, L. (2009). Closure, caution and the question of chilling: How have Australian counter‐terrorism laws affected the media? Media and Arts Law Review, 14(1), 1–30.

[cl21111-bib-0398] Meine, M. F. , & Dunn, T. P. (2013). Police consolidation in the United States: The never ending debate‐fiscal imperatives versus political agendas and public policy implications. In X. N. Zhu & S. R. Zhao (Eds.), Proceedings of 2013 International Conference on Public Administration (9th) (1, pp. 391–400). Chengdu, China: University of Electronic Science and Technology of China Press.

[cl21111-bib-0399] Merkur'ev, V. V. (2013). Criminologic characteristics of organized resistance to crime counteraction. Criminology Journal of Baikal National University of Economics and Law, 3, 25–41.

[cl21111-bib-0400] Metcalfe, C. , & Hodge, O. (2018). Empowering the police to fight terrorism in Israel. Criminology & Criminal Justice, 18(5), 585–603. 10.1177/1748895817739664

[cl21111-bib-0401] Meyer, S. (2012). Reducing harm from explosive attacks against railways. Security Journal, 25(4), 309–325. 10.1057/sj.2011.23

[cl21111-bib-0402] Michaelson, C. (2008). Law, intelligence and politics in Australia's 'war on terror'. In J. Moran & M. Phythian (Eds.), *Intelligence, security and policing post‐9/11: the UK's response to the 'war on* terror' (pp. 159–182). London, UK: Palgrave Macmillan. 10.1057/9780230583542_9

[cl21111-bib-0403] Mijares, T. C. , & McCarthy, R. M. (2008). The management of police specialized tactical units. Springfield, IL: Charles C Thomas Publishing.

[cl21111-bib-0404] Mikhailov, M. A. , & Kryazhev, V. S. (2016). The uniformity of defining the crimes of terrorism and extremism as a prerequisite for the effective methodology of their investigation. Russian Journal of Criminology, 10(4), 770–778.

[cl21111-bib-0405] Millar, A. (2009). Developing regional counterterrorism cooperation in South Asia. CTC Sentinel, 2(12), 18–21.

[cl21111-bib-0406] Millar, A. (2010). *Multilateral counterterrorism: Harmonizing political direction and technical expertise*. The Stanley Foundation, Policy Analysis Brief. Retrieved from https://www.globalcenter.org/wp-content/uploads/2012/07/MillarPAB1210.pdf

[cl21111-bib-0407] Millar, A. , & Fink, N. C. (2016). *Blue Sky III: Taking UN counterterrorism efforts in the next decade from plans to action*. Global Center of Cooperative Security, Norwegian Ministry of Foreign Affairs. Retrieved from https://www.globalcenter.org/wp-content/uploads/2016/09/Blue-Sky-III_low-res.pdf

[cl21111-bib-0408] Millar, A. , Fink, N. C. , Boucher, A. , Ipe, J. , Shetret, L. , & Schwartz, M. (2013). *Countering violent extremism and promoting community engagement in West Africa and the Sahel: An action agenda*. The Center on Global Counterterrorism Cooperation. Retrieved from https://www.globalcenter.org/wp-content/uploads/2013/07/Action-Agenda-ENG.pdf

[cl21111-bib-0409] Millar, A. , Ipe, J. , Cortright, D. , Lopez, G. A. , Marbarger, A. , & Lawall, K. (2006). *Report on standards and best practices for improving states’ implementation of UN Security Council counter‐terrorism mandates*. Center on Global Count‐Terrorism Cooperation. Retrieved from https://www.globalcenter.org/wp-content/uploads/2006/09/best_pratices.pdf

[cl21111-bib-0410] * Miller, E. , Toliver, J. , & Schanzer, D. (2016). *Promising practices for using community policing to prevent violent extremism: How to create and implement a community outreach program*. Police Executive Research Forum, Triangle Center on Terrorism and Homeland Security. Retrieved from https://sites.duke.edu/tcths/files/2016/06/manual_final.pdf

[cl21111-bib-0411] Millie, A. & Das, K. D. (Eds.). (2008). Contemporary issues in law enforcement and policing. Boca Raton, FL: CRC Press.

[cl21111-bib-0412] von Minor, D. (2016). *MAXIME Berlin — Interkulturelles und interreligiöses präventionsprojekt zur toleranzund demokratieentwicklung: Evaluation 2014–2016*. Berlin, Germany: Violence Prevention Network.

[cl21111-bib-0413] Mirahmadi, H. , & Farooq, M. (2010). *A community based approach to countering radicalization: A partnership for America*. Washington, DC: World Organization for Resource Development and Education.

[cl21111-bib-0414] Mitchell, S. (2016). Deradicalization: Using triggers for the development of a US program. Journal for Deradicalization, 9, 101–125.

[cl21111-bib-0415] Mitts, T. (2017). *Do community engagement efforts reduce extremist rhetoric on social media?* Retrieved from https://ssrn.com/abstract=2940290

[cl21111-bib-0416] Mockaitis, T. (2003). Winning hearts and minds in the ‘war on terrorism’. Small Wars and Insurgencies, 14(1), 21–38. 10.1080/09592310412331300546

[cl21111-bib-0417] Moffett, K. , & Sgro, T. (2016). School‐based CVE strategies. The Annals of the American Academy of Political and Social Science, 668(1), 145–164. 10.1177/0002716216672435

[cl21111-bib-0418] Mohtadi, H. (2017). Risk‐mitigating policies and adversarial behavior: Case of backlash. Risk Analysis, 37, 459–470. 10.1111/risa.12636 27279595

[cl21111-bib-0419] * Molenkamp, M. , & Wouterse, L. (2018). *Triple P: Coordination and collaboration between police, prison and probation services in dealing with violent extremist and terrorist offenders*. Paper presented at the RAN POL P&P Joint Event, Prague, Czech Republic.

[cl21111-bib-0420] Möller, K. , & Neuscheler, F. (2018). *Bericht über zentrale ergebnisse der evaluation der beratungsstelle hessen — Religiöse toleranz statt extremismus (Managementfassung)*. Berlin, Germany: Violence Prevention Network.

[cl21111-bib-0421] Monetti, M. (2018). *Boston components communications experiment report*. New York, NY: National Urban Security Technology Laboratory (NUSTL). Retrieved from https://www.dhs.gov/sites/default/files/publications/753_NUSTL_Boston-Components-Comms-OpExReport_1802.pdf

[cl21111-bib-0422] Moore, K. S. (Ed.). (2013). The most pressing issue of 2013 is…. *The Police Chief, 80*(2).

[cl21111-bib-0423] Morabito, M. S. (2010). Understanding community policing as an innovation: Patterns of adoption. Crime & Delinquency, 56(4), 564–587. 10.1177/0011128707311643

[cl21111-bib-0424] Morag, N. (2011). Comparative homeland security. Hoboken, NJ: John Wiley & Sons.

[cl21111-bib-0426] Moran, J. & Phythian, M. (Eds.). (2008). Intelligence, security and policing post‐9/11: The UK's response to the 'war on terror'. Basingstoke, UK: Palgrave Macmillan.

[cl21111-bib-0427] Morentin, B. , Callado, L. F. , & Idoyaga, M. I. (2008). A follow‐up study of allegations of ill‐treatment/torture in incommunicado detainees in Spain. Torture: Quarterly Journal on Rehabilitation of Torture Victims and Prevention of Torture, 18(2), 87–98. 19289885

[cl21111-bib-0428] Morris, M. , Eberhard, F. , Rivers, J. , & Watsula, M. (2010). *Deradicalization: A review of the literature with comparison to findings in the literatures on deganging and deprogramming* (Research Brief). Institute for Homeland Security Solutions.

[cl21111-bib-0429] Moss, S. (2012). *Do attempts to counter violent extremism merely exacerbate the problem?* Paper presented at the GTReC ARC Linkage Project on Radicalisation Conference 2012—Terrorism and counter‐terrorism in Australia and Indonesia: 10 years after Bali, Melbourne, VIC, Australia.

[cl21111-bib-0430] Mould, N. , Regens, J. L. , Jensen, C. J. , & Edger, D. N. (2014). Video surveillance and counterterrorism: The application of suspicious activity recognition in visual surveillance systems to counterterrorism. Journal of Policing, Intelligence and Counter Terrorism, 9(2), 10.1080/18335330.2014.940819. 151‐17.

[cl21111-bib-0431] Mucchielli, L. (2017). L'évolution des polices municipales en France: Une imitation des polices d'État vouée à I'échec? Deviance et Societe, 41(2), 239–271.

[cl21111-bib-0432] Muniesa, A. , & Rayna, C. (2002). De Tampere à Séville: bilan de la sécurité européenne [Special issue]. Cultures & Conflits, 45, 5–169. 10.4000/conflits.36

[cl21111-bib-0433] Murphy, J. J. (2007). *En une fraction de seconde: Une étude préliminaire sur le recours à la force par les policiers et la formation sur le recours à la force par les policiers au Canada [Betond a split‐second: An exploratory study of police use of force and use of force training in Canada]*. Ottawa, ON, Canada: Public Safety Canada.

[cl21111-bib-0434] Murphy, C. (2016). *Community engagement to counter radicalisation is a team effort*. Retrieved from https://www.aspistrategist.org.au/community-engagement-counter-radicalisation-team-effort/

[cl21111-bib-0435] Murphy, K. , Cherney, A. , & Teston, M. (2018). Promoting Muslims’ willingness to report terror threats to police: Testing competing theories of procedural justice. Justice Quarterly, 36(4), 594–619. 10.1080/07418825.2018.1437210

[cl21111-bib-0436] Murphy, K. , Cramer, R. J. , Waymire, K. A. , & Barkworth, J. (2018). Police bias, social identity, and minority groups: A social psychological understanding of cooperation with police. Justice Quarterly, 35(6), 1105–1130. 10.1080/07418825.2017.1357742

[cl21111-bib-0437] Murphy, G. R. , Plotkin, M. R. , Flynn, E. A. , Perlov, J. , Stafford, K. , & Stephens, D. W. (2003a). Protecting your community from terrorism: The strategies for local law enforcement series (1–5). Washington, DC: Community Oriented Policing Services U.S. Department of Justice.

[cl21111-bib-0438] Murphy, G. R. , Plotkin, M. R. , Flynn, E. A. , Perlov, J. , Stafford, K. , & Stephens, D. W. (2003b). Protecting your community from terrorism: The strategies for local law enforcement series (1–6). Washington, DC: Community Oriented Policing Services U.S. Department of Justice.

[cl21111-bib-0439] Murphy, K. , Madon, N. S. , & Cherney, A. (2018). Reporting threats of terrorism: Stigmatisation, procedural justice and policing Muslims in Australia. Policing and Society, 30, 361–377. 10.1080/10439463.2018.1551393

[cl21111-bib-0440] Murray, M. (2018). *Assessing the importance of CVE strategies in Ontario* (Master's thesis). Retrieved from https://ir.lib.uwo.ca/sociology_masrp/22

[cl21111-bib-0441] Namgung, H. (2013). *How do specialized units affect the outputs of police organizations? Investigating the effect of community policing units on community policing activities in local police departments* (Doctoral dissertation). Retrieved from ProQuest Dissertations and Theses Global database. (UMI No. 3605955).

[cl21111-bib-0442] National Consortium for the Study of Terrorism and Responses to Terrorism . (2012). *Law enforcement efforts to counter violent extremism: Lessons learned from past cases* (Research brief). Retrieved from https://www.dhs.gov/sites/default/files/publications/OPSR_TP_Countermeasures-Lessons-Learned-from-LE-Case-Studies_Sept2012-508_0.pdf

[cl21111-bib-0443] National Consortium for the Study of Terrorism and Responses to Terrorism . (2015). *Supporting a multidisciplinary approach to violent extremism: The integration of mental health in countering violent extremism (CVE) and what law enforcement needs to know*. Retrieved from https://www.dhs.gov/sites/default/files/publications/OPSR_TP_Cross-Training-Primer_Law-Enforcement_2015-508.pdf

[cl21111-bib-0444] National Institute of Justice . (2015). Radicalization and violent extremism: Lessons learned from Canada, the U.K., and the U.S. Washington, DC: National Institute of Justice.

[cl21111-bib-0445] National Institute of Justice . (2017a). *Research on domestic radicalization and terrorism*. Retrieved from https://nij.ojp.gov/topics/articles/research-domestic-radicalization-and-terrorism

[cl21111-bib-0446] National Institute of Justice . (2017b). *What can we learn from the similarities and differences between Lone Wolf Terrorists and Mass Murderers?* Retrieved from https://nij.ojp.gov/topics/articles/what-can-we-learn-similarities-and-differences-between-lone-wolf-terrorists-and

[cl21111-bib-0447] National Institute of Justice . (2018). *Research provides guidance on building effective counterterrorism programs*. Retrieved from https://nij.ojp.gov/topics/articles/research-provides-guidance-building-effective-counterterrorism-programs

[cl21111-bib-0448] Navarro, J. (2013). Hunting terrorists: A look at the psychopathology of terror. Springfield, IL: Charles C Thomas Publishing.

[cl21111-bib-0449] Neild, R. (2009). Ethnic profiling in the European Union: Pervasive, ineffective, and discriminatory. New York, NY: Open Society Institute.

[cl21111-bib-0450] Neumann, P. R. (2010). *Prisons and terrorism: Radicalisation and de‐radicalisation in 15 countries*. International Centre for the Study of Radicalisation and Political Violence. Retrieved from https://www.clingendael.org/sites/default/files/pdfs/Prisons-and-terrorism-15-countries.pdf

[cl21111-bib-0451] Nikoofal, M. E. , & Zhuang, J. (2015). On the value of exposure and secrecy of defense system: First‐mover advantage vs. robustness. European Journal of Operational Research, 246, 320–330. 10.1016/j.ejor.2015.04.043

[cl21111-bib-0452] Nix, J. , Wolfe, S. E. , & Campbell, B. A. (2018). Command‐level police officers’ perceptions of the “war on cops” and de‐policing. Justice Quarterly, 35(1), 33–54. 10.1080/07418825.2017.1338743

[cl21111-bib-0453] O'Callaghan, M. (2003). The thinning blue line. Bulletin With Newsweek, 121(6402), 24–38.

[cl21111-bib-0454] O'Connor, D. (2005). Closing the gap: A review of the fitness for purpose of the current structure of policing in England & Wales. London, UK: Home Office.

[cl21111-bib-0455] O'Toole, T. , Meer, N. , DeHanas, D. N. , Jones, S. H. , & Modood, T. (2016). Governing through prevent? Regulation and contested practice in State–Muslim engagement. Sociology, 50(1), 160–177. 10.1177/0038038514564437 26877558 PMC4735676

[cl21111-bib-0456] Office of Justice Programs . (2017). *Assessment of BJA's state and local anti‐terrorism training program*. Retrieved from https://nij.ojp.gov/topics/articles/assessment-bjas-state-and-local-anti-terrorism-training-program

[cl21111-bib-0457] Oleszkiewicz, S. , Granhag, P. A. , & Kleinman, S. M. (2017). Eliciting information from human sources: Training handlers in the Scharff technique. Legal & Criminological Psychology, 22(2), 400–419. 10.1111/lcrp.12108

[cl21111-bib-0458] Oliphant, R. (2017). *Protecting Canadians and their rights: A new road map for Canada's national security: Report of the Standing Committee on Public Safety and National Security (42nd Parliament, 1st Session)*. Ottawa, ON, Canada: House of Commons Canada.

[cl21111-bib-0459] Omer, S. B. , Barnett, D. J. , Castellano, C. , Wierzba, R. K. , Hiremath, G. S. , Balicer, R. D. , & Everly, G. S., Jr. (2007). Impact of homeland security alert level on calls to a law enforcement peer support hotline. International Journal of Emergency Mental Health, 9(4), 253–258. 18459529

[cl21111-bib-0460] Onyango, R. (2018). *Process evaluation of terrorism amnesty and reintegration program, and perceptions of the program within Kenya police* (Doctoral dissertation). Indexed in ProQuest Dissertations and Theses Global database. (ProQuest No. 10811560).

[cl21111-bib-0461] Ordi, G. H. , Miguel‐Tobal, J. J. , Vindel, A. C. , & Iruarrizaga, I. (2004). Efectos de la exposición a eventos traumáticos en personal de emergencias: Consecuencias psicopatológicas tras el atentado terrorista del 11‐M en Madrid. Ansiedad y Estrés, 10(2‐3), 207–217.

[cl21111-bib-0462] Organisation for the Security and Co‐operation in Europe (2014). *Preventing terrorism and countering violent extremism and radicalization that lead to terrorism: A community‐policing approach*. Vienna, Austria: The Organisation for the Security and Co‐operation in Europe.

[cl21111-bib-0463] O'Toole, T. , DeHanas, D. N. , & Modood, T. (2012). Balancing tolerance, security and Muslim engagement in the United Kingdom: The impact of the ‘Prevent’ agenda. Critical Studies on Terrorism, 5(3), 373–389. 10.1080/17539153.2012.725570

[cl21111-bib-0464] Ouellet, M. , Bouchard, M. , & Hart, M. (2017). Criminal collaboration and risk: The drivers of Al Qaeda's network structure before and after 9/11. Social Networks, 51, 171–177. 10.1016/j.socnet.2017.01.005

[cl21111-bib-0465] Özeren, S. , & Cinoğlu, H. (2010). Terörle mücadelede toplum desteklik polislik uygulamaları: Karşılaştırmalı analiz, Toplum Destekli Polislik: Toplum suç ve güvenlik. Ankara, Turkey: Adalet Yayınevi.

[cl21111-bib-0466] Ozgul, F. , Erdem, Z. , & Bowerman, C. (2009). Two models for semi‐supervised terrorist group detection. In N. Memon , J. D. Farley , D. L. Hicks & T. Rosenorn (Eds.), Mathematical methods in counterterrorism (pp. 229–249). Vienna, Austia: Springer.

[cl21111-bib-0467] Page Poma, F. R. (2015). *Contention and control: Violent protest policing in democratic Argentina* (Doctoral dissertation). Retrieved from ProQuest Dissertations and Theses database. (UMI No. 10013720).

[cl21111-bib-0468] Parent, R. B. , & Ellis, J. O. (2016). Countering radicalization in the community and in prison environments. Law Enforcement Executive Forum, 16(3), 50–63.

[cl21111-bib-0469] Parent, R. B. , & Ellis, J. O., III . (2011). *Countering radicalization of diaspora communities in Canada* (Working Paper Series No. 11‐12). Vancouver, BC, Canada: Centre of Excellence for Research on Immigration and Diversity.

[cl21111-bib-0470] Parkin, W. S. , Freilich, J. D. , Chermak, S. M. , & Gruenewald, J. (2016). *Criminal justice & military deaths at the hands of extremists*. National Consortium for the Study of Terrorism and Responses to Terrorism. Retrieved from https://www.start.umd.edu/pubs/START_CriminalJusticeMilitaryDeathsbyExtremists_BackgroundReport_Nov2016.pdf

[cl21111-bib-0471] Pastor, J. F. (2010). Terrorism and public safety policing: Implications for the Obama Presidency. Boca Raton, FL: CRC Press.

[cl21111-bib-0472] Pate, A. (2015). *Surveying the literature on counter‐terrorism, counter‐insurgency, and countering violent extremism: A summary report with a focus on Africa*. National Consortium for the Study of Terrorism and Responses to Terrorism. Retrieved from https://www.start.umd.edu/pubs/START_SMA-AFRICOM_LiteratureReview_Jan2015.pdf

[cl21111-bib-0473] Paterson, W. (2012). *The changing landscape of terrorism* (Public Lecture). Nathan, QLD, Australia: ARC Centre of Excellence in Policing and Security.

[cl21111-bib-0474] Peak, K. J. (Ed.). (2013). Encyclopedia of community policing and problem solving. Thousand Oaks, CA: Sage Publications.

[cl21111-bib-0475] Pedersen, M. J. B. , Gjerland, A. , Rund, B. R. , Ekeberg, Ø. , & Skogstad, L. (2016). Emergency preparedness and role clarity among rescue workers during the terror attacks in Norway July 22, 2011. PLoS One, 11(6), 1–12. 10.1371/journal.pone.0156536 PMC4900570 27280520

[cl21111-bib-0476] * Pelfrey, W. V., Jr. (2007). Local law enforcement terrorism prevention efforts: A state level case study. Journal of Criminal Justice, 35(3), 313–321. 10.1016/j.jcrimjus.2007.03.007

[cl21111-bib-0477] Peña, C. V. (2006). A smaller military to fight the war on terror. Orbis, 50(2), 289–306. 10.1016/j.orbis.2006.01.007

[cl21111-bib-0478] Perry, G. , & Hasisi, B. (2018). Closing the gap: Promoting suspect communities’ cooperation with airport security. Terrorism and Political Violence, 10.1080/09546553.2018.1442331

[cl21111-bib-0479] Peterson, A. (2012). Legitimacy and the Swedish Security Service's attempts to mobilize Muslim communities. International Journal of Criminology and Sociology, 1, 108–120.

[cl21111-bib-0480] Pettinger, T. (2017). De‐radicalization and counter‐radicalization: Valuable tools combating violent extremism, or harmful methods of subjugation? Journal for Deradicalization, 12, 1–59.

[cl21111-bib-0481] Phillips, C. , Tse, D. , Johnson, F. , & Mori, I. (2011). *Community cohesion and PREVENT: How have schools responded?* (Research Report DFE‐RR085). London, UK: Department for Education.

[cl21111-bib-0482] Piazza, J. A. (2015). Terrorist suspect religious identity and public support for harsh interrogation and detention practices. Political Psychology, 36(6), 667–690. 10.1111/pops.12190

[cl21111-bib-0483] Piazza, J. A. , & Piazza, S. (2017). Crime pays: Terrorist group engagement in crime and survival. Terrorism and Political Violence, 10, 1–23. 10.1080/09546553.2017.1397515

[cl21111-bib-0484] Pickering, S. , McCulloch, J. , & Wright‐Neville, D. (2008). Counter‐terrorism policing: Towards social cohesion. Crime, law, and social change, 50(1‐2), 91–109. 10.1007/s10611-008-9119-3

[cl21111-bib-0485] Pickering, S. , McCulloch, J. , & Wright‐Neville, D. (2008). Counter‐terrorism policing: Community, cohesion and security. New York, NY: Springer.

[cl21111-bib-0486] * Pickering, S. , Wright‐Neville, D. , McCulloch, J. , & Lentini, P. (2007). *Counter‐terrorism policing and culturally diverse communities: Final report 2007* (Linkage Project No. LP0454956). Australian Research Council Linkage Project. Retrieved from https://www.monash.edu/__data/assets/pdf_file/0011/1672805/counterterrorreport-07.pdf

[cl21111-bib-0487] Pilkington, H. (2018). *Violent extremism: How communities can help counter it*. Retrieved from https://theconversation.com/violent-extremism-how-communities-can-help-counter-it-100622

[cl21111-bib-0488] Pilkington, H. , & Acik, N. (2018). *Youth mobilisations of ‘suspect communities’*. Retrieved from http://www.promise.manchester.ac.uk/en/home-page/

[cl21111-bib-0489] Pisoiu, D. , & Ahmed, R. (2016). *Radicalisation research—Gap analysis*. RAN Centre of Excellence. Retrieved from https://ec.europa.eu/home-affairs/sites/homeaffairs/files/docs/pages/201612_radicalisation_research_gap_analysis_en.pdf

[cl21111-bib-0490] * Pisoiu, D. I. (2018). *Engaging with communities in P/CVE*. Paper presented at RAN Policy & Practice Event, Berlin, Germany.

[cl21111-bib-0491] Poiret, G. , & Beylier, P. A. (2016). The Mohawk reserve of Akwesasne between Canada and USA, smuggling area and weakness in the border security. Territoire en Mouvement, 29, 1–18.

[cl21111-bib-0492] Potter, M. A. , Sweeney, P. , Iuliano, A. D. , & Allswede, M. P. (2007). Performance indicators for response to selected infectious disease outbreaks: A review of the published record. Journal of Public Health Management and Practice, 13(5), 510–518. 17762697 10.1097/01.PHH.0000285205.40964.28

[cl21111-bib-0493] Poutvaara, P. , & Priks, M. (2006). *Hooliganism in the shadow of the 9/11 terrorist attack and the tsunami: Do police reduce group violence?* (CESifo Working Paper No. 1882). Retrieved from https://papers.ssrn.com/sol3/papers.cfm?abstract_id=956275

[cl21111-bib-0494] Poutvaara, P. , & Priks, M. (2009). The effect of police intelligence on group violence: Evidence from reassignments in Sweden. Journal of Public Economics, 93(3‐4), 403–411. 10.1016/j.jpubeco.2008.10.004

[cl21111-bib-0495] Power, N. , & Alison, L. (2016). Offence or defence? Approach and avoid goals in the multi‐agency emergency response to a simulated terrorism attack. Journal of Occupational and Organizational Psychology, 90, 51–76. 10.1111/joop.12159

[cl21111-bib-0496] Presman, D. , Chapman, R. , & Rosen, L. (2002). *Creative partnerships: Supporting youth, building communities*. Washington, DC: Office of Community Oriented Policing Services, U.S. Department of Justice.

[cl21111-bib-0497] Privy Counsellor Review Committee (2003). *Anti‐terrorism, Crime and Security Act 2001 review: Report*. London, UK: The Stationery Office.

[cl21111-bib-0498] Public Health and Law Enforcement Emergency Preparedness Workgroup (2008). *A framework for improving cross‐sector coordination for emergency preparedness and response: Action steps for public health, law enforcement, the judiciary, and corrections*. Washington, DC: Centers for Disease Control and Prevention, Bureau of Justice Assistance.

[cl21111-bib-0499] *Public Safety Canada (2013a). Building resilience against terrorism: Canada's counter‐terrorism strategy. Ottawa, ON, Canada: Public Safety Canada.

[cl21111-bib-0500] Public Safety Canada (2013b). *2013 Public report on the terrorist threat to Canada: Building a safe and resilient Canada*. Ottawa, ON, Canada: Public Safety Canada.

[cl21111-bib-0501] Public Safety Canada (2014). *2014 Public report on the terrorist threat to Canada*. Ottawa, ON, Canada: Public Safety Canada.

[cl21111-bib-0502] Public Safety Canada (2016a). *2016 Public report on the terrorist threat to Canada*. Ottawa, ON, Canada: Public Safety Canada.

[cl21111-bib-0503] Public Safety Canada (2016b). *Our security, our rights: National security green paper, 2016*. Ottawa, ON, Canada: Government of Canada.

[cl21111-bib-0504] Public Safety Canada (2017). *2017 Public report on the terrorist threat to Canada*. Ottawa, ON, Canada: Public Safety Canada.

[cl21111-bib-0505] *Public Safety Canada (2018a). *National Strategy on countering radicalization to violence*. Ottawa, ON, Canada: Public Safety Canada.

[cl21111-bib-0506] Public Safety Canada (2018b). *Strengthening Canada's counter‐proliferation framework*. Ottawa, ON, Canada: Public Safety Canada.

[cl21111-bib-0507] Public Safety Canada. *Federal terrorism response plan: Domestic concept of operations*. Ottawa, ON, Canada: Government of Canada.

[cl21111-bib-0508] Quick, D. , & Choo, K.‐K. R. (2017). Big forensic data management in heterogeneous distributed systems: quick analysis of multimedia forensic data. Software: Practice and Experience, 47, 1095–1109. 10.1002/spe.2429

[cl21111-bib-0509] Rabbit, E. P. (2009). Preparing for the worst: An analysis of homeland security collaboration among state government agencies (Master's thesis). Retrieved from ProQuest Dissertations and Theses Global database. (UMI No. 1462366).

[cl21111-bib-0510] Radicalisation Action Network (2014). Preventing radicalisation to terrorism and violent extremism: Strengthening the EU's response. Brussels, Belgium: European Commission. Retrieved from https://ec.europa.eu/home-affairs/sites/homeaffairs/files/e-library/documents/policies/crisis-and-terrorism/radicalisation/docs/communication_on_preventing_radicalisation_and_violence_promoting_extremism_201301_en.pdf

[cl21111-bib-0511] Radicalisation Awareness Network . (2016a). *Successful and effective engaging with communities*. Paper presented at RAN POL, Oslo.

[cl21111-bib-0512] Radicalisation Awareness Network . (2016b). *RAN P&P Multiagency cooperation around radicalised offenders*. Paper presented at RAN POL, Stockholm.

[cl21111-bib-0513] Radicalisation Awareness Network . (2017). *Strengthening community resilience to polarisation and radicalisation*. Paper presented at RAN YF&C, London.

[cl21111-bib-0514] Radicalisation Awareness Network (2018). *Preventing radicalisation to terrorism and violent extremism: Approaches and practices*. Luxembourg: The Publications Office of the European Union.

[cl21111-bib-0515] Radnayeva, E. L. , & Boskholov, S. S. (2016). On Russian national security through the lenses of the problems of contemporary legal education and criminology as a research and academic discipline. Russian Journal of Criminology, 10, 608–617.

[cl21111-bib-0516] Ramirez, D. A. (2012). *Developing partnerships between law enforcement and American Muslim*. Arab, and Sikh Communities: A promising practices guide. Retrieved from https://ssrn.com/abstract=1998442

[cl21111-bib-0517] RAN Centre of Excellence . (2018). *A Nimble (NMBL) approach to youth engagement in P/CVE*. Retrieved from https://ec.europa.eu/home-affairs/sites/homeaffairs/files/what-we-do/networks/radicalisation_awareness_network/about-ran/ran-young/docs/nimble_approach_to_youth_engagement_in_pcve_2018_en.pdf

[cl21111-bib-0518] Randol, B. M. (2012). The organizational correlates of terrorism response preparedness in local police departments. Criminal Justice Policy Review, 23(3), 304–326. 10.1177/0887403411400729

[cl21111-bib-0519] * Randol, B. M. (2013). An exploratory analysis of terrorism prevention and response preparedness efforts in municipal police departments in the United States: Which agencies participate in terrorism prevention and why? The Police Journal, 86, 158–181. 10.1350/pojo.2013.86.2.618

[cl21111-bib-0520] Ratcliffe, J. (2008). Intelligence‐led policing. Cullompton, UK: Willan Publishing.

[cl21111-bib-0521] Rausch, S. , & LaFree, G. (2007). The growing importance of criminology in the study of terrorism. The Criminologist, 32(6), 1–3.

[cl21111-bib-0522] Reding, A. , Van Gorp, A. , Robertsin, K. , Wakczak, A. , Giacomantonio, C. , & Hoorens, S. (2014). *Handling ethical problems in counterterrorism: An inventory of methods to support ethical decisionmaking*. Santa Monica, CA: RAND Corporation. Retrieved from https://www.rand.org/pubs/research_reports/RR251.html

[cl21111-bib-0523] Redo, S. M. (2012). *Blue criminology: The power of United Nations ideas to counter crime globally*. (HEUNI Publication Series No. 72). Helsinki, Finland: European Institute for Crime Prevention and Control.

[cl21111-bib-0524] Reed, E. , Devost, M. G. , & Pollard, N. (2005). *Utilizing terrorism early warning groups to meet the national preparedness goal*. Terrorism Research Centre, Retrieved from http://www.terrorism.org/wp-content/uploads/2015/12/TEW_and_National_Preparedness_Goal-TRC-May2005.pdf

[cl21111-bib-0525] Regehr, C. , & Bober, T. (2005). In the line of fire: Trauma in the emergency services . Oxford Bibliographies Online. 10.1093/acprof:oso/9780195165029.001.0001

[cl21111-bib-0526] Regens, J. L. , Mould, N. A. , Jensen, C. J., III , & Edger, D. N. (2016). Terrorism‐centric behavior recognition and adversarial threat forecasting. International Journal of Intelligence & Counterintelligence, 29(2), 328–340. 10.1080/08850607.2015.108331

[cl21111-bib-0527] Regens, J. L. , Mould, N. , Jensen, C. J. , Graves, M. A. , & Edger, D. N. (2015). Probabilistic graphical modeling of terrorism threat recognition using Bayesian networks and Monte Carlo simulation. Journal of Cognitive Engineering and Decision Making, 9(4), 295–311. 10.1177/1555343415592730

[cl21111-bib-0528] Regens, J. L. , Mould, N. , Jensen, C. J., III , & Graves, M. A. (2016). Terrorism‐centric behaviors and adversarial threat awareness. Social Science Quarterly, 97(3), 791–806. 10.1111/ssqu.12233

[cl21111-bib-0529] Regens, J. L. , Mould, N. , Jensen, C. J., III , Edger, D. N. , Cid, D. , & Graves, M. (2017). Effect of intelligence collection training on suspicious activity recognition by front line police officers. Security Journal, 30(3), 951–962. 10.1057/sj.2015.10

[cl21111-bib-0530] Rehman, F. U. (2015). The spatial analysis of terrorism in Pakistan. Asian Journal of Law and Economics, 6(2), 125–165. 10.1515/ajle-2015-0009

[cl21111-bib-0531] Restrepo, E. M. , Sánchez, F. , & Cuéllar, M. M. (2006). Impunity or punishment? An analysis of criminal investigation into kidnapping, terrorism and embezzlement in Colombia. Global Crime, 7(2), 176–199. 10.1080/17440570601014446

[cl21111-bib-0532] Reynolds, L. (2019). *Maintaining vigilance to combat terrorism*. Retrieved from https://nij.ojp.gov/topics/articles/maintaining-vigilance-combat-terrorism

[cl21111-bib-0533] Richman, D. (2006). The past, present, and future of violent crime federalism. Crime and Justice, 34(1), 377–439.

[cl21111-bib-0534] Ritzmann, A. (2017). *RAN guidelines for effective alternative and counter‐narrative campaigns (GAMMMA+)*. RAN Centre of Excellence. Retrieved from https://ec.europa.eu/home-affairs/sites/homeaffairs/files/what-we-do/networks/radicalisation_awareness_network/about-ran/ran-c-and-n/docs/ran_cn_guidelines_effective_alternative_counter_narrative_campaigns_31_12_2017_en.pdf

[cl21111-bib-0535] Robinson Salazar, P. (2011). Plutocracy, the new right and securitization of natural strategic resources in Latin America: A necessary reflection. Opcion, 27(64), 13–45.

[cl21111-bib-0536] Robinson, L. , Helmus, T. C. , Cohen, R. S. , Nader, A. , Radin, A. , Magnusin, M. , & Migacheva, K. (2018). *Modern political warfare: Current practices and possible responses*. Santa Monica, CA: RAND Corporation.

[cl21111-bib-0537] * Roberts, A. , Roberts, J. M., Jr. , & Liedka, R. V. (2012). Elements of terrorism preparedness in local police agencies, 2003‐2007: Impact of vulnerability, organizational characteristics, and contagion in the Post‐9/11 era. Crime and Delinquency, 58(5), 720–747. 10.1177/0011128712452960

[cl21111-bib-0538] Romaniuk, P. (2015). *Does CVE work? Lessons learned from the global effort to counter violent extremism*. Center on Global Counterterrorism Cooperation. Retrieved from https://www.globalcenter.org/wp-content/uploads/2015/09/Does-CVE-Work_2015.pdf

[cl21111-bib-0539] Romaniuk, P. , & Durner, T. (2018). The politics of preventing violent extremism: The case of Uganda. Conflict, Security and Development, 18(2), 159–179. 10.1080/14678802.2018.1447863

[cl21111-bib-0540] Romaniuk, P. , & Fink, N. C. (2012). *From input to impact: Evaluating terrorism prevention programs*. Center on Global Counterterrorism Cooperation. Retrieved from https://www.files.ethz.ch/isn/153962/CGCC_EvaluatingTerrorismPrevention.pdf

[cl21111-bib-0541] Rosand, E. (2007). *Global terrorism: Multilateral responses to an extraordinary threat*. International Peace Academy. Retrieved from https://www.globalcenter.org/wp-content/uploads/2007/04/coping_with_crisis.pdf

[cl21111-bib-0542] Rosand, E. (2009). *The UN office on drugs and crime's terrorism prevention branch: Strengths and challenges ahead*. Center on Global Counterterrorism Cooperation. Retrieved from https://www.globalcenter.org/wp-content/uploads/2009/07/rosand_policybrief_093.pdf

[cl21111-bib-0543] Rosand, E. , Fink, N. C. , & Ipe, J. (2009). Countering terrorism in South Asia: Strengthening multilateral engagement. International Peace Institute. Retrieved from https://www.globalcenter.org/wp-content/uploads/2009/05/south_asia.pdf

[cl21111-bib-0544] Rosand, E. , Ipe, J. , & Millar, A. (2007). *Implementing the UN Global Counter‐Terrorism Strategy in Southern Africa*. Center on Global Counterterrorism Cooperation. Retrieved from https://www.globalcenter.org/wp-content/uploads/2007/11/southern_africa.pdf

[cl21111-bib-0545] Rosand, E. , Millar, A. , & Ipe, J. (2008). *Implementing the UN Global Counter‐Terrorism Strategy in the Latin America and Caribbean region*. Center on Global Counterterrorism Cooperation, Retrieved from https://www.globalcenter.org/wp-content/uploads/2008/09/latin_america.pdf

[cl21111-bib-0546] Rosand, E. , Millar, A. , & Ipe, J. (2009). Enhancing counterterrorism cooperation in Eastern. Africa. African Security Review, 18(2), 93–106.

[cl21111-bib-0547] Rosand, E. , Millar, A. , & Ipe, J. (2010). *Implementing the UN Global Counter‐Terrorism Strategy in East Africa*. London, UK: Center on Global Counterterrorism Cooperation.

[cl21111-bib-0548] Rosand, E. , Millar, A. , Ipe, J. , & Healey, M. (2008). *The UN Global Counter‐Terrorism Strategy and regional and subregional bodies: Strengthening a critical partnership*. Center on Global Counterterrorism Cooperation. Retrieved from https://www.globalcenter.org/wp-content/uploads/2008/10/strengthening_a_critical_partnership.pdf

[cl21111-bib-0549] Royal Canadian Mounted Police (2015). *Terrorism prevention program*. Ottawa, ON, Canada: Public Safety Canada.

[cl21111-bib-0550] RTI Interational . (2018). *Countering violent extremism—The application of risk assessment tools in the criminal justice and rehabilitation process: Literature review*. Retrieved from https://www.dhs.gov/sites/default/files/publications/OPSR_TP_CVE-Application-Risk-Assessment-Tools-Criminal-Rehab-Process_2018Feb-508.pdf

[cl21111-bib-0551] RTI International . (2017). *Countering violent extremism—The use of assessment tools for measuring violence risk: Literature review*. Retrieved from https://www.dhs.gov/sites/default/files/publications/OPSR_TP_CVE-Use-Assessment-Tools-Measuring-Violence-Risk_Literature-Review_March2017-508.pdf

[cl21111-bib-0552] RTI International . (2017). *Countering violent extremism (CVE)—Developing a research roadmap*. Retrieved from https://www.dhs.gov/sites/default/files/publications/861_OPSR_TP_CVE-Developing-Research-Roadmap_Oct2017.pdf

[cl21111-bib-0553] RTI International . (2017). *International expert engagement and analysis of countering violent extremism (CVE) evaluations: Final report*. Retrieved from https://www.dhs.gov/sites/default/files/publications/860_OPRS_TP_Intl-Expert-Engagement-Analysis_CVE_Evaluators_2017Nov-508.pdf

[cl21111-bib-0554] Russell, J. (2018). *Developing counter‐ and alternative narratives together with local communities*. RAN Center of Excellence. Retrieved from https://ec.europa.eu/home-affairs/sites/homeaffairs/files/what-we-do/networks/radicalisation_awareness_network/about-ran/ran-c-and-n/docs/developing_counter_and_alternative_narratives_together_with_local_communities_en.pdf

[cl21111-bib-0555] Safer‐Lichtenstein, A. (2015). *Incorporating ideas of displacement and diffusion of benefits into evaluations of counterterrorism policy* (Doctoral dissertation). Retrieved from ProQuest Dissertations and Theses Global database. (UMI No. 1592442).

[cl21111-bib-0556] Sakiyama, M. (2018). *The balance between privacy and safety in police UAV use: The power of threat and its effect on people's receptivity* (Doctoral dissertation). Retrieved from ProQuest Dissertations and Theses Global database. (UMI No. 10688579).

[cl21111-bib-0557] Samra, K. M. (2018). *Multi‐agency working and preventing violent extremism I*. Paper presented at RAM Centre of Excellence, Ireland.

[cl21111-bib-0558] Sanchez‐Cuenca, I. (2009). Analyzing temporal variation in the lethality of eta. Revista Internacional de Sociologia, 67(3), 609–629. 10.3989/ris.2008.03.24

[cl21111-bib-0559] Sang, P. J. (2017). 국가재난 관리에 있어 경찰정보의 활용방안에 관한 연구 [A study on application for police information in national disaster management]. Journal of Korean Public Police and Security Studies, 14(3), 243–272.

[cl21111-bib-0560] Saraiva, E. R. A. , & Coutinho, M. P. L. (2012). Meios de comunicação impressos, Representações sociais e violência contra idosos [Print media, Social representations and violence against the elderly]. Psicologia em Estudo, 17(2), 205–214.

[cl21111-bib-0561] Schafer, J. A. , Burruss, G. W., Jr , & Giblin, M. J. (2009). Measuring homeland security innovation in small municipal agencies: Policing in a Post—9/11. World. Police Quarterly, 12(3), 263–288. 10.1177/1098611109339891

[cl21111-bib-0562] Schanzer, D. H. (2012). *The way forward on combating Al‐Qa'ida‐inspired violent extremism in the United States: Suggestions for the next administration*. Institute for Social Policy and Understanding, the Duke Islamic Studies Center, ISALMiCommentary, and the Triangle Center on Terrorism and Homeland Security, Retrieved from https://fds.duke.edu/db/attachment/2343

[cl21111-bib-0563] Schanzer, D. H. , & Eyerman, J. (2009). *Improving strategic risk management at the department of homeland security*. IBM Center for The Business of Government. Retrieved from https://www.mercatus.org/system/files/Applying_Strategic_Risk_Management_to_Allocating_Resources_for_Homeland_Security.pdf

[cl21111-bib-0564] Schanzer, D. , & Eyerman, J. (2016). *United States Attorneys’ community outreach and engagement efforts to counter violent extremism: Results from a nationwide survey*. Triangle Center on Terrorism and Homeland Security, Retrieved from https://dukespace.lib.duke.edu/dspace/handle/10161/15949

[cl21111-bib-0565] Schanzer, D. , Kurtzman, C. , Toliver, J. , & Miller, E. (2016). The challenge and promise of using community policing strategies to prevent violent extremism. Police Chief, 83(7), 14.

[cl21111-bib-0566] Schanzer, D. , Kurzman, C. , & Moosa, E. (2010). *Anti‐terror lessons of Muslim‐Americans*. Durham, NC: Sanford School of Public Policy. Retrieved from https://fds.duke.edu/db/attachment/1255

[cl21111-bib-0567] Schanzer, D. , Kurzman, C. , Toliver, J. , & Miller, E. (2016a). *The challenge and promise of using community policing strategies to prevent violent extremism: A call for community partnerships with law enforcement to enhance public safety*. Washington, DC: National Institute of Justice.

[cl21111-bib-0568] * Schanzer, D. , Kurzman, C. , Toliver, J. , & Miller, E. (2016b). *The challenge and promise of using community policing strategies to prevent violent extremism*. Triangle Center on Terrorism and Homeland Security. Retrieved from https://sites.duke.edu/tcths/2016/01/14/triangle-center-report-recommends-reforms-to-policing-practices-to-prevent-violent-extremism/

[cl21111-bib-0569] Scheider, M. C. , & Chapman, R. (2003). Community policing and terrorism. Journal of Homeland Security, 4–8.

[cl21111-bib-0570] Scherrer, A. (2009). G8 against transnational organized crime. New York, NY: Routledge.

[cl21111-bib-0571] Schmidt, A. , & Sikkink, K. (2018). Partners in crime: An empirical evaluation of the CIA rendition, detention, and interrogation program. Perspectives on Politics, 16(4), 1014–1033. 10.1017/S1537592717004224

[cl21111-bib-0572] Schneider, S. , & Hurst, C. (2008). Obstacles to an integrated, joint forces approach to organized crime enforcement: A Canadian case study. Policing, 31(3), 359–379. 10.1108/13639510810895759

[cl21111-bib-0573] Schuurman, B. , & Bakker, E. (2016). Reintegrating jihadist extremists: Evaluating a Dutch initiative, 2013–2014. Behavioral Sciences of Terrorism and Political Aggression, 8(1), 66–85. 10.1080/19434472.2015.1100648

[cl21111-bib-0574] Schuurman, B. , Harris‐Hogan, S. , Zammit, A. , & Lentini, P. (2014). Operation Pendennis: A case study of an Australian terrorist plot. Perspectives on Terrorism, 8(4), 91–99.

[cl21111-bib-0575] * Schwartz, M. (2015). *Policing and (in)security in fragile and conflict‐affected settings. Global Center on Cooperative Security*. Retrieved from https://www.globalcenter.org/wp-content/uploads/2015/05/policing-and-in-security-in-fragile-and-conflict-affected-settings.pdf

[cl21111-bib-0576] Schwartz, M. , Millar, A. , Kessels, E. , Lefas, M. , Nozawa, J. , & Rector, L. (2015). *Strengthening the case: Good criminal justice practices to counter terrorism*. Goshen, IN: Global Center on Cooperative Security.

[cl21111-bib-0577] Schwarzer, R. , Bowler, R. M. , & Cone, J. E. (2014). Social integration buffers stress in New York police after the 9/11 terrorist attack. Anxiety, Stress & Coping, 27(1), 18–26. 10.1080/10615806.2013.806652 23768128

[cl21111-bib-0578] Scott, E. D. Jr. (2006). *Factors influencing user‐level success in police information sharing: An examination of Florida's FINDER system* (Doctoral dissertation). Retrieved from ProQuest Dissertations and Theses Global database (UMI No. 3242470).

[cl21111-bib-0579] Searle, G. M. (2017). *Impact assessment in special warfare* (Master's thesis). Retrieved from http://hdl.handle.net/10945/56802

[cl21111-bib-0580] Secretariat . (2013). *Ways and means of enhancing the effectiveness of international cooperation in countering criminal and terrorist threats and challenges to the tourism sector, including by means of public‐private partnerships (Document No. E/CN.15/2013/19)*. Report of the Secretariat on the 22nd Session of the Commission on Crime Prevention and Criminal Justice, Vienna, Austria.

[cl21111-bib-0581] Sécurité Publique Canada . (2010). *Évaluation de 2009‐2010 du programme des services de police des Premières nations: Direction générale de l'évaluation*. Retrieved from https://www.securitepublique.gc.ca/lbrr/archives/cn27051-fra.pdf

[cl21111-bib-0582] Sécurité Publique Canada (2011). *Plan d'action en réponse à la Commission d'enquête sur l'affaire Air India: Rapport d'état des progress*. Sécurité Publique Canada. Retrieved from http://www.securitepublique.gc.ca/lbrr/archives/cn27672-fra.pdf

[cl21111-bib-0583] Sécurité Publique Canada . (2013). *Évaluations des programmes de la police frontalière*. Retrieved from https://www.securitepublique.gc.ca/lbrr/archives/cn63303282-fra.pdf

[cl21111-bib-0584] Sécurité Publique Canada . (2015). *Établir la portée du crime organisé*. Retrieved from https://www.publicsafety.gc.ca/lbrr/archives/cn38998-fra.pdf

[cl21111-bib-0585] Sehee, C. (2015). 경찰 위기협상 발전 방향에 관한 연구 ‐ 경찰 기관의 교육 발전 방안을 중심으로 [A study on the development direction of crisis negotiations of the police ‐ with focus on the police department education development plan]. 한국경호경비학회지, 45, 161–189.

[cl21111-bib-0586] Sevinç, A. (2013). *Terörle mücadelede toplum destekli polislik (TDP) uygulamalarinin yeri: TDP faaliyeti yürüten polislerin algisi [Community policing in counterterrorism: Perspectives of practitioners practicing community policing activities]* (Doctoral dissertation).

[cl21111-bib-0587] Sheikh, S. , Sarwar, S. , & King, E. (2012). *Evaluation of the Muslim Council of Wales’ Prevent work* (Research Summary No. 23). Cardiff, UK: Office for Public Management, Welsh Government.

[cl21111-bib-0588] Shetret, L. , Schwartz, M. , & Cotter, D. (2013). *Mapping perceptions of violent extremism: Pilot study of community attitudes in Kenya and Somaliland*. Goshen, IN: Centre on Global Counterterrorism Cooperation. Retrieved from https://www.globalcenter.org/wp-content/uploads/2013/01/Jan2013_MPVE_PilotStudy.pdf

[cl21111-bib-0589] Shields, C. A. , Smith, B. L. , & Damphouse, K. R. (2015). Prosecuting terrorism: Challenges in the post ‐9/11 world. In M. Deflem (Ed.), Terrorism and counterterrorism today (pp. 175–195). Bingley, UK: Emerald Group Publishing.

[cl21111-bib-0590] Shields, C. A. , Smith, B. L. , & Damphouse, K. R. (2016). Prosecuting terrorism post‐9/11: Impact of policy changes on case outcomes. In G. LaFree & J. D. Freilich (Eds.), The handbook of the criminology of terrorism (pp. 495–507). Hoboken, NJ: Wiley.

[cl21111-bib-0591] Šikman, M. , & Ivetić, S. (2011). Terrorism in Bosnia and Herzegovina — Current state and suppression measures. International Security, 3(2), 31–44.

[cl21111-bib-0592] Silber, M. D. , & Bhatt, A. (2007). Radicalization in the West: The homegrown threat. New York, NY: New York City Police Department.

[cl21111-bib-0593] Silk, D. P. (2012). The complexity of police‐Muslim community relations in the shadow of 9/11. Arches Quarterly, 5(9), 73–82.

[cl21111-bib-0594] Silk, P. D. , Spalek, B. & O'Rawe, M. (Eds.). (2013). Preventing ideological violence communities, police and case atudies of “success”. New York, NY: Palgrave Macmillan.

[cl21111-bib-0595] Silke, A. (Ed.), 2006). Terrorists, victims and society: Psychological perspectives on terrorism and its consequences. Chichester, UK: John Wiley & Sons Ltd.

[cl21111-bib-0596] Simone, J., Jr , Freilich, J. D. , & Chermak, S. M. (2008). *Surveying state police agencies about domestic terrorism and far‐right extremists*. National Consortium for the Study of Terrorism and Responses to Terrorism. Retrieved from http://www.start.umd.edu/start/publications/research_briefs/20080221_State_Agency_Survey.pdf

[cl21111-bib-0597] Sisto, F. , & Teufel, H., III (2008). *Privacy impact assessment for the law enforcement information data base (LEIDB)/Pathfinder*. U.S. Department of Homeland Security. Retrieved from https://www.dhs.gov/sites/default/files/publications/privacy_pia_004-uscg-leidbpathfinder-2008.pdf

[cl21111-bib-0598] Skillicorn, D. B. (2008). Knowledge discovery for counterterrorism and law enforcement. Boca Raton, FL: CRC Press.

[cl21111-bib-0599] Skogstad, L. , Heir, T. , Hauff, E. , & Ekeberg, Ø. (2016). Post‐traumatic stress among rescue workers after terror attacks in Norway. Occupational Medicine, 66(7), 528–535. 10.1093/occmed/kqw063 27325417

[cl21111-bib-0600] Slensky, K. A. , Drobatz, K. J. , Downend, A. B. , & Otto, C. M. (2004). Deployment morbidity among search‐and‐rescue dogs used after the September 11, 2001, terrorist attacks. Journal of the American Veterinary Medical Association, 225(6), 868–873. 15485045 10.2460/javma.2004.225.868

[cl21111-bib-0601] Sloan, B. , & Cockayne, J. (2011). *Terrorism, crime, and conflict: Exploiting the differences among transnational threats? (Policy brief)*. Center on Global Counterterrorism Cooperation. Retrieved from https://www.globalcenter.org/wp-content/uploads/2011/02/BS_policybrief_117.pdf

[cl21111-bib-0602] Smith, A. (2015). Telling stories: Preventing violent extremism through community engagement. The Police Chief, 82(2), 40–41.

[cl21111-bib-0603] Smith, B. L. , Shields, C. , & Damphousse, K. R. (2011). *Patterns of intervention in federal terrorism cases*. College Park, MD: National Consortium for the Study of Terrorism and Responses to Terrorism. Retrieved from https://www.dhs.gov/sites/default/files/publications/OPSR_TP_Countermeasures-Patterns-Intervention-Federal-Terrorism-Cases_Aug2011-508.pdf

[cl21111-bib-0604] Smith, C. C. (2007). *Conflict, crisis, and accountability: Racial profiling and law enforcement in Canada*. Ottawa, ON, Canada: Canadian Centre for Policy Alternatives.

[cl21111-bib-0605] Smith, J. (2014). A law enforcement and security officers’ guide to responding to bomb threats. Springfield, IL: Charles C Thomas Publisher, Ltd.

[cl21111-bib-0606] Soares, G. A. D. (2005). Political covariates of violent deaths. Opinião pública, 4(1), 192–212.

[cl21111-bib-0607] Solicitor General Canada . (2002). *Annual report on the use of arrest without warrant pursuant to the anti‐terrorism act*. Retrieved from https://www.publicsafety.gc.ca/cnt/rsrcs/lbrr/ctlg/dtls-en.aspx?d=PS&i=42855086

[cl21111-bib-0608] Sondergaard, S. , Paoli, G. P. , Cox, K. , Warnes, R. , Clutterbuck, L. , Bellanova, R. , … Pilner, J. (2015). TACTICS: Policy and strategic impacts, implications and recommendations. Santa Monica, CA: RAND Corporation. Retrieved from https://www.rand.org/pubs/research_reports/RR1287.html

[cl21111-bib-0609] Soria, V. (2011). *The new PREVENT strategy: Establishing realistic expectations*. Retrieved from https://rusi.org/commentary/new-prevent-strategy-establishing-realistic-expectations

[cl21111-bib-0610] Sormani, R. , Soldatoa, J. , Vassilaras, S. , Kioumourtzis, G. , Leventakis, G. , Giordani, I. , & Tisato, F. (2016). A serious game empowering the prediction of potential terrorist actions. Journal of Policing, Intelligence and Counter Terrorism, 11(1), 30–48. 10.1080/18335330.2016.1161222

[cl21111-bib-0611] Sorochinski, M. , Hartwig, M. , Osborne, J. , Wilkins, E. , Marsh, J. , Kazakov, D. , & Granhag, P. A. (2014). Interviewing to detect deception: When to disclose the evidence? Journal of Police and Criminal Psychology, 29(2), 87–94. 10.1007/s11896-013-912

[cl21111-bib-0612] Spalek, B. (2013). Terror crime prevention with communities. London, UK: Bloomsbury Academic.

[cl21111-bib-0613] Spalek, B. , & MacDonald, L. Z. (2012). Counter‐terrorism: Police and community engagement in Britain and the US. Arches Quarterly, 5(9), 20–39.

[cl21111-bib-0614] Speckhard, A. (2017). *Possibilities of peace‐building in Iraq: Questions of deradicalization and reintegration amidst sectarian conflicts*. Retrieved from https://www.huffpost.com/entry/possibilities-of-peacebui_b_14191234

[cl21111-bib-0615] Sphar, L. L. , Ederheimer, J. , & Bilson, D. (2007). Patrol‐level response to a suicide bomb threat: Guidelines for consideration. Washington, DC: Police Executive Research Forum.

[cl21111-bib-0616] Stageman, D. L. (2018). *Local immigration enforcement entrepreneurship in the punishment marketplace* (Doctoral dissertation). Retrieved from ProQuest Dissertations and Theses Global database. (ProQuest No. 10250085).

[cl21111-bib-0617] Standing Senate Committee on National Security and Defence . (2015). *Countering the terrorist threat in Canada: An interim report*. Retrieved from https://www.publicsafety.gc.ca/lbrr/archives/cn000043947279-eng.pdf

[cl21111-bib-0618] Stevens, T. , & Neumann, P. R. (2009). *Countering online radicalisation: A strategy for action*. London, UK: International Centre for the Study of Radicalisation and Political Violence (ICSR).

[cl21111-bib-0619] Stevenson, J. (2015). *Statistical analysis of event data concerning Boko Haram in Nigeria (2009–2013)*. National Consortium for the Study of Terrorism and Responses to Terrorism. Retrieved from https://www.start.umd.edu/pubs/START_SMA-AFRICOM_StatisticalAnalysis_Jan2015.pdf

[cl21111-bib-0620] Stewart, C. M. (2017). *Countering violent extremism policy in the United States: Are CVE programs in America effectively mitigating the threat of homegrown violent extremism* (Master's thesis). Retrieved from https://calhoun.nps.edu/bitstream/handle/10945/56816/17Dec_Stewart_Craig.pdf?sequence=1&isAllowed=y

[cl21111-bib-0621] Stewart, M. G. , & Mueller, J. (2013). *Cost‐benefit analysis of Australian Federal Police counter‐terrorism operations at Australian airports* (Working Paper October, Issue 2). Brisbane, QLD, Australia: ARC Centre of Excellence in Policing and Security.

[cl21111-bib-0623] Stewart, M. G. , & Mueller, J. (2014). A risk and cost‐benefit analysis of police counter‐terrorism operations at Australian airports. Journal of Policing, Intelligence and Counter Terrorism, 9(2), 98–116. 10.1080/18335330.2014.940816

[cl21111-bib-0624] Stewart, M. G. , & Mueller, J. (2018). Risk and economic assessment of US aviation security for passenger‐borne bomb attacks. Journal of Transportation Security, 11(3‐4), 117–136. 10.1007/s12198-018-0196-y

[cl21111-bib-0625] van Stolk, C. , Ling, T. , Reding, A. , & Bassford, M. (2011). Monitoring and evaluation in stabilisation interventions: Reviewing the state of the art and suggesting ways forward. Cambridge, UK: RAND Europe.

[cl21111-bib-0626] Straub, F. (2020). *The importance of community policing in preventing terrorism*. Retrieved from https://nij.ojp.gov/topics/articles/maintaining-vigilance-combat-terrorism

[cl21111-bib-0627] Striegher, J.‐L. (2013). Deradicalisation of Terrorists. Salus Journal, 1(1), 19–40.

[cl21111-bib-0628] Strom, K. , Hollywood, J. , Pope, M. , Weintraub, G. , Daye, C. , & Gemeinhardt, D. (2010). *Building on clues: Examining successes and failures in detecting US terrorist plots, 1999–2009*. Institute for Homeland Security Solutions, Retrieved from https://pdfs.semanticscholar.org/4b95/804280d3f92c1450426f22c597a095fa19f7.pdf

[cl21111-bib-0629] Sukhodolov, A. P. , Lebedev, A. V. , Toropov, B. A. , Babkin, A. A. , & Spasennikov, B. A. (2018). Mathematical methods in law enforcement: Counteracting extremism on social media. Russian Journal of Criminology, 12(4), 468–475. 10.17150/2500-4255.2018.12(4).468-47

[cl21111-bib-0630] Sullivan, C. M. (2016). Undermining resistance: Mobilization, repression, and the enforcement of political order. Journal of Conflict Resolution, 60(7), 1163–1190. 10.1177/0022002714567951

[cl21111-bib-0631] Swedish Agency for Youth and Civil Society . (2018). *Crack the code!—A guide on how public stakeholders and civil society can work together to prevent violent extremism*. Retrieved from https://www.mucf.se/sites/default/files/publikationer_uploads/crack_the_code.pdf

[cl21111-bib-0632] Talucci, V. , & Nancy, K. (2003). *Volunteers in Police Service*. pp. 1–11.

[cl21111-bib-0633] Taylor, S. C. , Torphy, D. J. & Das, D. K. (Eds.). (2013). Policing global movement: Tourism, migration, human trafficking, and terrorism. Boca Raton, FL: CRC Press.

[cl21111-bib-0634] Tehrani, N. , Rainbird, C. , & Dunne, B. (2011). Supporting the police following the 7/7 London terrorist bombs—An organisational approach. In N. Tehrani (Ed.), Managing trauma in the workplace: Supporting workers and organisations (pp. 191–203). Abingdon, UK: Routledge.

[cl21111-bib-0635] Tepe, F. (2009). *Leadership characteristics among command, middle, and line level police department personnel in the era of terrorism* (Doctoral dissertation). Retrieved from ProQuest Dissertation and Theses Global database (UMI No. 3315747).

[cl21111-bib-0636] Terrorism Early Warning Group . (2005). *The Los Angeles terrorism early warning group conference: Terrorism, global security, and the law*. Los Angeles, CA: Los Angeles County Sheriffʼs Department.

[cl21111-bib-0637] Textile Protection and Comfort Center (T‐PACC) . (2018). *Advanced multi‐threat base ensemble for responders (AMBER™): Final report*. Retrieved from https://www.dhs.gov/sites/default/files/publications/848_AMBER-Final-Report_180305-508.pdf

[cl21111-bib-0638] The Global Center on Cooperative Security . (2015). *Countering violent extremism and promoting community resilience in the Greater Horn of Africa: An action agenda*. Republic of Turkey, Norwegian Ministry of Foreign Affairs. Retrieved from https://www.globalcenter.org/wpcontent/uploads/2015/05/HoA_Action_Agenda_Low_Res.pdf

[cl21111-bib-0639] Thompson, D. J. (2017). *Southeastern state local law enforcement preparedness in domestic terrorism interdiction: A quantitative ex‐post facto study* (Doctoral dissertation). Retrieved from ProQuest Dissertations and Theses Global database. (ProQuest No. 10259452).

[cl21111-bib-0640] Timbers, J. , Wilkinson, D. , Hause, C. C. , Smith, M. L. , Zaidi, M. A. , Laframboise, D. , & Wright, K. E. (2014). Elimination of bioweapons agents from forensic samples during extraction of human DNA. Journal of Forensic Sciences, 59(6), 1530–1540. 10.1111/1556-4029.12561 25069670

[cl21111-bib-0641] Tischer, B. , & Feik, R. (2007). Das System der informationellen Befugnisse der Polizei. Zeitschrift für öffentliches Recht, 62(2), 319–320.

[cl21111-bib-0642] Toddington, M. (2004). Seaport security the need for international cooperation. Intersec: the Journal of International Security, 14(3), 70–74.

[cl21111-bib-0643] Toelstede, B. (2019). Democracy interrupted: The anti‐social side of intensified policing. Democracy and Security, 15(2), 137–149. 10.1080/17419166.2018.1493992

[cl21111-bib-0644] Tomlinson, M. (2012). From counter‐terrorism to criminal justice: Transformation or business as usual? The Howard Journal of Criminal Justice, 51(5), 442–457. 10.1111/j.1468-2311.2012.00735.x

[cl21111-bib-0645] Tutun, S. , Khasawneh, M. T. , & Zhuang, J. (2017). New framework that uses patterns and relations to understand terrorist behaviors. Expert Systems With Applications, 78, 358–375. 10.1016/j.eswa.2017.02.029

[cl21111-bib-0646] Tyler, T. R. (2012). Toughness vs. fairness: Police policies and practices for managing the risk of terrorism. In C. Lum & L. W. Kennedy (Eds.), Evidence‐Based Counterterrorism Policy (pp. 353–363). New York, NY: Springer. 10.1007/978-1-4614-0953-3_15

[cl21111-bib-0647] Tyler, T. R. , Schulhofer, S. , & Huq, A. Z. (2010). Legitimacy and deterrence effects in counter‐terrorism policing: A study of Muslim Americans. Law and Society Review, 44(2), 365–402.

[cl21111-bib-0648] U.S. Department of Homeland Security . (2010). *Moving towards credentialing interoperability: Case studies at the state, local, and regional levels*. Retrieved from https://www.dhs.gov/sites/default/files/publications/st-credentialing-interoperability.pdf

[cl21111-bib-0649] *U.S. Department of Homeland Security (2011). Empowering local partners to prevent violent extremism in the United States. Washington DC: The White House. Retrieved from https://www.dhs.gov/sites/default/files/publications/empowering_local_partners.pdf

[cl21111-bib-0650] U.S. Department of Homeland Security . (2012). *2012 National network of fusion centers: Final report*. Retrieved from https://www.dhs.gov/sites/default/files/publications/2012%20National%20Network%20of%20Fusion%20Centers%20Final%20Report.pdf

[cl21111-bib-0651] U.S. Department of Homeland Security . (2013). *21st Century border: 2013 Action items and progress report*. Retrieved from https://www.dhs.gov/sites/default/files/publications/21cb-progress-report-2013_0.pdf

[cl21111-bib-0652] U.S. Department of Homeland Security . (2014a). *2014 National network of fusion centers: Final report*. Retrieved from https://www.dhs.gov/sites/default/files/publications/2014%20National%20Network%20of%20Fusion%20Centers%20Final%20Report_1.pdf

[cl21111-bib-0653] U.S. Department of Homeland Security . (2014b). *2014 Quadrennial homeland security review (QHSR)*. Retrieved from https://www.dhs.gov/sites/default/files/publications/2014-qhsr-final-508.pdf

[cl21111-bib-0654] U.S. Department of Homeland Security . (2015a). *2015 National network of fusion centers: Final report*. Retrieved from https://www.dhs.gov/sites/default/files/publications/2015%20Final%20Report%20Section%20508%20Compliant.pdf

[cl21111-bib-0655] U.S. Department of Homeland Security . (2015b). *Federal protective service annual report: Fiscal year 2015*. Retrieved from https://www.dhs.gov/sites/default/files/publications/Federal%20Protective%20Service%20Annual%20Report%20508%20Compliant%20FY2015.pdf

[cl21111-bib-0656] U.S. Department of Homeland Security . (2017). *MANET operational field test with the New York City Police Department (NYPD) Emergency Service Unit (ESU) after action report*. Retrieved from https://www.dhs.gov/sites/default/files/publications/OIC_MANET-NYPD-ESU-AAR_170921-508_0.pdf

[cl21111-bib-0657] U.S. Department of Homeland Security . (2018a). *Behavior detection visual search task analysis project visual search battery report* (RTI Project No. 0214428.006.000). RTI International. Retrieved from https://www.dhs.gov/sites/default/files/publications/940_OPSR_BDO-Visual-Search_Final-Report_1805-508.pdf

[cl21111-bib-0658] U.S. Department of Homeland Security . (2018b). *Next generation first responder (NGFR) case study: Situational awareness*. Retrieved from https://www.dhs.gov/sites/default/files/publications/804_NGFR_Case%20Study-Situational-Awareness_201805-508.pdf

[cl21111-bib-0659] U.S. Department of Homeland Security . (2018b). *Report on alerting tactics science and technology directorate*. Retrieved from https://www.dhs.gov/sites/default/files/publications/1051_IAS_Report-on-Alerting-Tactics_180807-508.pdf

[cl21111-bib-0660] UK Secretary of State for the Home Department . (2011). *Prevent strategy*. Retrieved from http://www.homeoffice.gov.uk/publications/counter-terrorism/prevent/prevent-strategy/prevent-strategy-review?view=Binary

[cl21111-bib-0661] Ullah, F. (2017). Critical assessment: Reforms and significance of effective police training to counter terrorism in Khyber Pakhtunkhwa Pakistan. Pakistan Journal of Criminology, 9(3), 121–132.

[cl21111-bib-0662] Umek, P. (2013). Novejse teorije psihologije mnozice in taktika policije. Varstvoslovje, 15(1), 29–44.

[cl21111-bib-1181] United States Agency for International Development (USAID) . (2016). Department of State & USAID joint strategy on countering violent extremism. Washington, DC: United States of America Department of State.

[cl21111-bib-0663] United States Government Accountability Office (2004). *FBI transformation: data inconclusive on effects of shift to counterterrorism related priorities on traditional crime enforcement* (Paper No. GAO‐04‐1036). Washington, DC: U.S. Government Accountability Office.

[cl21111-bib-0664] United States Government Accountability Office (2012). *Terrorist watchlist: Routinely assessing impacts of agency actions since the December 25, 2009, attempted attach could help inform future efforts* (Report No. GAO‐12‐476). Washington, DC: United States Government Accountability.

[cl21111-bib-0665] United States Government Accountability Office . (2013). *Combating terrorism: DHS should take action to better ensure resources abroad align with priorities*. Retrieved from https://www.gao.gov/assets/660/658132.pdf

[cl21111-bib-0666] Urciuoli, L. , Paulraj, A. , & Näslund, D. (2013). The role of the law enforcement agencies in transport security, a survey with Swedish operators. Logistics Research, 6(4), 145–157. 10.1007/s12159-013-0102-8

[cl21111-bib-0667] Vallejo, G. , & Alonso, A. (2009). La identificación genética en grandes catástrofes: avances científicos y normativos en España [Genetic identification in mass disasters: Scientific advances and guidelines in Spain}. Revista Española de Medicina Legal, 35(1), 19–27.

[cl21111-bib-0668] van der Velden, M. , & Krasenberg, J. (2018). *Embedding social and health care workers into institutional structures*. Paper presented at RAN H&SC Meeting, Munich, Germany.

[cl21111-bib-0669] Vardalis, J. J. , & Waters, S. N. (2010). An analysis of Texas Sheriffs' opinions concerning domestic terrorism. Journal of Homeland Security and Emergency Management, 7(1), 1–15. 10.1108/S0163-786X(2010)0000030006

[cl21111-bib-0670] Vaughn Lee, J. (2010). Policing after 9/11: Community policing in an age of homeland security. Police Quarterly, 13(4), 347–366. 10.1177/1098611110384083

[cl21111-bib-0671] Vermeulen, F. (2014). Suspect communities—Targeting violent extremism at the local level: policies of engagement in Amsterdam, Berlin, and London. Terrorism and Political Violence, 26(2), 286–306. 10.1080/09546553.2012.705254

[cl21111-bib-0672] Vickers, J. N. , & Lewinski, W. (2012). Performing under pressure: Gaze control, decision making and shooting performance of elite and rookie police officers. Human Movement Science, 31(1), 101–117. 10.1016/j.humov.2011.04.004 21807433

[cl21111-bib-0673] Victoria Auditor‐General (2009). Preparedness to respond to terrorism incidents: Essential services and critical infrastructure. Melbourne, VIC, Australia: Victorian Auditor‐General's Office.

[cl21111-bib-0674] Vidino, L. (2010). The role of non‐violent Islamists in Europe. Combating Terrorism Center Sentinel, 3(11‐12), 9–11.

[cl21111-bib-0675] Vidino, L. , & Brandon, J. (2012). *Countering radicalization in Europe*. London, UK: International Centre for the Study of Radicalisation.

[cl21111-bib-0676] Wainfan, L. (2010). *Multi‐perspective strategic decision making: Principles, methods, and tools* (Doctoral dissertation). Retrieved from ProQuest Dissertations and Theses Global database. (UMI No. 3421236).

[cl21111-bib-0677] Walters, J. , Budd, C. , Smith, R. G. , Choo, K.‐K. R. , McCusker, R. , & Rees, D. (2011). *Anti‐money laundering and counter‐terrorism financing across the globe: A comparative study of regulatory action* (Research and Public Policy Series No. 113). Canberra, ACT, Australia: Australian Institute of Criminology.

[cl21111-bib-0678] Walton, M. (2014). *Informal transnational police‐to‐police information sharing: Its structure and reform* (Master's thesis). Toronto, On, Canada, York University.

[cl21111-bib-0679] * Warnes, R. (2016). 'Beyond procedural justice': The significance of personal and community relationships in countering terrorist recruitment. In S. Ekici & E. Ragab (Eds.), Countering terrorist recruitment in the context of armed counter‐terrorism operations (pp. 221–238). Amsterdam, The Netherlands: IOS Press.

[cl21111-bib-0680] Watson, T. (2013). *Enforcement and immigrant location choice* (NBER Working Paper No. 19626). Retrieved from http://www.nber.org/papers/w19626

[cl21111-bib-0681] Weine, S. (2013). Building community resilience to violent extremism. Georgetown Journal of International Affairs, 14(2), 81–89.

[cl21111-bib-0682] Weine, S. (2017). *How local law enforcement uses community policing to combat terrorism*. Retrieved from https://www.lawfareblog.com/how-local-law-enforcement-uses-community-policing-combat-terrorism

[cl21111-bib-0683] Weine, S. , & Ahmed, O. (2012). *Building resilience to violent extremism among Somali‐Americans in Minneapolis‐St. Paul*. College Park, MD: National Consortium for the Study of Terrorism and Responses to Terrorism.

[cl21111-bib-0684] Weine, S. , & Braniff, W. (2015). *Report on the national summit on empowering communities to prevent violent extremism*. Washington, DC: Office of Community Oriented Policing Services.

[cl21111-bib-0685] Weine, S. , & Braniff, W. (2017). Countering terrorism. In G. LaFree & J. D. Freilich (Eds.), The handbook of the criminology of terrorism (pp. 450–467). Hoboken, NJ: John Wiley & Sons.

[cl21111-bib-0686] Weine, S. , Eisenman, D. P. , Kinsler, J. , Glik, D. C. , & Polutnik, C. (2017). Addressing violent extremism as public health policy and practice. Behavioral Sciences of Terrorism and Political Aggression, 9(3), 208–221. 10.1080/19434472.2016.1198413

[cl21111-bib-0687] Weine, S. , Eisenman, D. , Glik, D. , Kinsler, J. , & Polutnik, C. (2018). *Leveraging a targeted violence prevention program to prevent violent extremism: A formative evaluation in Los Angeles*. Chicago, IL: University of Illinois at Chicago. Retrieved from https://www.dhs.gov/sites/default/files/publications/862_OPSR_TP_LA-Formative-Evaluation_180817-508.pdf

[cl21111-bib-0688] Weine, S. , Ellis, B. H. , Haddad, R. , Miller, A. B. , Lowenhaupt, R. , & Polutnik, C. (2015a). *Best practices for developing resilient communities and addressing violent extremism*. Retrieved from http://www.start.umd.edu/pubs/START_LessonsLearnedfromMentalHealthAndEducation_BestPracticesforResilientCommunities_Oct2015.pdf

[cl21111-bib-0689] * Weine, S. , Ellis, B. H. , Haddad, R. , Miller, A. B. , Lowenhaupt, R. , & Polutnik, C. (2015b). *Lessons learned from mental health and education: Identifying best practices for addressing violent extremism*. Retrieved from https://www.start.umd.edu/pubs/START_LessonsLearnedfromMentalHealthAndEducation_FullReport_Oct2015.pdf

[cl21111-bib-0690] Weine, S. , Ellis, B. H. , Haddad, R. , Miller, A. B. , Lowenhaupt, R. , & Polutnik, C. (2015c). *Supporting a multidisciplinary approach to addressing violent extremism: What role can education professionals play?* Retrieved from http://www.start.umd.edu/pubs/START_LessonsLearnedfromMentalHealthAndEducation_EducatorSummary_Oct2015.pdf

[cl21111-bib-0691] Weine, S. , Ellis, B. H. , Haddad, R. , Miller, A. B. , Lowenhaupt, R. , & Polutnik, C. (2015d). *Supporting a multidisciplinary approach to addressing violent extremism: What role can mental health professionals play?* Retrieved from http://www.start.umd.edu/pubs/START_LessonsLearnedfromMentalHealthAndEducation_MentalHealthSummary_Oct2015.pdf

[cl21111-bib-0692] Weine, S. , Erez, E. , & Polutnik, C. (2019). *Helpful and harmful practices for addressing alleged transnational crimes in Somali‐American communities* (Document No. 252137). Retrieved from https://www.ncjrs.gov/pdffiles1/nij/grants/252137.pdf

[cl21111-bib-0693] Weine, S. , Henderson, S. , Shanfield, S. , Legha, R. , & Post, J. (2013). Building community resilience to counter violent extremism. Democracy and Security, 9(4), 327–333. 10.1080/17419166.2013.766131

[cl21111-bib-0694] * Weine, S. , Polutnik, C. , & Younis, A. (2015). *The role of community policing in countering violent extremism* (Research Brief). Retrieved from http://www.start.umd.edu/pubs/STARTResearchBrief_CommunityPolicing_Feb2015.pdf

[cl21111-bib-0695] Weine, S. , Polutnik, C. , & Younis, A. (2015). *Understanding communities' attitudes towards CVE* (Research Brief). Retrieved from https://www.start.umd.edu/pubs/STARTResearchBrief_UnderstandingCommunitiesAttitudesTowardCVE_Feb2015.pdf

[cl21111-bib-0696] * Weine, S. , Younis, A. , & Polutnik, C. (2017). *Community policing to counter violent extremism: A process evaluation in Los Angeles*. Retrieved from https://www.start.umd.edu/pubs/START_CSTAB_CommunityPolicingtoCounterViolentExtremism_July2017.pdf

[cl21111-bib-0697] Weisburd, D. , & Braga, A. A. (2006). Police innovation: Contrasting perspectives. Cambridge, UK: Cambridge University Press.

[cl21111-bib-0698] Weisburd, D. , Feucht, T. , Hakimi, I. , Mock, L. & Perry, S. (Eds.). (2011). To protect and to serve: Policing in an age of terrorism. New York, NY: Springer.

[cl21111-bib-0699] Weisburd, D. , Hasisi, B. , Jonathan, T. , & Aviv, G. (2010). Terrorist threats and police performance: A study of Israeli communities. The British Journal of Criminology, 50(4), 725–747. 10.1093/bjc/azp064

[cl21111-bib-0700] Weisburd, D. , Jonathan, T. , & Perry, S. (2009). The Israeli model for policing terrorism: Goals, strategies, and open questions. Criminal Justice and Behavior, 36(12), 1259–1278. 10.1177/0093854809345597

[cl21111-bib-0701] Whelan, C. W. , Wagstaff, G. , & Wheatcroft, J. M. (2014). High stakes lies: Police and non‐police accuracy in detecting deception. Psychology, Crime and Law, 21(2), 127–138. 10.1080/1068316X.2014.935777

[cl21111-bib-0702] Whitaker, R. (2008). Arar: The affair, the inquiry, the aftermath . IRPP Policy Matters, 9(1). Retrieved from https://www.publicsafety.gc.ca/cnt/rsrcs/lbrr/ctlg/dtls-en.aspx?d=PS&i=19184338

[cl21111-bib-0703] White, G. , Mazerolle, L. , Porter, M. D. , & Chalk, P. (2014). *Modelling the effectiveness of counter‐terrorism interventions (Trends and Issues in Crime and Criminology No. 475)*. Canberra, ACT, Australia: Australian Institute of Criminology.

[cl21111-bib-0704] White, M. P. , Cohrs, J. C. , & Göritz, A. S. (2008). The police officer's terrorist dilemma: Trust resilience following fatal errors. European Journal of Social Psychology, 38(6), 947–964. 10.1002/ejsp.488

[cl21111-bib-0705] White, S. (2018). *Policing reforms in the aftermath of conflict* (Policy Brief). Washington, DC: Global Center on Cooperative Security.

[cl21111-bib-0706] Wiefelspütz, D. (2007). Der Einsatz bewaffneter deutscher Streitkräfte im Ausland [The deployment of German armed forces abroad]. Archiv des öffentlichen Rechts, 132(1), 44–94.

[cl21111-bib-0707] Williams, M. J. , Horgan, J. G. , Evans, W. P. , & Bélanger, J. J. (2018). Expansion and replication of the theory of vicarious help‐seeking. Behavioral Sciences of Terrorism and Political Aggression, 12, 1–29. 10.1080/19434472.2018.1546217

[cl21111-bib-0708] Willis, H. H. , Predd, J. B. , Davis, P. K. , & Brown, W. P. (2010). *Measuring the effectiveness of border security between ports of entry*. Santa Monica, CA: RAND Homeland Security Defense Center.

[cl21111-bib-0709] Willits, D. W. , & Nowacki, J. S. (2014). Police organisation and deadly force: An examination of variation across large and small cities. Policing and Society: An International Journal of Research and Policy, 24(1), 63–80. 10.1080/10439463.2013.78431

[cl21111-bib-0710] Wilner, A. , & Rigato, B. (2017). The 60 days of PVE campaign: Lessons on organizing an online, peer‐to‐peer, counter‐radicalization program. Journal for Deradicalization, 12, 227–268.

[cl21111-bib-0711] Woltman, P. , & Haanstra, W. (2017). *A holistic local approach to preventing radicalisation in Helsinki*. Paper presented at RAN Study Visit, Helsinki, Finland.

[cl21111-bib-0712] *World Organisation for Resource Development & Education (WORDE) . The Montgomery County model. Retrieved from https://www.dhs.gov/sites/default/files/publications/Montgomery%20County%20MD%20Community%20Partnership%20Model-WORDE%20Report_0.pdf

[cl21111-bib-0713] van der Woude, M. , & Brouwer, J. (2017). Searching for “illegal” junk in the trunk: Underlying intentions of (cr)immigration controls in Schengen's inernal border areas. New Criminal Law Review, 20(1), 157–179. 10.1525/nclr.2017.20.1.157

[cl21111-bib-0714] Yang, S.‐M. , & Jen, I.‐C. (2018). An evaluation of displacement and diffusion effects on eco‐terrorist activities after police interventions. Journal of Quantitative Criminology, 34, 1103–1123. 10.1007/s10940-017-9367

[cl21111-bib-0715] Yildiz, S. , & Goktepe, F. (2011). The role of social projects in preventing radicalization in terrorist organizations. In I. Bal , S. Ozeren & M. A. Sozer (Eds.), Multi‐faceted approach to radicalisation in terrorist organisations, NATO Science for Peace and Security Series E: Human and social dynamic (pp. 77–95). Clifton, VA: IOS Press. 10.3233/978-1-60750-824-3-77

[cl21111-bib-0716] Yıldız, S. , & Sahin, B. (2010). Toplum destekli polisliğin terörle mücadelede kullanımı ve bir alan çalışması olarak mardin örneğI. In M. A. Sözer (Ed.), Toplum destekli polislikteki yeri Toplum: Suç ve Güvenlik. Ankara, Turkey: Adalet Yayınevi.

[cl21111-bib-0717] Žaberl, M. , Pozderec, F. , & Oberman, I. (2017). Preprečevanje nevarnosti kot podlaga za izvajanje varnostnih pooblastil [Preventing danger as a basis for the implementation of police safety power]. Varstvoslovje, 19(3), 273–292.

[cl21111-bib-0718] Zanini, M. T. F. , dos Santos, M. C. C. , & Lima, D. F. P. (2015). A influência do estilo de liderança consultivo nas relações de confiança e comprometimento no Batalhão de Operações Policiais Especiais do Rio de Janeiro. Revista de Administração, 50(1), 105–120. 10.5700/rausp1187

[cl21111-bib-0719] Zeiger, S. (Ed.), 2016). Expanding research on CVE. Abu Dhabi, United Arab Emirates: Hedayah.

[cl21111-bib-0720] Zeiger, S. (Ed.). (2018). Expanding the evidence base for preventing and countering violent extremism: Research solutions. Abu Dhabi, United Arab Emirates: Hedayah.

[cl21111-bib-0721] Zeiger, S. & Aly, A. (Eds.). (2014). Countering violent extremism: Developing an evidence‐base policy and practice. Perth, WA, Australia: Curtin University.

[cl21111-bib-0722] Zhong, L. R. , & Kebbell, M. R. (2018). Detecting truth, deception, and innocence in a mock counter‐terrorism scenario: The use of forced‐choice testing. Journal of Policing, Intelligence and Counter Terrorism, 13(1), 80–92. 10.1080/18335330.2018.1438640

[cl21111-bib-0723] Zureik, E. & Salter, M. B. (Eds.). (2005). Global surveillance and policing borders, security, identity. Devon, UK: Willan Publishing.

[cl21111-bib-0724] Zureik, E. , & Salter, M. B. (2005). Global surveillance and policing: Borders, security, identity. Portland, OR: Wilan.

[cl21111-bib-0725] 2001‐2002 annual report of the executive director . (2002). *Police Chief, 69*(10), 56‐63.

[cl21111-bib-0726] A different mindset: negotiation challenges for today's critical incident responders . (2002). *Royal Canadian Mounted Police Gazette, 64*(2), 22–24.

[cl21111-bib-0727] A different perspective . (2003). *Police Life: Victoria Police Magazine*, 12, 26–27.

[cl21111-bib-0728] A life of vice. Race against crime . (2003). *Jane's Police Review, 111*(5735), 20–22, 24–25. Profiling terror. Crime sweep. (2003). Jane's Police Review, 111(5737), 18–20, 24–25. Martyn, A. (2003). The Amrozi Bali bombing case: Is Indonesia's anti‐terrorism law unconstitutional? (Research Note No. 14). Canberra: Department of the Parliamentary Library. Taking charge. Help the aged. (2003). Jane's Police Review, 111(5737a), 18–20, 26–27. Street life. Ground force. Jane's Police Review, 111(5738), 20–21, 22–23.

[cl21111-bib-0729] A potent force for change . (2004). *Security Management Today*, 7, 35–36.

[cl21111-bib-0730] Abdul‐Ra'uf, B. (2006). U.S. police/African American community relations and racial profiling: Doing anthropology at home. ERCES Online Quarterly Review, 3(1). https://web.archive.org/web/20100226202618/ http://www.erces.com/journal/Journal.htm

[cl21111-bib-0731] Abru, E. (2002). Diamonds: A crook's best friend. New South Wales Police News, 82(8), 25–27. August.

[cl21111-bib-0732] Addressing organised criminality . (2002). *Policing Today, 8*(1), 15–16.

[cl21111-bib-0733] Adler, R. (2003). Air force security police: A modern paradigm or civilian law enforcement? Journal of Counterterrorism & Homeland Security International, 10(1), 60–62.

[cl21111-bib-0734] Afghanistan, an VIII . (2009). *Les Cahiers de Mars*, 199, 13–84.

[cl21111-bib-0735] Ahlin, E. , Beckman, K. , Gibbs, J. , Gugino, M. , & Varriale, J. (2007). *Trends in research on community policing 2000‐2004: A review of the published literature*. Paper presented at the annual meeting of the American Society of Criminology, Atlanta, GA.

[cl21111-bib-0736] Ajzenstadt, M. (2008). *Risk and counter‐terrorist policies in Israel*. Paper presented at the International Sociological Association conference, Barcelona, Spain.

[cl21111-bib-0737] Akdogan, H. , & Yasin, K. (2008). *Positive and negative obligations of Democratic States while struggling with terrorism*. Paper presented at the annual meeting of the American Society of Criminology, St. Louis, MO.

[cl21111-bib-0738] Allan, M , & Geva, N. (2011). *An experimental analysis of utilitarian considerations of public preferences for military and legal policies*. Paper presented at the International Society of Political Psychology Conference, Instanbul, Turkey.

[cl21111-bib-0739] Almonte, R. (2018). Planning meeting to launch the International Organized Crime and Gang Investigators Association (IOCGIA). Journal of Gang Research, 25(3), 74.

[cl21111-bib-0740] Anarumo, M. (2007). *Terrorist target selection and event predictors: Results from the National Terrorism Study*. Paper presented at the annual meeting of the American Society of Criminology, Atlanta, GA.

[cl21111-bib-0741] Australian Attorney‐General's Department (2010). Enhanced regulation of alternative remittance dealers: Discussion paper (Australian Transaction Reports and Analysis Centre: 1‐6). Canberra, ACT, Australia: Attorney‐General's Department.

[cl21111-bib-0742] Bailey, A. C. (2012). Psychological effects of exposure to stress in law enforcement: Advances in psychology research. New York, NY: Nova Science Publishers.

[cl21111-bib-0743] Baker, D. (2006). Forms of exclusion: Racism and community policing in Canada. Oshawa, ON, Canada: de Sitter.

[cl21111-bib-0744] Barak, G. (Ed.). (2007). Violence, conflict & world order: Critical conversations on state‐sanctioned justice. Lanham, MD: Rowman & Littlefield Publishers.

[cl21111-bib-0745] Barton, P. , & Nissanka, V. (2003). Cyber‐crime ‐ criminal offence or civil wrong? Computer Law & Security Review, 19(5), 401–405. 10.1016/S0267-3649(03)00509-0

[cl21111-bib-0746] Bayley, D. H. , & Perito, R. (2010). The police in war: Fighting insurgency, terrorism, and violent crime. Boulder, CO: Lynne Rienner Publishers.

[cl21111-bib-0747] Beauchesne, L. (2010). *La police communautaire: un Écran de fumée*. Ottawa, ON, Canada: Public Safety Canada.

[cl21111-bib-0748] Bennett, C. W., Jr. , & Snead, P. H. (2002). In challenging times, law enforcement must be the model of ethics in action. The Police Chief, 69(2), 37.

[cl21111-bib-0749] Bennett, J. T. (2006). Homeland security scam. New Brunswick, NJ: Transaction Publishers.

[cl21111-bib-0750] Berger, W. B. (2002). IACP actions in response to September 11. The Police Chief, 69(9), 6.

[cl21111-bib-0751] Berhanu, S. , Nolan, J. , & Barnett‐Ryan, C. (2008). *Preempting domestic terrorist attacks: Institutionalizing proactive policing*. Paper presented at the annual meeting of the American Society of Criminology, St. Louis, MO.

[cl21111-bib-0752] Berksoy, B. (2009). Police sub‐culture as the manifestation of state strategies: An evaluation of the hegemonic discourses prevalent within the police organization in post‐1960 Turkey. Toplum ve Bilim, 114, 98–130.

[cl21111-bib-0753] Bianchi, F. (2007). Internal security and riot control vehicles. Military Technology, 31(11), 84.

[cl21111-bib-0754] Bischoff, M. (2005). *Accountability in policing and surveillance in the information age: Boundaries and boundary‐crossing in global terrorism and counter‐terrorism* (Doctoral dissertation). Melbourne, VIC, Australia, La Trobe University.

[cl21111-bib-0755] Bjørgo, T. , & Horgan, J. (2008). Leaving terrorism behind: Individual and collective disengagement. London, UK: Routledge.

[cl21111-bib-0756] Bodrero, D. (2002). Law enforcement's new challenge to investigate, interdict, and prevent terrorism. Police Chief, 69(2), 41–48.

[cl21111-bib-0757] Bojanic, N. , & Haris, N. (2003). The role of police managing crises situation occurred by terrorism. Kriminalisticke teme, 2(1‐2), 307–335.

[cl21111-bib-0758] Bork, R. (2003). Liberty and terrorism: Avoiding a police state. Current, 458, 9–15.

[cl21111-bib-0759] Borum, R. (2003). Understanding the terrorist mindset. Crime and Justice International: Worldwide News and Trends, 19(77), 28–30.

[cl21111-bib-0760] Bott, K. , & Koch‐Arzberger, C. (2012). Der faktor furcht ‐ Auswirkungen der islamistischen terrorgefahr: Befunde einer repräsentativen studie in Hessen [The fear factor: The effect of Islamist terrorism findings of a representative study in Hesse/Germany]. Monatsschrift für Kriminologie und Strafrechtsreform, 95(2), 132–141.

[cl21111-bib-0761] Brace, M. (2003). Down under and up above. Money Laundering Bulletin, 101, 8–10.

[cl21111-bib-0762] Brems, L. (2004). Policing Needham: A story of suburban cops. Orlando, FL: Rivercross.

[cl21111-bib-0763] Bridges, D. (2002). It's a police problem: The terrorist threat's impact on state and local law enforcement. The Police Chief, 69(2), 35–38.

[cl21111-bib-0764] Brown, S. D. (2008). Combating international crime: The longer arm of the law, London, UK: Routledge‐Cavendish.

[cl21111-bib-0765] Buck, C. (2002). Protecting California. California Journal, 33, 33–37.

[cl21111-bib-0766] Bye, R. J. , Almklov, P. G. , Nyheim, O. M. , Gilberg, A. , & Johnsen, S. O. (2017). *The failure of knowledge‐based task performance—A study of the police emergency response during the 22/7 terror attacks in Norway*. Paper presented at the 26th European Safety and Reliability Conference, Glasgow, UK.

[cl21111-bib-0767] Byrne, K. (2006). The enemy within: The psychological nature of extremists. Jane's Police Review, 114(5879), 26–27.

[cl21111-bib-0768] Carter, D. L. (2012). Law enforcement intelligence and national security intelligence exploring the differences. IALEIA Journal, 21(1), 1–14.

[cl21111-bib-0769] Cassara, J. A. (2006). Hide and seek: Intelligence, law enforcement, and the stalled war on terrorist finance. Washington, DC: Potomac Books.

[cl21111-bib-0770] Cavaillès, C. (2005). Quelle politique européenne contre le terrorisme? [What should be Europe's policy to combat terrorism?]. Revue du Marche Commun et de l'Union Europeenne, 489, 382–384.

[cl21111-bib-0771] CBRN response in the UK . (2006). *Crisis Response Journal, 2*(4), 28–29.

[cl21111-bib-0772] Chapman, S. (2003). Securing the nation: A business issue. Information Week, 47, 34–38.

[cl21111-bib-0773] Chappell, A. T. (2008) *Community policing and Homeland Security: Friend or foe?* Paper presented at the annual meeting of the American Society of Criminology, St. Louis, MO.

[cl21111-bib-0774] Chermak, S. , Freilich, J. , & Caspi, D. (2009). *Consider the unintended consequences of proposed interventions/responses to extremist and terrorist groups*. Paper presented at the American Society of Criminology Annual Meeting, Philadelphia, PA.

[cl21111-bib-0775] Chiabi, D. (2007). *Policing non‐rule of law societies*. Paper presented at the annual meeting of the American Society of Criminology, Atlanta, GA.

[cl21111-bib-0776] Chocquet, C. (2003). Terrorism & organized crime. Paris, France: L'Harmattan.

[cl21111-bib-0777] Christensen, J. (2008). Developing intelligence led law enforcement ‐‐ An example from the Baltic Sea Region. HEUNI Papers, 28, 20–21.

[cl21111-bib-0778] Ciprani, F. , Moroni, M. , & Conte, G. (2014). Fattori di rischio nell'attivita di Polizia: Criticita operative nei protocolli di sorveglianza [Risk factors in police activities: Operational criticism in surveillance programs]. Giornale Italiano di Medicina del Lavoro ed Ergonomia, 36(4), 397–399. 25558742

[cl21111-bib-0779] Coaffee, J. (2008). Redesigning counter‐terrorism for 'soft' targets. Royal United Services Institute (RUSI) Homeland Security and Resilience Monitor, 7(2), 16–17.

[cl21111-bib-0780] Cone, J. , Bowler, R. , Li, J. , Kornblith, E. , Shaikh, A. , & Gocheva, V. (2014). Chronic probable posttraumatic stress disorder among police registrants in the World Trade Centre Health Registry ten years after 9/11/01. Occupational and Environmental Medicine, 71(1), A55–A56.

[cl21111-bib-0781] Consortium, U.S. attorney help reduce gun violence . (2004). *Community Links*, 1, 1, 4.

[cl21111-bib-0782] Coopération et sécurité en Europe [Cooperation and security in Europe] . (2005). *Revue de la Gendarmerie Nationale*, 215, 37–79.

[cl21111-bib-0783] Coosemans, T. (2004). Promouvoir «l'Europe du renseignement»: Nécessité et perspectives [Promoting a ‘European intelligence community’: Necessities and outlook]. Revue du Marche Commun et de l'Union Europeenne, 477, 241–252.

[cl21111-bib-0784] Copes, H. (2005). Policing and stress. Upper Saddle River, NJ: Pearson Prentice Hall.

[cl21111-bib-0785] Corbo Crehan, A. (2003). War and terrorism: What governments can learn from police: Viewpoint. New South Wales Police News, 83(4), 23–25.

[cl21111-bib-0786] Corum, J. , & Wray, J. (2003). Airpower in small wars: fighting insurgents and terrorists. Lawrence, KS: University Press of Kansas.

[cl21111-bib-0787] Crowther, D. (2005). EKAM Greek police anti‐terrorist unit. Combat and Survival Magazine, 17(2), 21–26.

[cl21111-bib-0788] Cue, C. (2009, November). *Streamlining criminal intelligence sharing among federal, state, local and tribal agencies*. Paper presented at the American Society of Criminology Annual Meeting, Philadelphia, PA.

[cl21111-bib-0789] Cullen, S. C. (2008). Muslim extremism in the Caribbean Basin: The threat to the United States. Journal of Counterterrorism & Homeland Security International, 14(2008), 20–23. 3.

[cl21111-bib-0790] Das, D. K. , & Jiao, A. Y. (2005). Public order: A global perspective. Upper Saddle River, NJ: Pearson Prentice Hall.

[cl21111-bib-0791] Das, D. K. & Kratcoski, P. C. (Eds.), 2008). Meeting the challenges of global terrorism: Prevention, control, and recovery. Lanham, MD: Lexington Books.

[cl21111-bib-0792] Davis, A. (2003). Thailand cracks down on illicit arms trade. Jane's Intelligence Review, 15(12), 30–35.

[cl21111-bib-0793] Dean, G. J. , & Gottschalk, P. (2007). Knowledge management in policing and law enforcement: Foundations, structures, applications. Oxford, UK: Oxford University Press.

[cl21111-bib-0794] Delattre, E. (2006). Character and cops: Ethics in policing. Washington, DC: AEI Press.

[cl21111-bib-0795] DeNevi, D. , & Campbell, J. H. (2004). Into the minds of madmen: How the FBI's Behavioral Science Unit revolutionized crime investigation. Amherst, NY: Prometheus Books.

[cl21111-bib-0796] Denying criminals use of the roads . (2003). *Policing Today, 9*(4), 23, 25.

[cl21111-bib-0797] Dhami, M. , & Garcia‐Retamero, R. (2012). *On avoiding framing effects in police decision making*. Paper presented at the American Psychology‐Law Society Conference, San Juan, Puerto Rico.

[cl21111-bib-0798] Difede, J. , Cukor, J. , Olden, M. , Peskin, M. , Palmer, J. , & Wyka, K. (2014). *Enhancing exposure therapies with D‐Cycloserine for the treatment of chronic WTC‐related PTSD*. Paper presented at the annual meeting of the American Psychological Association, Washington, DC.

[cl21111-bib-0799] Doherty, R. (2004). Thin green line: A history of the Royal Ulster Constabulary GC 1922–2001. Barnsley, UK: Pen and Sword Military.

[cl21111-bib-0800] Domash, S. (2003). Nerve Center: NYPD strikes back at terrorists with highly equipped, highly motivated unit. Police: The Law Enforcement Magazine, 27(2), 30–33.

[cl21111-bib-0801] Dorin, A. F. (2008). Jihad and American medicine: Thinking like a terrorist to anticipate attacks via our health system. Westport, CT: Praeger Security International.

[cl21111-bib-0802] Dorriety, J. (2002). Answering the call. Sheriff, 54(2), 16.

[cl21111-bib-0803] Du, J. (2002). On mandatory administrative act of public security (Doctoral dissertation). Indexed in ProQuest Dissertations and Theses Global database. (UMI No. 1027911553).

[cl21111-bib-0804] Dupont, B. , & Massimiliano, M. (2007). *Airport security: A different kind of alliance*. Paper presented at annual meeting of the American Society of Criminology 2007, Atlanta, GA.

[cl21111-bib-0805] Earley, P. , & Shur, G. (2002). WITSEC: Inside the Federal Witness Protection Program. New York, NY: Bantan Books Inc.

[cl21111-bib-0806] Edwards, C. J. (2005). Changing policing theories for 21st century societies. Leichhardt, NSW, Australia: Federation Press.

[cl21111-bib-0807] Ellerbrok, A. A. (2010). *Face recognition systems: From security to convenience*. Paper presented at the International Sociological Association World Congress of Sociology, Gothenburg, Sweden.

[cl21111-bib-0808] Everly, G. S., Jr. , & Lating, J. M. (2017). The Johns Hopkins guide to psychological first aid. Baltimore, MD: Johns Hopkins University Press.

[cl21111-bib-0809] Farfane‐Mendez, C. (2017). *Beyond cartels and kingpins: A theory on behavioral patterns of drug trafficking organizations* (Doctoral dissertation). Retrieved from http://www.escholarship.org/uc/item/37309720

[cl21111-bib-0810] Farrell, T. (2002). Talks resume to end Aceh rebellion. Jane's Intelligence Review, 14(12), 2–3.

[cl21111-bib-0811] Ferrando, L. (2005). Prevalence and correlates of mental disorders in victims, general population and police officers after March 11 terrorist attacks in Madrid. European Neuropsychopharmacology, 15, S557.

[cl21111-bib-0812] Finklea, K. M. (2011). Organized crime in the United States: Trends and issues for congress. In C. M. Webb & P. W. Dobrev (Eds.), Organized crime, gangs and trafficking (pp. 1–43). New York, NY: Nova Science Publishers.

[cl21111-bib-0813] Flemming, M. H. (2005). *UK law enforcement agency use & management of suspicious activity reports: Towards determining the value of the regime*. London, UK: Jill Dando Institute of Crime Science.

[cl21111-bib-0814] Flynt, B. , & Olin, R. (2002). The red, gray, and blue model: A new tool to help law enforcement executives address the transformed security environment. Police Chief, 69(2), 50–52. 55, 58.

[cl21111-bib-0815] Forensics in the field . (2003). *AFP News: Newsletter of the Australian Federal Police*, 113, 5–6.

[cl21111-bib-0816] Franklin‐Webb, J. (2008). In dogs we trust. The Journal of International Security, 18, 8–10.

[cl21111-bib-0817] Freilich, J. D. & LaFree, G. (Eds.). (2016). Criminology theory and terrorism: New applications and approaches. Abingdon, UK: Routledge.

[cl21111-bib-0818] Freilich, J. D. & Newman, G. R. (Eds.), 2009). Reducing terrorism through situational crime prevention (25) New York, NY: Criminal Justice Press.

[cl21111-bib-0819] Frevel, B. (2004). Europäische politik der inneren sicherheit: Ein überblick [European policy of internal security: An overview]. Kriminalpraevention, 8(2), 66–72.

[cl21111-bib-0820] Fuka, J. , Bat'a, R. , & Lešáková, P. (2017, May). *The phenomenon of terrorism as a challenge for the Czech Republic*. Paper presented at the 29th International Business Information Management Association, Vienna, Austria.

[cl21111-bib-0821] Fullerton, C. S. , Ursano, R. J. , & Norwood, A. E. (2004). Planning for the psychological effects of bioterrorism. In R. J. Ursano , A. E. Norwood & C. S. Fullerton (Eds.), Bioterrorism: Psychological and public health interventions (pp. 2–14). Cambridge, UK: Cambridge University Press.

[cl21111-bib-0822] Fussey, P. (2006). *Blurring the boundaries of transgression? Surveillance and counter‐terrorism in London*. Paper presented at the International Sociological Association, Durban, South Africa.

[cl21111-bib-0823] Gagnon, N. (2012a). *Enquêteur(e) principal(e)—contre‐terrorisme: Profil de compétences*. Ottawa, ON, Canada: Conseil Sectoriel de la Police.

[cl21111-bib-0824] Gagnon, N. (2012b). *Investigator*—*counterterrorism: Competency profile*. Ottawa, ON, Canada: Police Sector Council.

[cl21111-bib-0825] Gagnon, N. (2013). Contre‐terrorisme: Liste des tâches professionnelles de l'enquêteur(e) principal. Ottawa, ON, Canada: Police Sector Council.

[cl21111-bib-0826] Galli, F. (2010). *British, French and Italian measures to deal with terrorism: A comparative study*. (Doctoral Dissertation) Indexed in ProQuest Dissertations and Theses Global database. (UMI No. 564826).

[cl21111-bib-0827] Garrett, R. (2002). Analyzing the hidden patterns in call records. Law Enforcement Technology, 29(5), 96–99.

[cl21111-bib-0828] Gaskew, T. (2008). Policing Muslim American communities: A compendium of post 9/11 interviews. Lewiston, NY: Edwin Mellen Press.

[cl21111-bib-0829] Gendron, A. (2008). 'Shocking and sensational': Homegrown terrorism in Canada? Homeland Security and Resilience Monitor, 7(6), 6–11.

[cl21111-bib-0830] Gettler, L. (2003). The new terrorism: every aspect of Western society, including business, is a target for the new terrorists. Management Today, 4, 15–20.

[cl21111-bib-0831] Gibson, B. (2007). The new Home Office. Winchester, UK: Waterside Press.

[cl21111-bib-0832] Going for gold: The Commonwealth Games . (2006). *Association News, 10*(2), 31–34.

[cl21111-bib-0833] Graphia, R. , Kennedy, L. , & Jie, X. (2008). *Enabling and enhancing crime prevention and analysis: Results from a process evaluation of innovative technology*. Paper presented at the annual meeting of the American Society of Criminology, St. Louis, MO.

[cl21111-bib-0834] Gray, A. , Victor, C. , Das, S. , & Faridi, B. (2007). The war on terror's impact on the community. Race & Class, 48(4), 67–71.

[cl21111-bib-0835] Greene, J. R. (2007). Human rights and police discretion: Justice served or denied? In S. Parmentier , E. G. M. Weitekamp & M. Deflem (Eds.), Sociology of Crime Law and Deviance (pp. 147–169). Bingley, UK: Emerald Group Publishing.

[cl21111-bib-0836] Griffin, P. (2009). Intelligence innovation in the Post 9‐11 era: A case study analysis of a regional fusion center. Paper presented at the ASC Annual Meeting, Philadelphia, PA.

[cl21111-bib-0837] Grillo, M. (2008). *Policing for Homeland Security: A new paradigm or a reversion to old ways?* Paper presented at the annual meeting for the American Society of Criminology in St Louis, MO.

[cl21111-bib-0838] Guille, L. (2008). *Police and judicial cooperation in Europe: Europol, Eurojust and the European Judicial Network: master pieces of the European Union's puzzle in justice and home affairs* (Doctoral dissertation). University of Sheffield, Sheffield, UK.

[cl21111-bib-0839] Guiora, A. N. (2008). Fundamentals of counterterrorism. New York, NY: Aspen Publishers.

[cl21111-bib-0840] Gula, P. W. , & Szafran, E. M. (2011). Natural disasters challenge for emergency and rescue services — Lessons learned. Prehospital and Disaster Medicine, 26(Suppl. S1), s108.

[cl21111-bib-0841] Guliaev, V. A. , Zubkov, A. D. , Kuz'min, N. S. , Lochmel, O. I. , Popov, V. N. , Ushakov, I. B. , & Shchegol'kov, A. M. (2003). About interdepartmental program: Rehabilitation of servicemen, citizens dismissed from military service, and law enforcement personnel suffered during combat and antiterrorist operations. Voenno‐Meditsinskii Zhurnal, 324(12), 4–7. 14981997

[cl21111-bib-0842] Habtes, F. F. (2007). Bioterrorism preparedness the interdependent relationship of the local, state, and federal governments. Law Enforcement Executive Forum, 7(6), 187–193.

[cl21111-bib-0843] Hammack, D. W. (2016). Terrorist target selection: Why would they come here? Police Chief, 83(2), 22–29.

[cl21111-bib-0844] Hargrave, R. (2002). A police chaplain's perspective: On the aftermath of September 11. The Police Chief, 69(2), 64–70.

[cl21111-bib-0845] Harrison, K. , & Jones, M. (2007). *Counter‐terrorism: Implications for community corrections*. Paper presented at the annual meeting of the American Society of Criminology, Atlanta, GA.

[cl21111-bib-0846] Hawdon, J. , & Ryan, J. (2007). *Hiding in plain sight: Community organization, naive trust and terrorism*. Paper presented at the Southern Sociological Society, Atlanta, GA.

[cl21111-bib-0847] Hayes, B. (2008). Towards an authoritarian European state? The development and implementation of EU Justice and Home Affairs policy (Doctoral dissertation). University of Ulster, Coleraine, UK.

[cl21111-bib-0848] Haynes, C. (2009). Confidence boost. Jane's Police Review, 117(6028), 24–26.

[cl21111-bib-0849] Henry, K. , & Silva, J. (2002). Enhanced consequence management, planning and support system (ENCOMPASS). Enabling an effective, coordinated response. Emergency Medical Services, 31(4), 52–59. 11963609

[cl21111-bib-0850] Henych, M. C. , Bob, R. , & Mesloh, C. (2002). Colleges and universities as staging grounds for cyber terrorist attacks. Campus Law Enforcement Journal, 32(2), 28–31.

[cl21111-bib-0851] Hickman, M. , & Reaves, B. (2002). Local police and homeland security: Some baseline data. The Police Chief, 69(10), 83–88.

[cl21111-bib-0852] Higginbotham, C. E. (2003). Homeland security preparedness survey. The Police Chief, 70(8), 19–20.

[cl21111-bib-0853] Hillebrand, C. (2010). *The democratic legitimacy of EU counter‐terrorism policing: challenges for parliament and parliamentary scrutiny* (Doctoral dissertation). Aberystwyth University, Aberystwyth, UK.

[cl21111-bib-0854] Hiller, A. (2003). Post September 11 legislation supporting counter‐terrorism measures. Australian Police Journal, 57, 51–69.

[cl21111-bib-0855] Holmqvist‐Jonsäter, C. (2010). *Policing wars: A twenty‐first century discourse on war* (Doctoral dissertation). Indexed in ProQuest Dissertations and Theses Global database. (UMI No. 609581).

[cl21111-bib-0856] Homeland Security funding sources . (2004). *The Police Chief, 71*(2), 23.

[cl21111-bib-0857] Hordern, N. , Bostock, I. , & Chalk, P. (2002). Australia's reaction to Bali. Jane's Intelligence Review, 14(12), 16–19.

[cl21111-bib-0858] Hossfeld, B. , Wurmb, T. , Josse, F. , & Helm, M. (2017). Massenanfall von Verletzten — Besonderheiten von „bedrohlichen Lagen “[Mass casualty incident ‐ Special features of "threatening situations"]. Anasthesiologie Intensivmedizin Notfallmedizin Schmerz, 52(9), 618–629. 10.1055/s-0042-120229 28886611

[cl21111-bib-0859] Hotchkiss, N. S. , & Shawna, N. (2010). *A thin line between terror and hate: Legal distinctions and disparate outcomes*. Paper presented at the International Sociological Association, Gothenburg, Sweden.

[cl21111-bib-0860] Hunt, J. (2009). *The dynamics of lethal force in a raid on a terrorist cell*. Paper presented at the American Society of Criminology Annual Meeting, Philadelphia, PA.

[cl21111-bib-0861] Hunter, C. (2009). Assault IED defeat. The Journal of International Security, 19(4), 20–23.

[cl21111-bib-0862] Hussain, S. (2009, November). *Policing terrorism: Do terrorist arrests deter terrorism?* Paper presented at the American Society of Criminology Annual Meeting, Philadelphia, PA.

[cl21111-bib-0863] Indonesia's changing role in the war on terrorism . (2002). *Jane's Intelligence Review, 14*(11), 46–49.

[cl21111-bib-0864] Intelligence drives investigations: Law enforcement is using intelligence to conduct more accurate investigations . (2003). *Investigator, 16*(Mar/Apr), 32–33.

[cl21111-bib-0865] Ivanusa, T. , & Iztok, P. (2007). Required skills for the security/armed forces in counterterrorism. Varstvoslovje, 9(1‐2), 58–68.

[cl21111-bib-0866] Jackson, T. , & Parkin, W. (2009). *Comparing extreme far‐right homicide cases: The role of snitching*. Paper presented at the American Society of Criminology Annual Meeting, Philadelphia, PA.

[cl21111-bib-0867] Jahankhani, H. (2009). Criminal investigation and forensic tools for smartphones. International Journal of Electronic Security and Digital Forensics, 2(4), 387–406. 10.1504/IJESDF.2009.027671

[cl21111-bib-0868] Japan creeps towards a modern terrorism response . (2006). *Homeland Security Resilience Monitor, 5*(6), 18–19.

[cl21111-bib-0869] Jevsek, A. (2010). Establishment of the National Bureau of Investigation in Slovenia as a response to the contemporary unconventional forms of crime. Revija za Kriminalistiko in Kriminologijo, 61(3), 307–314.

[cl21111-bib-0870] Johnson, M. (2009). *Evaluating the effectiveness of post 9/11 legislation and national security measures*. Paper presented at the American Society of Criminology Annual Meeting, Philadelphia, PA.

[cl21111-bib-0871] Jones, C. (2002). *Military as law enforcers? Coming to terms with the new security environment*. Working paper No. 72, Canberra, VIC, Australia: Australian Defence Studies Centre.

[cl21111-bib-0872] Jones, A. , Bowers, R. , & Lodge, H. D. (2006). Blackstone's guide to the terrorism act 2006. London, UK: Blackstone Press.

[cl21111-bib-0873] Jore, S. H. , & Njå, O. (2009). Protection from half‐criminal windows breakers to mass murderers with nuclear weapons: Changes in the Norwegian authorities' discourses on the terrorism threat. In S. Martorell , C. Guedes Soares & J. Barnett (Eds.), Safety, reliability and risk analysis: Theory, methods and applications (1‐4, pp. 3077–3084). Valencia, Spain: CRC Press‐Taylor & Francis Group.

[cl21111-bib-0874] Julian, R. , & Narramore, T. (2004). *Presentation to the Energy and Communications Australia (ETTA) Counter‐terrorism Workshop*. Paper presented at the Energy and Communications Australia (ETTA) Counter‐terrorism Workshop, Hobart, TAS, Australia.

[cl21111-bib-0875] Jun, J. , Metzler, T. , Henn‐Haase, C. , Best, S. , & Marmar, C. (2008). *Social adjustment and PTSD symptoms in NYC Police Officers exposed to the WTC terrorist attack*. Paper presented at the 24th Annual Meeting of the International Society for Traumatic Stress Studies, Chicago, IL.

[cl21111-bib-0876] Keelty, M. (2002). The operational response to terrorism. Australia and Security Cooperation in the Asia Pacific, 13, 34–37.

[cl21111-bib-0877] Keelty, M. (2006). Indonesian police now the target counter‐terrorism centres in the firing line. *Australian National Security Magazine*, June, 40–41.

[cl21111-bib-0878] Kellman, B. (2007). Bioviolence: Preventing biological terror & crime. New York, NY: Cambridge University Press.

[cl21111-bib-0879] Kelly, S. F. (2003). Internal affairs: Issues for small police departments. FBI Law Enforcement Bulletin, 72(7), 1–6.

[cl21111-bib-0880] Kennedy, M. (2008). Notes from the frontline: 'The Green Light'. Civil Liberty, 212, 16–17.

[cl21111-bib-0881] Kennedy, L. , & Van Brunschot, E. E. (2009). *Risk‐based policing: Applying intelligence to process*. Paper presented at the American Society of Criminology Annual Meeting, Philadelphia, PA.

[cl21111-bib-0882] Kenney, M. (2009). How terrorists learn, From Pablo to Osama: Trafficking and terrorist networks, government bureaucracies, and competitive adaptation. University Park, PA: Penn State University Press.

[cl21111-bib-0883] Kessing, P. V. , & Anderson, L. G. (2018). Forebyggelse af radikalisering i fængsler ‐ menneskeret og retssikkerhed for de indsatte. Nordisk Tidsskrift for Kriminalvidenskab, 105(1), 15–51.

[cl21111-bib-0884] Kilgore, E. L. (2012). *Exploring information sharing within an intelligence community: A case study* (Doctoral dissertation). Indexed in ProQuest Dissertations and Theses Global database. (UMI No. 3536304).

[cl21111-bib-0885] Kirwil, L. , & Rowell, L. (2003, May). *The relation between aggressiveness and emotional reactions to observed violence*. Paper presented at the Annual Meeting of Midwestern Psychological Association, Chicago, IL.

[cl21111-bib-0886] Kisriev, E. , & Ware, R. B. (2002). Irony and political Islam: Dagestan's spiritual directorate. Nationalities Papers, 30(4), 663–689.

[cl21111-bib-0887] Kolesnikova, L. (2006). Metro bombings: Moscow's experience. Crisis Response Journal, 2(4), 22–23.

[cl21111-bib-0888] Kolomeitz, G. (2004). Terrorism and the coronial jurisdiction. Policing Issues and Practice Journal, 12(4), 54–61.

[cl21111-bib-0889] Kratcoski, P. C. & Das, D. K. (Eds.). (2007). Police education & training in a global society. Lanham, MD: Lexington Books.

[cl21111-bib-0890] van Krieken, P. J. (2002). Terrorism & the international legal order: With special reference to the UN, the EU & cross‐border aspect. The Hague, The Netherlands: TMC Asser Press Inc.

[cl21111-bib-0891] Kubink, M. (2002). Fremdenfeindliche straftaten: Ein neuer versuch der polizeilichen registrierung und kriminalpolitischen problem bewältigung [Xenophobic crimes: A new attempt of police recording and political problem solving]. Monatsschrift für Kriminologie und Strafrechtsreform, 85(5), 325–340.

[cl21111-bib-0892] Kulick, A. (2018). Gefahr, Gefährder. Archiv für Öffentlichen Rechts, 143(2), 175–219.

[cl21111-bib-0893] Kulikov, A. S. , & Romashev, Y. S. (2007). On the new Russian anti‐terrorism law. Gosudarstvo i Pravo, 80(7), 40–49.

[cl21111-bib-0894] Larkins, E. R. (2010). *A stray bullet has no address: The institutionalization of terror in a Rio de Janeiro Favela*. Paper presented at the AAA 109th Annual Meeting, New Orleans, LA.

[cl21111-bib-0895] Legault, R. , & Hendrickson, J. (2009). *Preventing firearms use by terrorists in the US through enhanced law enforcement and intelligence cooperation*. Paper presented at the American Society of Criminology Annual Meeting, Philadelphia, PA.

[cl21111-bib-0896] Li, X. X. (2006). *The police power and its regulation: The construction of a homonious society* (Unpublished Master's thesis). Hebei University, Baoding, China.

[cl21111-bib-0897] Lieberman, C. (2007). *Terrorism and deterrence: Construction of a foundation for crime*. Presented at the Annual Meeting of the American Society of Criminology, Atlanta, GA.

[cl21111-bib-0898] Lindsey, D. (2006). To build a more perfect discipline: Ideologies of the normative and the social control of the criminal innocent in the policing of New York City. In S. Pfohl , A. Van Wagenen , P. Arend , A. Brooks & D. Leckenby (Eds.), Culture, power and history: Studies in critical sociology (pp. 223–259). Leiden, The Netherlands: Koninklijke Brill NV.

[cl21111-bib-0899] Link, J. (2002, August). *The nonlinear and complexity dynamics of knowledge management in the classified world*. Paper presented at the Twelfth International Conference of the Society for Chaos Theory in Psychology and Life Science, Portland, OR. Retrieved from https://www.societyforchaostheory.org/conf/conf2002/sctpls02abs.htm

[cl21111-bib-0900] Loumansky, A. (2009). *A death foretold similarities between law and literature*. Paper presented at the American Society of Criminology Annual Meeting, Philadelphia, PA.

[cl21111-bib-0901] Lowe, D. (2010). *Spooks, Provo's and Al Qaeda: An inside study of the UK's integrated Special Branch counter‐terrorism investigations* (Doctoral dissertation). Indexed in ProQuest Dissertations and Theses Global database. (UMI No. U556789).

[cl21111-bib-0902] Lyon, D. (2002). *Technology vs 'terrorism': ID cards, CCTV, and biometric surveillance in the city*. Paper presented at the 15th Conference of the International Sociological Association, Brisbane, QLD, Australia.

[cl21111-bib-0903] Mabrey, D. (2003). Demystifying Hawala: Understanding terrorist financial networks. Crime & Justice International, 19(69), 23–26.

[cl21111-bib-0904] Makarenko, T. (2003). Europe adapts to new terrorist threats. Jane's Intelligence Review, 15(8), 24–27.

[cl21111-bib-0905] Making a positive impression. Leaders in criminal identification. Rebuilding a family's trust . (2003). *Police Life: Victoria Police Magazine*, (August), 4‐6, 16–17, 20–21.

[cl21111-bib-0906] Manley, D. K. , & Bravata, D. M. (2009). A decision framework for coordinating bioterrorism planning: Lessons from the BioNet program. American Journal of Disaster Medicine, 4(1), 49–57. 19378669

[cl21111-bib-0907] Manning, P. K. (2007, November). *On domestic surveillance*. Paper presented at annual meeting of American Society of Criminology, Atlanta, GA.

[cl21111-bib-0908] Marenin, O. (2002). 'A work in progress': Police interagency cooperation homeland security. In M. Pagon (Ed.), Policing in Central and Eastern Europe: Deviance, violence and victimization (pp. 565–584). Ljubljana, Slovenia: College of Police and Security Studies.

[cl21111-bib-0909] Maslesa, R. (2003). Political and safety measures for protection against terrorism. Kriminalisticke Teme, 2(1‐2), 7–18.

[cl21111-bib-0910] Mass. Troopers enlist wireless e‐mail devices in war on terrorism . (2002). *Police, 26*(3), 16.

[cl21111-bib-0911] McAdams, A. J. (2007). Spying on terrorists: Germany in comparative perspective. German Politics & Society, 25(3), 70–88.

[cl21111-bib-0912] McCafferty, M. , & Cragen, J. F. (2008). *Police management culture, individual officer's professional identity and symbolic convergence theory*. Paper presented at the annual meeting of the American Society of Criminology, St. Louis, MO.

[cl21111-bib-0913] McCaffrey, J. , & Kirshner, A. (2016). Government hacking OK? New rules will expand government authority to do so. White‐Collar Crime, 30(12), 3–5.

[cl21111-bib-0914] McDonald, W. F. (2007). *Immigration, crime and justice: A century of development of criminological interests*. Paper presented at the annual meeting of the American Society of Criminology, Atlanta, GA.

[cl21111-bib-0915] McElwee, T. A. (2007). The role of UN police in nonviolently countering terrorism. In R. Senthill & R. Summy (Eds.), Nonviolence: An alternative for defeating global terror(ism) (pp. 187–210). New York, NY: Nova Science Publishers.

[cl21111-bib-0916] Merola, L. (2009). *The legal community's views of terrorism and civil liberties: A survey and an experiment*. Paper presented at the American Society of Criminology Annual Meeting, Philadelphia, PA.

[cl21111-bib-0917] Meyr, E. (2003). The OMON units: Russia's response to crime and terrorism. Intersec: The Journal of International Security, 13(11/12), 359–360. 362.

[cl21111-bib-0918] Michael, L. (2007). *Leadership, security and communities under suspicion*. Paper presented at the annual meeting of the American Society of Criminology, Atlanta, GA.

[cl21111-bib-0919] Milosevic, M. (2003). Problem of penal law protection of the police members from organized terrorism. Kriminalisticke teme, 2(1‐2), 123–137.

[cl21111-bib-0920] Mischkowitz, R. (2013). Fragen an die Kriminologie. aus der Sicht der Polizei [Questions regarding criminology from a police perspective]. Monatsschrift fuer Kriminologie und Strafrechtsreform, 96(2/3), 212–221.

[cl21111-bib-0921] Moeckli, D. (2006). *Whose liberty? The 'war on terrorism', human rights and non‐discrimination* (Unpublished doctoral dissertation). Nottingham, UK: The University of Nottingham.

[cl21111-bib-0922] Monahan, T. (2009). *"Blossoming" surveillance: Exploring DHS fusion centers*. Paper presented at the American Society of Criminology Annual Meeting, Philadelphia, PA.

[cl21111-bib-0923] Monahan, T. (2010). *Mapping concerns with Homeland Security fusion centers*. Paper presented at the XVII ISA World Congress of Sociology: Sociology on the Move, Gothenburg, Sweden.

[cl21111-bib-0924] Moorcraft, P. (2005). Finessing the terrorist threat. In P. Moorcraft , G. Winfield & J. Chisholm (Eds.), Axis of Evil: the War on Terror (pp. 263–269). Barnsley, UK: Pen and Sword Military.

[cl21111-bib-0925] Mors, T. (2008). *Best practices for police relative to recognizing extremists*. Paper presented at the annual meeting of the American Society of Criminology, St Louis, MO.

[cl21111-bib-0926] Mosser, M. E. (2007). *Sharing law enforcement and intelligence information*. Paper presented at the annual meeting of the American Society of Criminology, Atlanta, GA.

[cl21111-bib-0927] Naado, H. O. (2011). Countering violent extremism among Kenyan Muslim youth (Policy Brief). Global Center.

[cl21111-bib-0928] Nancoo, S. E. (Ed.). (2004). Contemporary issues in Canadian policing. Mississauga, ON, Canada: Canadian Educators' Press.

[cl21111-bib-0929] Narr, W.‐D. (2007). Die Zukunft präventiver Illusion ‐ Und ihre repressive Allgegenwart. [The Future of Preventive Illusion ‐ And its Repressive Pervasiveness]. Kriminologisches Journal, 39(3), 214–226.

[cl21111-bib-0930] National security: The APS and the AFP . (2003). AUSPOL: The Official Publication of the Australian Federal Police Association and ALAJA, 1, 25–30.

[cl21111-bib-0931] National threat warning system update‐‐Continued use of NCIC after 2002 Winter Olympic Games . (2002). *The Police Chief, 69*(4), 199.

[cl21111-bib-0932] Necula, I. (2017). Valorificarea datelor biometrice în investigarea actelor de terorism [Biometric data valuation in the investigation of terrorism acts]. Revista Română de Criminalistică, 18(5), 2726–2729.

[cl21111-bib-0933] New techniques used to counter terrorism . (2006). *Australian National Security Magazine*, 41.

[cl21111-bib-0934] Newburn, T. (2003). Handbook of policing. Cullompton, UK: Willan.

[cl21111-bib-0935] Newton, S. J. (2002). Toll of terrorism. *Community Links*, 1–3.

[cl21111-bib-0936] Newton, A. (2011). Emergency medical ambulance services: Anti terrorist response in the 21 century, supporting police firearms units with specially trained critical care paramedics. Prehospital and Disaster Medicine, 26(Suppl. S1), s146.

[cl21111-bib-0937] O'Looney, M. (2003). Deter, detect, respond: How New York fights terror. Policing Today, 9(1), 15–17.

[cl21111-bib-0938] Office of International Criminal Justice . (2002). Animal rights terrorism. Crime and Justice International: Worldwide News and Trends, 18(64), 15–33.

[cl21111-bib-0939] Olson, D. T. (2012). Tactical counterterrorism: The law enforcement manual of terrorism prevention. Springfield, IL: Charles C Thomas Publishing.

[cl21111-bib-0940] On site . (2003). *Security Electronics Magazine: Incorporating Security Australia Magazine*, 6, 62–63.

[cl21111-bib-0941] Oram, G. (Ed.). (2003). Conflict and legality: Policing mid‐twentieth century Europe. London, UK: Francis Boutle.

[cl21111-bib-0942] Ozer, M. , & Duru, H. (2009). *Reading terrorist activities of Turkey from a different perspective*.Paper presented at the American Society of Criminology Annual Meeting, Philadelphia, PA.

[cl21111-bib-0943] Ozguler, M. (2009). *Comparing and assessing the preparedness of police organizations in counter‐terrorism (Netherlands and United Kingdom)* (Doctoral dissertation). Indexed in ProQuest Dissertations and Theses Global database. (UMI No. 3324056).

[cl21111-bib-0944] Parenti, C. (2008). Lockdown America. London, UK: Verso.

[cl21111-bib-0945] Parmar, A. (2009). *Counter‐terrorist policing in London: Locating rhetoric, reality and responses*. Paper presented at the American Society of Criminology Annual Meeting, Philadelphia, PA.

[cl21111-bib-0946] Pearce, L. D. (2010). *Psychosocial considerations in emergency management: A methodology for influencing key decision makers*. Paper presented at the 1010 Conference of International Sociological Association, Gothenburg, Sweden.

[cl21111-bib-0947] Pete, L. (2010). Terrorism and social exclusion: Misplaced risk — common security. Cheltenham, UK: Edward Elgar Publishing.

[cl21111-bib-0948] Pherson, K. H. , & Pherson, R. H. (2013). Critical thinking for strategic intelligence. Los Angeles, CA: Sage publications.

[cl21111-bib-0949] Phillips, A. (2007). Skinheads in America. Law Enforcement Technology, 34(10), 64–71.

[cl21111-bib-0950] Pineiro, E. , & Winzeler, S. (2015). Real gewordene utopie im zerfall: Heterotopologische skizzen zur basler jugendbewegung 1981 [Real has become utopia in decay: Straight topological sketches to basel youth movement 1981]. Forschungsjournal Soziale Bewegungen, 28(1), 169–175.

[cl21111-bib-0951] Plöse, M. (2014a). Warum die ATDG‐Novelle mit der Sicherheitsverfassung des Grundgesetzes unvereinbar ist [Why the ATDG amendment is incompatible with the constitutional protection of basic law]. Vorgänge, 53(4), 153–178.

[cl21111-bib-0952] Plöse, M. (2014b). Was Karlsruhe nicht verbietet, macht Berlin nur dreister [What Karlsruhe doesn't prohibit makes Berlin only bolder]. Vorgänge, 53(2‐3), 122–134.

[cl21111-bib-0953] Police Executive Research Forum . (2003). Anti‐terrorism and disaster response programs exist for law enforcement .

[cl21111-bib-0954] Polikanov, D. V. (2006). Public opinion on the terrorism issue in Russia. Sotsiologicheskie Issledovaniya, 32(2), 57–61.

[cl21111-bib-0955] Pollak, J. D. (2009). *Racial profiling is wrong: A doctrinal comparison of counter‐terrorism racial profiling in Europe and the United States*. Social Science Research Network Working Paper Series. 10.2139/ssrn.1390269

[cl21111-bib-0956] Posner, R. A. (2005). Preventing surprise attacks: Intelligence reform in the wake of 9/11. New York, NY: Rowman & Littlefield.

[cl21111-bib-0957] Power, J. G. C. (2002). A critical examination of state security strategy and its impact on operational policing in Ireland and Euzkadi, and normalisation throughout the European Union. Belford, UK: Ann Arbor.

[cl21111-bib-0958] Preparing for the future. (2003). *AFP News: Newsletter of the Australian Federal Police*, 113, 16–17.

[cl21111-bib-0959] Putting tempest into context. (2003). *AFP News: Newsletter of the Australian Federal Police*, 113, 1–2.

[cl21111-bib-0960] Quillet, N. (2007). Prum treaty. Revue du Marche Commun et de l'Union Europeenne, 513, 660–664.

[cl21111-bib-0961] Rademacher, T. (2017). Im deutschen polizeirecht [Predictive policing]. Archiv für Öffentlichen Rechts, 142(3), 366–416. 10.1628/000389117X15054009148798

[cl21111-bib-0962] Randol, M. A. (2011). The department of Homeland Security intelligence enterprise: Operational overview and oversight challenges for Congress. In T. M. Lindall (Ed.), Border security & the removal of illegal aliens (pp. 103–183). New York, NY: Nova Science Publishers.

[cl21111-bib-0963] Rathmell, A. , & Valeri, L. (2002). New methods needed to counter cybercrime. Jane's Intelligence Review, 14(8), 52–53.

[cl21111-bib-0964] de Rivera, J. (2005) *Impact of terrorism on emotional climate in the United States*.Poster presented at the annual meeting of the American Psychological Association, Washington, DC.

[cl21111-bib-0965] Roberts, S. K., Jr (2009). Infectious fear: Politics, disease, and the health effects of segregation. Chapel Hill, NC: University of North Carolina Press.

[cl21111-bib-0966] Robinson, N. (2002). New laws seek to balance privacy and surveillance. Jane's Intelligence Review, 14(1), 52–54.

[cl21111-bib-0967] Rodriguez‐Spahia, D. (2018). Gender and terrorism: A homeland security perspective (Doctoral dissertation). Indexed in ProQuest Dissertations and Theses Global database. (ProQuest No. 10936732).

[cl21111-bib-0968] Roed‐Larsen, S. (2006). Public safety investigations of accidents. Paper presented at the annual meeting of the International Sociological Association, Durban, South Africa.

[cl21111-bib-0969] Ronczkowski, M. R. (2006). Terrorism & organized hate crime: Intelligence gathering, analysis & investigation. Boca Raton, FL: CRC Press.

[cl21111-bib-0970] Root, C. (2008). Cooperation or corruption? Informants, cooperating witnesses, snitches and the cops who love/loathe them! Paper presented at the annual meeting of the American Society of Criminology, St. Louis, MO.

[cl21111-bib-0971] Rossi, S. (2003). Busting SPAM. Computerworld, 26(15), 20–25.

[cl21111-bib-0972] Rudofossi, D. (2013). A cop doc's guide to understanding terrorism as human evil: Healing from complex trauma syndromes for military, police, and public safety officers and their families. New York, NY: Baywood Publishing Co.

[cl21111-bib-0973] Rudolph, L. , & Soullez, C. (2007). Les stratégies de la sécurité, 2002‐2007: Avec 150 propositions pour aller plus loin. Paris, France: Presses Universitaires de France.

[cl21111-bib-0974] Runge, J. W. (2002). The role of traffic law enforcement in homeland security. Police Chief, 69(10), 90. 93‐94, 96, 98.

[cl21111-bib-0975] Ruo Men Ke, A. (2008). *Network structure mining and its application in analyzing covert terrorist networks* (Doctoral dissertation). Indexed in ProQuest Dissertations and Theses Global database. (UMI No. H393066)

[cl21111-bib-0976] Sadikovic, L. (2003). Police, democracy and terrorism. Kriminalisticke teme, 2(1‐2), 293–305.

[cl21111-bib-0977] Sakun, S. A. , & Kiselyov, A. V. (2015). Moral and psychological support for the RF Interior Ministry Troops in the North Caucasus. Military Thought, 24(3), 129.

[cl21111-bib-0978] Salter, M. B. (2010). Mapping transatlantic security relations: The EU, Canada, and the War on Terror. New York, NY: Routledge.

[cl21111-bib-0979] Sarkozy, N. (2005). Defending freedom. Défense Nationale et Sécurité Collective, 61(11), 7–18.

[cl21111-bib-0980] Satoh, J. I. (2010). What do the Japanese citizens think of anti‐terrorism legislation in Japan and the United Kingdom from the perspective of human rights? Journal of Osaka Sangyo University: Humanities and Social Sciences, 9, 81–106.

[cl21111-bib-0981] Savinov, L. V. , Dorozhinskaya, E. A. , & Sigarev, A. V. (2015). Examination of information extremist materials: Methodological and legal issues. Criminology Journal of Baikal National University of Economics and Law, 9(2), 209–222.

[cl21111-bib-0982] Serabrakova, I. (2002). Co‐operation of the ministries of internal affairs of the countries of the commonwealth of independent states in combating crime. In M. Pagon (Ed.), Policing in Central and Eastern Europe: Deviance, violence and victimization (pp. 263–270). Ljubljana, Slovenia: College of Police and Security Studies.

[cl21111-bib-0983] Shelley, L. I. (2002). The nexus of organized international criminals and terrorism. International Annals of Criminology, 40(1‐2), 85–92.

[cl21111-bib-0984] Sieges in diplomatic premises . (2002). *Intersec, 12*(2), 45–47.

[cl21111-bib-0985] Simovic, M. (2003). Actions and measures of procedural enforcement and special investigative methods in the criminal procedure act of Bosnia and Herzegovina, in view of fighting organized crime. Kriminalisticke Teme, 2(3‐4), 75–99.

[cl21111-bib-0986] Singh, J. (2002). Inside Indian police. New Delhi, India: Gyan.

[cl21111-bib-0987] Singh, R. (2015). Counter‐terrorism in the post‐9/11 era: Successes, failures and lessons learnt. In R. English (Ed.), Illusions of terrorism and counter‐terrorism (pp. 39–56). Oxford, UK: British Academy.

[cl21111-bib-0988] Singh, R. , & Lasmar, J. M. (2014). Understanding terrorism and counter‐terrorism. New York, NY: Routledge.

[cl21111-bib-0989] Smart guard? (2006) Intersec: The Journal of International Security, 16(4), 26–30.

[cl21111-bib-0990] Sotlar, A. (2002). Coping with extremism within society−The Slovenian experiences. In M. Pagon (Ed.), Policing in Central and Eastern Europe: Deviance, violence and victimization (pp. 593–610). Ljubljana, Slovenia: College of Police and Security Studies.

[cl21111-bib-0991] Stan, C. (2017). Rolul expertizei medico‐legale în investigarea şi analiza actelor de terorism (The role of forensic medical expertise in the investigation and analysis of terrorism acts). Jurnalul Român de Stiințe Criminalistice, 18(5), 2713–2715.

[cl21111-bib-0992] Staniforth, A. (2010). Possession anti‐terrorism training part 90. Jane's Police Review, 118(6072), 27.

[cl21111-bib-0993] Stowe, R. (2007). Co‐ordinating CBRN preparedness in the UK. Crisis Response Journal, 4(1), 40–41.

[cl21111-bib-0994] Strachan, I. (2009). Anti‐terrorism training. Military Technology, 33(12), 57.

[cl21111-bib-0995] Strategies to eradicate international terrorism . (2006). *Security Solutions, 43*(52), 54, 56, 58, 60, 62.

[cl21111-bib-0996] Tahiliani, J. (2009). *An exploratory study of human and social capital and their influence on police confidence in India*. Paper presented at the annual meeting of the American Society of Criminology, St. Louis, MO.

[cl21111-bib-0997] Teague, M. (2009). The radicals among us. Philadelphia Magazine, 100(2), 68.

[cl21111-bib-0998] Tembo, E. B. (2011). Assessing British and American counter‐terrorism: Intelligence, law enforcement and military force (2006‐2009) (Doctoral dissertation). Indexed in ProQuest Dissertations and Theses Global database. (UMI No. U586554).

[cl21111-bib-0999] Terrorism Act gives power to protect . (2003). *Victoria Police Gazette, 10*, 1.

[cl21111-bib-1000] Terrorism and profiling . (2006). *Security Solutions, 41*, 107–108.

[cl21111-bib-1001] Thabouillot, O. , Roffi, R. , Bertho, K. , Ramon, F. , Commeau, D. , Fressancourt, Y. , & Dubourg, O. (2017). Medical causes of temporary or definitive leaves from a French counterterrorist unit pre‐internship. BMJ Military Health, 163, 132–134. 10.1136/jramc-2016-000644 27412359

[cl21111-bib-1002] The first team . (2003). *Jane's Police Review, 111*(5724), 22–28.

[cl21111-bib-1003] The matrix project . (2003). *The Police Chief, 70*(11), 20–21.

[cl21111-bib-1004] The USA Patriot Act . (2003). Crime and Justice International: Worldwide News and Trends, 19(69), 27.

[cl21111-bib-1005] Thickening the thinning blue line . (2002). *Association News*, 6–7

[cl21111-bib-1006] Thirty years of international cooperation . (2003). *AFP News: Newsletter of the Australian Federal Police, 113*, 7–8.

[cl21111-bib-1007] Thomas, M. (2003). Study group workshops bring IT industry and law enforcement together. The Police Chief, 70(4), 14.

[cl21111-bib-1008] Tigner, B. (2007). *EU counterterrorism: Evolving complexities*. Zürich, Switzerland: Center for Security Studies and Conflict Research, Swiss Federal Institute of Technology.

[cl21111-bib-1009] Tiron, R. (2009). Human intelligence. Sea Power, 52(4), 28.

[cl21111-bib-1010] Torture: The true purpose . (2005). *Human Rights Defender, 24*(4), 5–6.

[cl21111-bib-1011] Treverton, G. F. (2009). Intelligence for an age of terror. Cambridge, NY: Cambridge University Press.

[cl21111-bib-1012] Ulrich, C. J. , & Kivimäki, T. (2002). Uncertain security: Confronting transnational crime in the Baltic Sea region & Russia. Oxford, UK: Lexington Books.

[cl21111-bib-1013] Under surveillance . (2003). *Jane's Police Review, 111*(5709), 30–31.

[cl21111-bib-1014] Uyaksil, A. (2006). *Bir halkla iliskiler uygulamasi olarak toplum destekli polislik modeli nd [Community policing as public relations]*. Paper presented at the National Public Relations Symposium, Kocaeli University, Kocaeli, Turkey.

[cl21111-bib-1015] Verdugo, C. (2008). International standards in anti‐money laundering and combating the terrorist financing regulation: Compliance and strategy changes. Global Business and Economics Review, 10(3), 353–378. 10.1504/GBER.2008.019988

[cl21111-bib-1016] Vermeulen, F. , & Bovenkerk, F. (2012). Engaging with violent Islamic extremism: Local policies in Western European cities. The Hague, The Netherlands: Eleven International Publishers.

[cl21111-bib-1017] Vgontzas, A. N. (2008). War on terrorism: From the bloody clash of civilisations to a nonviolent coexistence of nations and civilisations. Global Business and Economics Review, 10(2), 211–215. 10.1504/GBER.2008.019020

[cl21111-bib-1018] Von Brakle, M. (2007). *From radicalization to reconciliation: Political violence and terrorism during the transition to democracy*. Paper presented at the annual meeting of the American Society of Criminology, Atlanta, GA.

[cl21111-bib-1019] Walker, C. (2009, November). *Conscripting the public in terrorism policing: Towards safer communities or a police state?* Paper presented at the 61st annual meeting of the American Society of Criminology, Philadelphia, PA.

[cl21111-bib-1020] Walter, A. A. (2004). First responder handbook. Clifton Park, NY: Thomson/Delmar Learning.

[cl21111-bib-1021] Walzer, M. (2005). Was ist falsch am Terrorismus? [What is wrong with terrorist tactics?]. Mittelweg, 36, 13(6), 73–86.

[cl21111-bib-1022] Wan, J. (2002). *On international terrorist crime* (Doctoral dissertation). Indexed in ProQuest Dissertations and Theses Global database. (UMI No. 1024997179).

[cl21111-bib-1023] Waseem, H. , Razzak, J. A. , Naseer, R. , & Mehmood, A. (2010). Pre‐hospital management in terrorist MCI's in Punjab, Pakistan year 2009. Injury Prevention, 16, A196. 10.1136/ip.2010.029215.699

[cl21111-bib-1024] Water police . (2005). *Security Electronics Magazine*, 253, 56–57.

[cl21111-bib-1025] Watts, S. D. (2008). *Report on hate crime in Canada: The Royal Canadian Mounted Police perspective*. Ottawa, ON, Canada: Royal Canadian Mounted Police.

[cl21111-bib-1026] Werner, E. , Lhomme, M. , & de Benoist, A. (2005). Nous sommes tous en liberté surveillée [We all are under monitored freedom]. Élements pour la Civilisation Européenne, 118, 27–44.

[cl21111-bib-1027] Wexler, S. (2002). The NYPD: On the front lines in the war against terrorism. Law Enforcement Technology, 76‐80, 82–84.

[cl21111-bib-1028] Wexler, S. (2003). The Philadelphia story: Philly's police department is applying innovative policing strategies to reduce crime and combat terrorism. Law Enforcement Technology, 30(10), 10–16.

[cl21111-bib-1029] Wheatley, S. , Hollingsworth, A. C. , & Greaves, I. (2018). Responding to the marauding terror attack: The police perspective. Journal of the Royal Army Medical Corps, 166(2), 80–83. 10.1136/jramc-2018-000960 30012666

[cl21111-bib-1030] White, J. R. (2004). Defending the homeland: Domestic intelligence, law enforcement, and security. Toronto, ON, Canada: Wadsworth/Thomson Learning.

[cl21111-bib-1031] Wilczak, C. (2006). *Consultant feedback on studying 9/11 New York City Police trauma*. Paper presented at the American Psychological Association 114th Annual Convention, New Orleans, LA.

[cl21111-bib-1032] Williams, D. (2003). *Formal opening address*. Paper presented at The 2003 Homeland Security Conference ‐ Safeguarding Australia: Frontline Issues Conference, National Convention Centre, Canberra, ACT, Australia.

[cl21111-bib-1033] Williams, J. E. G. , Bulthouse, J. M. , Ferralez, C. , Siddle., K. M. , & Siddle, B. K. (2008). *Biopsychological responding of police in extreme stress firearms/hostage simulation events*. Paper presented at the Eightieth Annual Meeting of the Midwestern Psychological Association, Chicago, IL.

[cl21111-bib-1034] Wilson, R. (2011). *Blue fish in a dark sea: Police intelligence in a counterinsurgency* (Doctoral dissertation). Indexed in ProQuest Dissertations and Theses Global database. (UMI No. U604868).

[cl21111-bib-1035] Withrow, B. L. (2006). Racial profiling: From rhetoric to reason. Upper Saddle River, NJ: Pearson/Prentice Hall.

[cl21111-bib-1036] Zhao, C. Y. (2002). *On regional criminal law* (Doctoral dissertation). Indexed in ProQuest Dissertations and Theses Global database. (UMI No. 1024954184).

[cl21111-bib-1037] Zimmering, R. (2005). Mexico: A revolutionary army becomes police force. Welt Trends, 49, 60–72.

[cl21111-bib-1038] Aman, M. (2007). Preventing terrorist suicide attacks. Mississauga, ON, Canada: Jones and Bartlett.

[cl21111-bib-1039] Barton, G. (2011). *Indonesia's counterterrorism challenge: Dealing with radicalization*. Paper presented at the GTReC ARC Linkage Project on Radicalisation Conference 2011 — Lessons from 9/11: Reflections on the Evolution of Counter Terrorism Policy, Practice and Analysis in the Decade Since the Attacks of September 11, 2001, Melbourne, VIC, Australia.

[cl21111-bib-1040] Barton, G. (2012). *Countering violent extremism in the Indonesian context: The key elements of an evolving CVE program*. Paper presented at the GTReC ARC Linkage Project on Radicalisation Conference 2012 — Terrorism and Counter‐terrorism in Australia and Indonesia: 10 years after Bali, Melbourne, VIC, Australia.

[cl21111-bib-1041] Bebbington, S. (2008). Terrorism specs. Jane's Police Review, 116(5967), 22–25.

[cl21111-bib-1042] Bermudez, J. S. (2003). North Korea prepares for digital war. Jane's Intelligence Review, 15(10), 32–37.

[cl21111-bib-1043] Burrell, W. D. (2002). *Leadership in a time of crisis: A guide for managers*. Community Corrections Report on Law and Corrections Practice, 9(2), 17–29.

[cl21111-bib-1044] Caspersen, S. J. (2003). New Jersey's office of counter‐terrorism: One state's efforts to detect and deter terrorism. Journal of Counterterrorism & Homeland Security International, 9(4), 8–12.

[cl21111-bib-1045] Cavailles, C. (2005). Quelle politique Europeenne contre le terrorisme? Revue du Marche Commun et de l'Union Europeenne, 489, 382–384.

[cl21111-bib-1046] Chalk, P. (2006). Australian mass transport vulnerable to attack. Homeland Security and Resilience Monitor, 5(6), 16–17.

[cl21111-bib-1047] Churchill, B. W. (2008). *Transportation security and the FBI's infragard program*. Paper presented at the 15th World Congress on Intelligent Transport Systems and ITS America Annual Meeting, New York City, NY.

[cl21111-bib-1048] Das, D. K. & Jiao, A. Y. (Eds.). (2005). Public order: A global perspective. Upper Saddle River, NJ: Pearson Prentice Hall.

[cl21111-bib-1049] Davis, A. (2003). Thailand faces up to southern extremist threat. Jane's Intelligence Review, 15(10), 12–15.

[cl21111-bib-1050] McDermott, R. N. , & O Malley, W. D. (2003). Countering terrorism in Central Asia. Jane's Intelligence Review, 15(10), 16–19.

[cl21111-bib-1051] Defeating terrorism . (2002). *Police Chief, 69*(2), 19–70.

[cl21111-bib-1052] Delord, R. G. (2005). Should Australia mirror US labor and policing models? Association News: The Official Journal of the Police Association of Tasmania, 9(3), 57–60.

[cl21111-bib-1053] Ghaffur, T. (2002). Policing the backlash. Police Review, 110(5659), 20–21.

[cl21111-bib-1054] Government's four‐year plan for policing: Community safety and smarter policing form the basis of a four‐year State Government blueprint for Victoria Police . (2003). *Police Life: Victoria Police Magazine*, 15.

[cl21111-bib-1055] Gozubenli, M. , & Akbas, H. (2009). The implementation of community‐oriented policing as a part of establishing democratic policing in Eastern Anatolia: The benefits and challenges. In I. L. Gultekin , I. Lofka , C. Baycan , M. Delice & F. Goktepe (Eds.), Global and regional perspectives on democracy and security (pp. 78–92). Washington, DC: TISD.

[cl21111-bib-1056] Gozubenli, M. , Ozturk, M. , & Ozgenturk, I. (2008). *A case study on implementing community policing in Eastern Anatolia Region of Turkey*. Paper presented at the annual meeting of the American Society of Criminology, St. Louis, MO.

[cl21111-bib-1057] Gregory, F. (2007). Police and counter‐terrorism in the UK: A study of 'one of the highest and most challenging priorities for police forces nationally'. In P. Wilkinson (Ed.), Homeland security in the UK: Future preparedness for terrorist attack since 9/11 (pp. 200–245). London, UK: Routledge.

[cl21111-bib-1058] Gregory, P. (2012). *The arts & crafts: what's different about 21st century intelligence?* Paper presented at the GTReC ARC Linkage Project on Radicalisation Conference 2012−Terrorism and Counter‐terrorism in Australia and Indonesia: 10 years after Bali, Melbourne, VIC, Australia.

[cl21111-bib-1059] Hanson, D. (2004). The nation's food and water supply: A new target for terrorists? Law Enforcement Technology, 31(1), 18–23.

[cl21111-bib-1060] Harris‐Hogan, S. (2013). *Combating radicalisation in Australia*. Paper presented at the 2013 National Metropolis Conference: Building an Integrated Society, Ottawa, ON, Canada.

[cl21111-bib-1061] Harwood, M. (2008). Fighting terrorism in the U.K. (cover story). Security Management, 52(1), 48–55.

[cl21111-bib-1062] Haynes, C. (2008). Eyes wide open. Jane's Police Review, 116(5977), 18–19.

[cl21111-bib-1063] Hodgson, J. F. & Orban, C. (Eds.). (2005). Public policing in the 21st century: Issues and dilemmas in the U.S. and Canada. Monsey, NY: Criminal Justice Press.

[cl21111-bib-1064] Jones, K. (2005). Confronting terrorism: The need for immediate changes to law and practice. Jane's Police Review, 113(5), 16–17.

[cl21111-bib-1065] Jones, B. (2006). Renewing reform. Policing Today, 12(3), 17–18.

[cl21111-bib-1066] Kemp, R. (2003). Homeland security: Best practices for local government. Washington, DC: ICMA.

[cl21111-bib-1067] Lambert, R. (2010). *The London partnerships: An insider's analysis of legitimacy and effectiveness* (Doctoral dissertation). University of Exeter, Exeter, UK.

[cl21111-bib-1068] Lambert, R. (2011). Countering Al‐Qaeda in London: Police and Muslims in partnership. London, UK: Hurst & Company.

[cl21111-bib-1069] Lee, J. (2007). *Has community policing run its course? Community policing implementation after 9/11*. Paper presented at the annual meeting of the American Society of Criminology, Atlanta, GA.

[cl21111-bib-1070] Leivesley, S. (2008). Downsizing terror recruitment. The Journal of International Security, 18(6), 34–36.

[cl21111-bib-1071] Lentini, P. (2004). What has changed, and what hasn't changed, since 9/11? In M. Vicziany , D. P. Wright‐Neville & P. Lentini (Eds.), Regional security in the Asia Pacific–9/11 and after (pp. 2–29). Cheltenham, UK: Edward Elgar Publishing.

[cl21111-bib-1072] Lentini, P. (2012. *Grace under pressure? Assessing the impact of surveillance and counter terrorism raids on terrorist behaviour: A study based on wire‐tap and electronic bugging device evidence of a Melbourne Jema'ah 2004‐5*. Paper presented at the GTReC ARC Linkage Project on Radicalisation Conference 2012 — Terrorism and Counter‐terrorism in Australia and Indonesia: 10 years after Bali, Melbourne,VOC, Australia.

[cl21111-bib-1073] Lewindon, M. (2006). Russian roulette: It is Russia's turn to host the G8 Summit. Jane's Police Review, 114(5885), 22–23.

[cl21111-bib-1074] Lieberman, C. (2008). *Is community policing the future of counter‐terrorism?* Paper presented at the annual meeting of the American Society of Criminology, St Louis, MO.

[cl21111-bib-1075] Lieberman, C. (2009). *Counter‐terrorism through community engagement*. Paper presented at the American Society of Criminology Annual Meeting, Philadelphia, PA.

[cl21111-bib-1076] Lining up a security goal . (2003). *New South Wales Police News: Official Journal of the Police Association of New South Wales, 83*(11), 21, 23, 25.

[cl21111-bib-1077] Mencer, C. , Lynch, M. , & Allison, J. (2005). The new era of campus public safety. Campus Law Enforcement Journal, 35(2), 21–25.

[cl21111-bib-1078] Northern region shows its true colours . (2003). *AFP News: Newsletter of the Australian Federal Police*, 113, 24‐25.

[cl21111-bib-1079] Occhipinti, J. (2003). The politics of EU police cooperation: Toward a European FBI? Boulder, CO: L. Rienner.

[cl21111-bib-1080] Olin, W. (2003). Why traditional law enforcement methods cannot win the war on terrorism. Crime & Justice International, 19(77), 19–22.

[cl21111-bib-1081] Oliver, W. (2004). The homeland security juggernaut: The end of the community policing era? Crime & Justice International, 20(79), 4–10.

[cl21111-bib-1082] Peed, C. (2002a). Director's column.

[cl21111-bib-1083] Peed, C. (2002b). *Director's notes*.

[cl21111-bib-1084] Plywaczewski, E. (2009). *New ways of response to transnational crime: Central European police academy as an example*. Paper presented at the American Society of Criminology Annual Meeting, Philadelphia, PA.

[cl21111-bib-1085] Picarelli, J. , Horgan, J. , Schanzer, D. H. , & Downing, M. (2016). *Community‐level efforts to prevent violent extremism [transcript]*. Washington, DC: National Institute of Justice.

[cl21111-bib-1086] Policing Mother Nature the RCMP's role in natural disasters (2002). [Treatment with human chorion‐gonadotropin (pregnyl) of patients with vaginal bleeding in the first trimester of pregnancy]. Royal Canadian Mounted Police Gazette, 64(4), 13–15.

[cl21111-bib-1087] Russo, J. , & Labriola, D. (2002). Local information sharing systems fight terrorism. The Police Chief, 69(7), 68–70.

[cl21111-bib-1088] Schwarz, R. , & Colussi, M. (2015). Averting of dangers by the police in cases of international terrorism: Thoughts on the legal assessment of police measures according to § 20k BKAG. Kriminalistik, 69(3), 199–201.

[cl21111-bib-1089] Sending our expertise overseas . (2003). *AFP News: Newsletter of the Australian Federal Police*, 113, 20–21.

[cl21111-bib-1090] Sevinç, A. , & Demir, O. Ö. (2010). Problem Odaklı polislik yaklaşımının. In M. A. Sözer (Ed.), Toplum destekli polislikteki yeri. Toplum: Suç ve Güvenlik. Ankara, Turkey: Adalet Yayınevi.

[cl21111-bib-1091] Shusta, R. M. (2011). Multicultural law enforcement: Strategies for peacekeeping in a diverse society. Boston, MA: Prentice Hall.

[cl21111-bib-1092] Smith, D. (2012). *Countering violent extremism: An emotion centred approach*. Paper presented at the GTReC ARC Linkage Project on Radicalisation Conference 2012 — Terrorism and Counter‐terrorism in Australia and Indonesia: 10 years after Bali, Melbourne, VIC, Australia.

[cl21111-bib-1093] Spalek, B. (2015). Community engagement for counter terrorism in Britain: An exploration of the role of connectors in countering Takfiri Jihadist terrorism. Current, 571, 13.

[cl21111-bib-1094] Staniforth, A. (2008a). Characteristics anti‐terrorism training part 31. Jane's Police Review, 116(6007), 39.

[cl21111-bib-1095] Staniforth, A. (2008b). Know your enemy: Tackling terrorism. Part 1 of 10‐part series. Jane's Police Review, 116(5967), 26–27.

[cl21111-bib-1096] Staniforth, A. (2008c). Membership anti terrorism training part 5. Jane's Police Review, 116(5981), 35.

[cl21111-bib-1097] Staniforth, A. (2008d). Methods of mayhem: Tackling terrorism. Part 7 of 10‐part series. Jane's Police Review, 116(5973), 30–31.

[cl21111-bib-1098] Staniforth, A. (2008e). Munich massacre. Jane's Police Review, 116(5992), 30–33.

[cl21111-bib-1099] Staniforth, A. (2008f). Nationalism anti‐terrorism training part 17. Jane's Police Review, 116(5993), 43.

[cl21111-bib-1100] Staniforth, A. (2008g). Police reform anti‐terrorism training part 25. Jane's Police Review, 116(6001), 41.

[cl21111-bib-1101] Staniforth, A. (2008h). Pursuit anti terrorism training part 9. Jane's Police Review, 116(5985), 40.

[cl21111-bib-1102] Staniforth, A. (2008i). Suicide attack anti‐terrorism training part 18. Jane's Police Review, 116(5994), 37.

[cl21111-bib-1103] Staniforth, A. (2008j). Team effort: Tackling terrorism. Part 3 of 10‐part series. Jane's Police Review, 116(5969), 28–31.

[cl21111-bib-1104] Staniforth, A. (2008k). UK security anti‐terrorism training part 26. Jane's Police Review, 116(6002), 39.

[cl21111-bib-1105] Staniforth, A. (2009a). Afghanistan anti‐terrorism training part 81. Jane's Police Review, 117(6057), 32–33.

[cl21111-bib-1106] Staniforth, A. (2009b). Data sharing anti‐terrorism training part 36. Jane's Police Review, 117(6012), 34–35.

[cl21111-bib-1107] Staniforth, A. (2009c). Impact anti‐terrorism training part 46. Jane's Police Review, 117(6022), 35.

[cl21111-bib-1108] Staniforth, A. (2009d). Prepare anti‐terrorism training part 57. Jane's Police Review, 117(6033), 35.

[cl21111-bib-1109] Staniforth, A. (2009e). Tangled web. Jane's Police Review, 117(6017), 28–29.

[cl21111-bib-1110] Staniforth, A. (2009f). Toxic terrorists: Anti‐terrorism training part 74. Jane's Police Review, 117(6050), 31.

[cl21111-bib-1111] Staniforth, A. (2014). Preventing terrorism and violent extremism. Oxford, UK: Oxford University Press.

[cl21111-bib-1112] Staun, J. (2008). Radicalisation, recruitment and the EU counter‐radicalisation strategy. The Hague, The Netherlands: COT Institute for Safety, Security and Crisis Management.

[cl21111-bib-1113] Stegenga, P. (2003). Meeting the challenge: One neighborhood watch at a time. *The Police Chief, 70*(1), 55–56.

[cl21111-bib-1114] Strategic targeting and prioritization: A pro‐active approach to targeting criminal activity . (2002). *Intersec, 12*(5), 161–163.

[cl21111-bib-1115] Studdert, M. (2007). National security hotline and public information campaign. Security Solutions, 45(79‐80), 82.

[cl21111-bib-1116] Tucker, D. (2006). Winning back trust. Jane's Police Review, 114(5882), 18–19.

[cl21111-bib-1117] Tudor, R. (2003). Saddam loyalists pose biggest threat to Iraqi security. Jane's Intelligence Review, 15(10), 40–41.

[cl21111-bib-1118] Vicziany, M. , Wright‐Neville, D. P. & Lentini, P. (Eds.). (2004). Regional security in the Asia Pacific–9/11. Cheltenham, UK: Edward Elgar Publishing.

[cl21111-bib-1119] Warnes, R. J. (2011). The importance of local community knowledge in policing terrorism. In S. Ekici (Ed.), NATO science for peace and security series ‐ E: Human and societal dynamics (pp. 217–231). Berlin, Germany: IOS Press.

[cl21111-bib-1120] Warnings, indicators and terrorism . (2004). *Policing Issues and Practice Journal, 12*(4), 36–41.

[cl21111-bib-1121] Weitz, R. (2007). Joint Terrorism Task Forces: A users' manual. Homeland Security and Resilience Monitor, 6(10), 10–11.

[cl21111-bib-1122] Weston, K. (2007). Breaking the cycle. Intersec, 17(1), 36–38.

[cl21111-bib-1123] What police chiefs can do about terrorism ‐‐ Now! . (2002). *Police Chief, 69*(2), 19‐22, 25, 28‐33.

[cl21111-bib-1124] Williams, B. N. , Brodt, E. , & Schiller, T. (2008). Bringing theoretical collaboration into practice: Community policing, Homeland Security and the Police Training Officer (PTO) Model. *Proceedings of the Fourth International Conference on Public Administration*, 1, 3–15.

[cl21111-bib-1125] Yates, S. (2009). The crime fighters' Odyssey. Policing Today, 15(5), 18–19.

[cl21111-bib-1126] Barker, C. (2015). *Australian Government measures to counter violent extremism: A quick guide*. Retrieved from https://parlinfo.aph.gov.au/parlInfo/download/library/prspub/3650900/upload_binary/3650900.pdf;fileType=application/pdf#search=%22library/prspub/3650900%22

[cl21111-bib-1127] Barrelle, K. (2015). Pro‐integration: disengagement from and life after extremism. Behavioural Sciences of Terrorism and Political Aggression, 7, 129–142. 10.1080/19434472.2014.988165

[cl21111-bib-1128] Bradford, B. , Murphy, K. , & Jackson, J. (2014). Officers as mirrors: Policing, procedural justice and the (re) production of social identity. British Journal of Criminology, 54(4), 527–550. 10.1093/bjc/azu021

[cl21111-bib-1129] Berger, J. M. (2016). *Making CVE work*. International Centre for Counter‐Terrorism. The Hague, The Netherlands, 7. 10.19165/2016.1.05

[cl21111-bib-1130] Cherney, A. (2018). Police community engagement and outreach in a counterterrorism context. Journal of Policing, Intelligence and Counter Terrorism, 13, 60–79. 10.1080/18335330.2018.1432880

[cl21111-bib-1131] Cherney, A. , & Belton, E. (2019a). Assessing intervention outcomes targeting radicalised offenders: Testing the pro‐integration model of extremist disengagement as an evaluation tool. Dynamics of Asymmetric Conflict. Advance online publication. 10.1080/17467586.2019.1680854

[cl21111-bib-1132] Cherney, A. , & Belton, E. (2019b). Evaluating case‐managed approaches to counter radicalization and violent extremism: An example of the proactive integrated support model (PRISM) intervention. Studies in Conflict and Terrorism. Advance online publication. 10.1080/1057610X.2019.1577016

[cl21111-bib-1133] Cherney, A. , & Hartley, J. (2017). Community engagement to tackle terrorism and violent extremism: Challenges, tensions and pitfalls. Policing and Society, 27, 750–763. 10.1080/10439463.2015.1089871

[cl21111-bib-1134] Cherney, A. , & Murphy, K. (2017). Police and community cooperation in counterterrorism: Evidence and insights from Australia. Studies in Conflict & Terrorism, 40(12), 1023–1037. 10.1080/1057610X.2016.1253987

[cl21111-bib-1135] Cherney, A. , Bell, J. , Leslie, E. , Cherney, L. , & Mazerolle, L. (2018). *Countering violent extremism evaluation indicator document*. Australian and New Zealand Counter‐Terrorism Committee, National Countering Violent Extremism Evaluation Framework and Guide. Brisbane, QLD, Australia: University of Queensland.

[cl21111-bib-1136] De Cremer, D. , & Sedikides, C. (2005). Self‐uncertainty and responsiveness to procedural justice. Journal of Experimental Social Psychology, 41(2), 157–173. 10.1016/j.jesp.2004.06.010

[cl21111-bib-1137] Donner, C. , Maskaly, J. , Fridell, L. , & Jennings, W. G. (2015). Policing and procedural justice: A state‐of‐the‐art review. Policing: An International Journal of Police Strategies & Management, 38(1), 153–172. 10.1108/PIJPSM-12-2014-0129

[cl21111-bib-1138] Farrington, D. P. (2003). Methodological quality standards for evaluation research. The Annals of the American Academy of Political and Social Science, 587, 49–68. 10.1177/0002716202250789

[cl21111-bib-1139] Fox, J. P. (2012). *Legitimacy and law enforcement: The counterinsurgency against gang crime in the United States* (Doctoral dissertation). Georgetown University, Washington, DC.

[cl21111-bib-1140] Gill, C. , Weisburd, D. , Telep, C. W. , Vitter, Z. , & Bennett, T. (2014). Community‐oriented policing to reduce crime, disorder and fear and increase satisfaction and legitimacy among citizens: A systematic review. Journal of Experimental Criminology, 10, 399–428. 10.1007/s11292-014-9210-y

[cl21111-bib-1141] Glass, G. V. (1997). Interrupted time series quasi‐experiments. In R. M. Jaeger (Ed.), Complementary methods for research in education (2nd ed., pp. 589–608). Washington DC: American Educational Research Association.

[cl21111-bib-1142] Grossman, M. , Peucker, M. , Smith, D. , & Dellal, H. (2016). *Stocktake research project: A systematic literature and selected program review on social cohesion, community resilience and violent extremism 2011–2015*. Melbourne, VIC, Australia: Community Resilience Unit, Department of Premier and Cabinet.

[cl21111-bib-1143] Grossman, M. , Stephenson, P. , Street, R. , & Zhang, G. (2015). *Community reporting thresholds: Sharing information with authorities concerning violent extremist activity and involvement in foreign conflict*. Canberra, Australia: Countering Violent Extremism Subcommittee, Australia‐New Zealand Counter‐Terrorism Committee. Retrieved from https://www.researchgate.net/publication/330701084_Community_Reporting_Thresholds_Sharing_information_with_authorities_concerning_violent_extremist_activity_and_involvement_in_foreign_conflict

[cl21111-bib-1144] Government Offices of Sweden . (2011). *Action plan to safeguard democracy against violence‐promoting extremism*. Retrieved from https://www.government.se/contentassets/b94f163a3c5941aebaeb78174ea27a29/action-plan-to-safeguard-democracy-against-violence-promoting-extremism-skr.-20111244

[cl21111-bib-1145] Higginson, A. , Eggins, E. , Mazerolle, L. , & Stanko, E. (2015). *The Global Policing Database* [Database and Protocol]. Retrieved from http://www.gpd.uq.edu.au

[cl21111-bib-1146] Higginson, A. , & Neville, R. (2014). *SysReview [systematic review management software]*. Brisbane, QLD, Australia: The University of Queensland.

[cl21111-bib-1147] Holdaway, L. , & Simpson, R. (2018). *Improving the impact of preventing violent extremism programming. A toolkit for design, monitoring and evaluation*. International Alert; UNDP, 1–136. Retrieved from www.undp.org/content/dam/norway/undp-ogc/documents/PVE_ImprovingImpactProgrammingToolkit_2018.pdf

[cl21111-bib-1148] Hofman, J. & Sutherland, A. (Eds.). (2018). Evaluating interventions that prevent or counter violent extremism: A practical guide. Santa Monica, CA: RAND Corporation.

[cl21111-bib-1149] Horgan, J. (2009). Individual disengagement: A psychological analysis. In T. Bjorgo & J. Horgan (Eds.), Leaving terrorism behind: Individual and collective disengagement (pp. 35–47). New York, NY: Routledge.

[cl21111-bib-1150] Horgan, J. , & Braddock, K. (2010). Rehabilitating the terrorists? Challenges in assessing the effectiveness of de‐radicalization programs. Terrorism and Political Violence, 22, 267–291. 10.1080/09546551003594748

[cl21111-bib-1151] Huo, Y. J. , Smith, H. J. , Tyler, T. R. , & Lind, E. A. (1996). Superordinate identification, subgroup identification, and justice concerns: Is separatism the problem; is assimilation the answer? Psychological Science, 7(1), 40–45. 10.1111/j.1467-9280.1996.tb00664.x

[cl21111-bib-1152] Jackson, J. , Hough, M. , Bradford, B. , & Kuha, J. (2015). Empirical legitimacy as two connected psychological states. In I. G. Meško & J. Tankebe J . (Eds.), Trust and legitimacy in criminal justice (pp. 137–160). Cham, Switzerland: Springer. 10.1007/978-3-319-09813-5_7

[cl21111-bib-1153] Khalil, J. , & Zeuthen, M. (2016). *Countering violent extremism and risk reduction: A guide to program design and evaluation*. London, UK: Royal United Services Institute for Defence and Security Services.

[cl21111-bib-1154] Klausen, J. , Campion, S. , Needle, N. , Nguyen, G. , & Libretti, R. (2016). Toward a behavioural model of “homegrown” radicalization trajectories. Studies in Conflict & Terrorism, 39(1), 67–83. 10.1080/1057610X.2015.1099995

[cl21111-bib-1155] Koehler, D. (2017). *Preventing violent radicalization: Programme design and evaluation*. Retrieved from https://www.cidob.org/en/articulos/monografias/resilient_cities/preventing_violent_radicalisation_programme_design_and_evaluation

[cl21111-bib-1156] Lowe, D. (2017). Prevent strategies: The problems associated in defining extremism: The case of the United Kingdom. Studies in Conflict & Terrorism, 40(11), 917–933. 10.1080/1057610X.2016.1253941

[cl21111-bib-1182] Mazerolle, L. , Cherney, A. , Eggins, E. , Higginson, A. , Hine, L. , & Belton, E. (2020). PROTOCOL: Police programs that seek to increase community connectedness for reducing violent extremism behaviour, attitudes and beliefs. Campbell Systematic Reviews, 16, e1076. 10.1002/cl2.1076 PMC8356312 37133272

[cl21111-bib-1157] Mazerolle, L. , Sargeant, E. , Cherney, A. , Bennett, S. , Murphy, K. , Antrobus, E. , & Martin, P. (2014). Procedural justice and legitimacy in policing. Cham, Switzerland: Springer. 10.1007/978-3-319-04543-6

[cl21111-bib-1158] Meares, T. L. , & Tyler, T. R. (2020). *The battle for the constitution: The first step is figuring out what police are for*. The Atlantic. Retrieved from https://www.theatlantic.com/ideas/archive/2020/06/first-step-figuring-out-what-police-are/612793/

[cl21111-bib-1159] Murphy, K. (2013). Policing at the margins: Fostering trust and cooperation among ethnic minority groups. Journal of Policing, Intelligence and Counter Terrorism, 8(2), 184–199. 10.1080/18335330.2013.821733

[cl21111-bib-1160] Murray, A. , Mueller‐Johnson, K. , & Sherman, L. (2015). Evidence‐based policing of U.K. Muslim communities: Linking confidence in the police with area vulnerability to violent extremism. International Criminal Justice Review, 25, 64–79. 10.1080/15614260500293986

[cl21111-bib-1161] Nagin, D. S. , & Telep, C. W. (2017). Response to “Procedural justice and policing: A rush to judgment?”. Annual Review of Law and Social Science, 13, 55–58. 10.1146/annurev-lawsocsci-120516-024409

[cl21111-bib-1162] National Consortium of the Study of Terrorism and Responses to Terrorism (START) . (2018). *Profiles of individual in the United States (PIRUS) Codebook*. [Codebook]. Retrieved from https://www.start.umd.edu/sites/default/files/files/research/PIRUSCodebook.pdf

[cl21111-bib-1163] Norwegian Ministry of Justice and Public Security (2014). *Action plan against radicalization and violent extremism* (p. 7). Oslo, Norway: Norwegian Ministry of Justice and Public Security.

[cl21111-bib-1164] Perliger, A. , & Pedahzur, A. (2011). Social network analysis in the study of terrorism and political violence. Political Science & Politics, 44, 45–50. 10.1017/S10490965100001848

[cl21111-bib-1165] Pickering, S. , McCulloch, J. , & Wright‐Neville, D. (2008). Counter terrorism policing: Community, cohesion and security. New York, NY: Springer.

[cl21111-bib-1166] Public Safety Canada . (2018). *National strategy on countering radicalization to violence*. Retrieved from https://www.publicsafety.gc.ca/cnt/rsrcs/pblctns/ntnl-strtg-cntrng-rdclztn-vlnc/ntnl-strtg-cntrng-rdclztn-vlnc-en.pdf

[cl21111-bib-1167] Ramiriz, D. , Quinlan, T. L. , Malloy, S. P. , & Shutt, T. (2013). Community partnerships thwart terrorism. In D. P. Silk , B. Spalek & M. O'Rawe (Eds.), Preventing ideological violence: Communities, police and case studies of success (pp. 151–169). New York, NY: Palgrave Macmillan.

[cl21111-bib-1168] Romaniuk, P. , & Chowdhury Fink, N. (2012). *From input to impact: Evaluating terrorism prevention programs*. New York, NY: Center on Global Counterterrorism Cooperation.

[cl21111-bib-1169] San, S. (2018). Counter‐terrorism policing innovations in Turkey: A case study of Turkish National Police CVE experiment. Policing and Society, 28, 1–16. 10.1080/10439463.2018.1561697

[cl21111-bib-1170] Schanzer, D. , Kurzman, C. , Toliver, J. , & Miller, E. (2016). *Challenge and promise of using community policing strategies to prevent violent extremism: A call for community partnerships with law enforcement to enhance public safety*. Washington, DC: National Institute of Justice, US Department of Justice.

[cl21111-bib-1171] Shadish, W. R. , Cook, T. D. , & Campbell, D. T. (2002). Experimental and quasi‐experimental designs for generalized causal inference. Boston, MA: Houghton Mifflin.

[cl21111-bib-1172] Sterne, J. A. , Hernán, M. A. , Reeves, B. C. , Savović, J. , Berkman, N. D. , Viswanathan, M. , … Carpenter, J. R. (2016). ROBINS‐I: A tool for assessing risk of bias in non‐randomised studies of interventions. BMJ, 355, i4919. 10.1136/bmj.i4919 27733354 PMC5062054

[cl21111-bib-1173] Thomas, K. T. (2019). Bridging social boundaries and building social connectedness: Through youth development programs. Equality, Diversity and Inclusion: An International Journal. Advance online publication. 10.1108/EDI-02-2018-0019

[cl21111-bib-1174] Thomas, P. , Grossman, M. , Miah, S. , & Christmann, K. (2017). *Community reporting thresholds: Sharing information with authorities concerning violent extremist activity and involvement in foreign conflict: A UK replication study*. Centre for Research and Evidence on Security Threats. Retrieved from http://eprints.hud.ac.uk/id/eprint/33161/1/Community%20Reporting%20Thresholds%20UK%20Final%20Report.pdf

[cl21111-bib-1175] Tyler, T. R. (2004). Enhancing police legitimacy. The Annals of the American Academy of Political & Social Science, 593(1), 84–99. 10.1177/0002716203262627

[cl21111-bib-1176] Tyler, T. (2017). Procedural justice and policing: A rush to judgment? Annual Review of Law and Social Science, 13, 29–53. 10.1146/annurev-lawsocsci-110316-113318

[cl21111-bib-1177] Tyler, T. R. , Goff, P. A. , & MacCoun, R. J. (2015). The impact of psychological science on policing in the United States: Procedural justice, legitimacy, and effective law enforcement. Psychological Science in the Public Interest, 16(3), 75–109. 10.1177/1529100615617791 26635334

[cl21111-bib-1178] Tyler, T. R. , & Lind, E. A. (1992). A relational model of authority in groups. In M. P. Zanna (Ed.), Advances in experimental social psychology (25, pp. 115–191). New York, NY: Academic Press. 10.1016/S0065-2601(08)60283-X

[cl21111-bib-1179] Van Den Bos, K. (2018). Why people radicalize: How unfairness judgments are used to fuel radical beliefs, extremist behaviours, and terrorism. New York, NY: Oxford University Press.

