# Supplementary material for: Police programmes that seek to increase community connectedness for reducing violent extremism behaviour, attitudes and beliefs
Source: Campbell Syst Rev. 2020 Sep 8;16(3):e1111. doi: 10.1002/cl2.1111 (PMC8356323; doi:10.1002/cl2.1111)
Supplement: Supplementary file 1 — Supporting information [file CL2-16-e1111-s001.docx]

## Appendix A: Systematic search record

Table A1: Grey literature search record

| **Source** | **Search date** | **Search strategy** | **N results** |
| --- | --- | --- | --- |
| *Global Terrorism Research Centre (Monash University)* | 11/02/2020 | No search functionality. Hand-searched the publications section of the website for publications mentioning police and/or a response to terrorism/radicalisation/extremism. | 21 |
| *Triangle Centre on Terrorism and Homeland Security* | 11/02/2020 | No search functionality. Filtered by “reports” and “journal articles” and date range 2002-2018. | 16 |
| *Department of Homeland Security* | 20/02/2020 | No search functionality in publications library. Filtered by “law enforcement partnerships” and hand-searched results for publications mentioning police and/or a response to terrorism/radicalisation/extremism within 2002-2018 date range. | 40 |
| *Public Safety Canada* | 19/02/2020 and 12/03/2020 | - Filtered by “counter-terrorism” in the Publications & Reports section. - Conducted two searches in the Public Safety Canada library with the same search terms (police OR policing OR “law enforcement” OR “law-enforcement”). Search 1 was in the title field and search 2 was in the subject field. Limiters applied: unchecked Commissioner’s Directions and DVDs under Sources, date range 2002-2018. - Conducted two searches in the Police Catalogue with the same search terms (terror* OR extrem* OR radicali* OR counter* OR de-radicali*). Search 3 was in the title field and search 4 was in the subject field. Limiters applied: unchecked Commissioner’s Directions and DVDs under Sources, date range 2002-2018. | 1,382 |
| *National Consortium for the Study of Terrorism and Responses to Terrorism (START)* | 11/02/2020 | No search functionality. Filtered by topic area “policing terrorism” and date range 2002-2018. | 85 |
| *Terrorism Research Centre* | 17/02/2020 | No search functionality. Hand-searched the publications section of the website for publications mentioning police and/or a response to terrorism/radicalisation/extremism within date range 2002-2018. | 2 |
| *Global Centre on Cooperative Security* | 11/02/2020 | Could not narrow down search via search filters (e.g., document type, country, author), and no text search functionality. Hand-searched the publications section of the website for publications mentioning police and/or a response to terrorism/radicalisation/extremism within date range 2002-2018. | 37 |
| *Hedayah* | 11/02/2020 | No search functionality on reports and publications page. Hand-searched the publications section of the website for publications mentioning police and/or a response to terrorism/radicalisation/extremism within date range 2002-2018. | 4 |
| *RAND Corporation* | 20/02/2020 | No search functionality. Filtered by topic area “terrorism” and category “research”. Hand-searched results for publications mentioning police and/or a response to terrorism/radicalisation/extremism within date range 2002-2018. | 30 |
| *Radicalisation Awareness Network (RAN)* | 17/02/2020 | No search functionality. Hand-searched the publications section of the website for publications mentioning police and/or a response to terrorism/radicalisation/extremism within date range 2002-2018. | 24 |
| *RadicalisationResearch* | 11/02/2020 | No search functionality. Examinations of this website revealed that the research indexed was predominantly journal articles from journals already captured by the systematic search. | 0 |
| *Royal United Services Institute (RUSI)* | 19/02/2020 | Not search functionality Filtered by topic area “terrorism” and date range 2002-2018. | 1 |
| *Impact Europe* | 19/02/2020 | No search functionality. Filtered by “internal publications” and “external publications” and date range 2002-2018. | 5 |
| *National Criminal Justice Reference Service* | 12/03/2020 | Due to limited search and export functionality, specific indexing terms provided by NJCRS that were related to terrorism were selected and all results displayed were manually added to and EndNote library. | 100 |

Table A2: Journal hand searches, trial registries, and expert consultation

| **Search component and source** | **Search date** | **Search strategy** | **N results** |
| --- | --- | --- | --- |
| Journal hand search:   - Critical Studies on Terrorism - International Journal of Conflict and Violence - Policing—An international Journal of Police Strategies and Management - Policing and Society - Sciences of Terrorism and Political Aggression - Studies in Conflict & Terrorism - Terrorism and Political Violence | 05/03/2020 | Using Web of Science platform, refined to the specific journal titles, searched the TOPIC field (captures title, abstract and subject fields) using the following search string: (police* OR policing OR “law enforcement”). Search was limited to the 12-months prior to 31^st^ December 2018. | 13 |
| Journal hand search:   - Dynamics of Asymmetric Conflict - Journal of Policing, Intelligence and Counter Terrorism | 05/03/2020 | Using Taylor and Francis platform, refined to the specific journal titles, searched on the title and keyword fields using the following search string: (police* OR policing OR “law enforcement”). Search was limited to the 12-months prior to 31^st^ December 2018. | 3 |
| Journal hand search:   - Journal for Deradicalization - Perspectives on Terrorism | 05/03/2020 | Using Directory of Open Access Journals platform, refined to the specific journal titles, searched on the title and keyword fields using the following search string: (police* OR policing OR “law enforcement”). Search was limited to the 12-months prior to 31^st^ December 2018. | 1 |
| Trial registries:   - World Health Organisation (WHO) International Trial Registry - Trial registries indexed on the Office for Human Research Protections website <https://www.hhs.gov/ohrp/international/clinical-trial-registries/index.html> | 05/03/2020 | WHO trial registry captured by the GPD systematic search. Five trial registries not captured by the WHO registry, but listed in on the Office for Human Research Protections website were individually searched using the same policing search terms used for the GPD systematic search (Health Canada Clinical Trial Database; Swiss National Clinical Trials Portal; Philippine Health Research Registry; South African National Clinical Trials Register; Tanzania Clinical Trial Registry) | 0 |
| Consultation with experts (*n* = 44)   - Campbell Crime and Justice Coordinating Group Steering Committee - Authors of Department of Homeland Security funded reviews (Sarah Carthy, Michael Wolfowicz) - Members of the Department of Homeland Security and Public Safety Canada Advisory Committee for suite of funded Campbell Collaboration reviews on countering violent extremism - International colleagues of Professors Mazerolle and Cherney who conduct research in the area of countering violent extremism and/or community-oriented policing approaches | 10/03/2020 | A truncated copy of the protocol, PRISMA flowchart, and a list of excluded topic relevant studies were emailed by Professors Mazerolle and Cherney | 0 |

Se

## Appendix C: GPD Systematic Search Strategy

### Search Terms

To ensure optimum sensitivity and specificity, the GPD search strategy utilises a combination of free-text and controlled vocabulary search terms. Because controlled vocabularies and search capabilities vary across databases, the exact combination of search terms and field codes are adapted to each database. Final search syntax for each location will be reported in the final review.

The free-text search terms for the GPD are provided in Table 1 and are grouped by substantive (i.e., some form of policing) and evaluation terminology. Although the search strategy may vary slightly across search locations, it follows a number of general rules:

- Search terms are combined into search strings using Boolean operators “AND” and “OR”. Specifically, terms within each category are combined with “OR”, and categories will be combined with “AND”. For example: (police OR policing OR “law#enforcement”) AND (analy* OR ANCOVA OR ANOVA OR …).
- Compound terms (e.g., law enforcement) are considered single terms in search strings by using quotation marks (i.e., “law*enforcement”) to ensure that the database searches for the entire term rather than separate words.
- Wild cards and truncation codes are used for search terms with multiple iterations from a stem word (e.g., evaluation, evaluate) or spelling variations (e.g., evaluat* or randomi#e).
- If a database has a controlled vocabulary term that is equivalent to “POLICE”, the term is combined in a search string that includes both the policing and evaluation free-text search terms. This approach ensures that the search retrieves documents that do not use policing terms in the title/abstract but have been indexed as being related to policing in the database. An example of this approach is the following search string: (((SU: “POLICE”) OR (TI,AB,KW: police OR policing OR “law*enforcement”)) AND (TI,AB,KW: intervention* OR evaluat* OR compar* OR …)).
- For search locations with limited search functionality, a broad search that uses only the policing free-text terms is implemented.
- Multidisciplinary database searches are limited to relevant disciplines (e.g., include social sciences but exclude physical sciences).
- Search results are refined to exclude specific types of documents that are not suitable for systematic reviews (e.g., newspapers, front/back matter, book reviews).

#### Table 1. Free-text search terms for the GPD systematic search

| **Policing Search Terms** | **Evaluation Search Terms** | | | |
| --- | --- | --- | --- | --- |
| police  policing  “law*enforcement”  constab*  detective*  sheriff* | analy*  ANCOVA  ANOVA  “ABAB design”  “AB design”  baseline  causa*  “chi#square”  coefficient*  “comparison condition*”  “comparison group*”  “control condition*”  “control group*”  correlat*  covariat*  “cross#section*” | data  effect*  efficacy  eval*  experiment*  hypothes*  impact*  intervent*  interview*  longitudinal  MANCOVA  MANOVA  “matched group”  measure*  “meta-analy*”  “odds#ratio* | outcome*  paramet*  “post-test”  posttest  “post test”  predict*  “pre-test”  pretest  program*  “propensity score*”  quantitative  “quasi#experiment*”  questionnaire*  random*  RCT  regress* | result*  “risk#ratio*”  sampl*  “standard deviation*”  statistic*  studies  study  survey*  “systematic review*”  “t#test*”  “time#series”  treatment*  variable*  variance |

### Search Locations

To reduce publication and discipline bias, the GPD search strategy adopts an international scope and involves searching for literature across a number of disciplines (e.g., criminology, law, political science, public health, sociology, social science and social work). The search captures a comprehensive range of published (i.e., journal articles, book chapters, books) and unpublished literature (e.g., working papers, governmental reports, technical reports, conference proceedings, dissertations) by implementing a search strategy across bibliographic/academic, grey literature, and dissertation databases or repositories.

It is noted that there is substantial overlap of the content coverage between many of the databases. Therefore, the *Optimal Searching of Indexing Databases* (OSID) computer program (Neville & Higginson, 2014) has been used to analyse the content crossover for all databases that have accessible content coverage lists. OSID analyses the content coverage and creates a search location solution that provides the most comprehensive coverage via the least number of databases. Another advantage of using OSID when designing a search strategy is the reduction in the number of duplicates that would need to be removed prior to the screening phase. Databases with >10 unique titles are searched in full, whereas databases with ≤10 unique titles were searched only the unique titles and any non-serial content (e.g., reports, conference proceedings). Where a modified search of a database would be more labour-intensive than a full search and export results, a full search of the database is conducted. The final search locations and solutions are reported in Table 2.

#### Table 2. GPD search locations and protocol (January 1^st^ 1950 – December 2018)

| **INDEXED & ACADEMIC DATABASES** |  | **CONTENT COVERAGE FED INTO OSID?** | **FULL OR MODIFIED SEARCH?** | **SEARCH MODIFICATIONS** |
| --- | --- | --- | --- | --- |
| **ProQuest** | Criminal Justice | Yes | Full | None. |
|  | Dissertation and Theses Database Global | Not Available | Modified | Social Sciences subset. |
|  | Political Science | Yes | Full | None. |
|  | Periodical Archive Online | Yes | Full | None. |
|  | Research Library | Yes | Modified | Social Sciences subset. |
|  | Social Science Journals | Yes | Full | None. |
|  | Sociology | Yes | Modified | Search 2 unique journal titles and non-serial content only. |
|  | Applied Social Sciences Index and Abstracts | Yes | Full | None. |
|  | International Bibliography of the Social Sciences | Yes | Full | None. |
|  | Public Affairs Information Service | Yes | Full | None. |
|  | Social Services Abstracts | Yes | Modified | Search 5 unique journal titles and non-serial content only. |
|  | Sociological Abstracts | Yes | Full | None. |
|  | Worldwide Political Sciences Abstracts | Yes | Modified | Search 9 unique journal titles and non-serial content only. |
| **EBSCO** | Academic Search Premier | Yes | Full | None. |
|  | Criminal Justice Abstracts | Yes | Full | None. |
|  | EconLit | Yes | Full | None. |
|  | MEDLINE with Full-Text | Yes | Full | None. |
|  | Social Sciences Full-Text | Yes | Full | None. |
| **OVID** | International Political Science Abstracts | Not Available | Full | None. |
|  | PsycARTICLES | Yes | Modified | Search 4 unique journal titles only. |
|  | PsycEXTRA | Not Available | Full | None. |
|  | PsycINFO | Yes | Full | None. |
|  | Social Work Abstracts | Not Available | Full | None. |
| **Web of Science** | Current Contents Connect – Social and Behavioural Sciences Edition | Yes | Modified | Search 1 unique journal title and non-serial content only. |
|  | Book Citation Index (Social Sciences and Humanities) | Not Available | Full | None. |
|  | Conference Proceedings Citation Index (Social Sciences and Humanities) | Not Available | Full | None. |
|  | Social Science Citation Index | Yes | Full | None. |
| **Informit** | Australian Attorney General Information Service | Yes | Full | None. |
|  | Australian Criminology Database (CINCH) | Yes | Full | None. |
|  | Australian Federal Police Database | Yes | Full | None. |
|  | Australian Public Affairs Full-Text | Yes | Full | None. |
|  | DRUG | Yes | Full | None. |
|  | Health & Society Database | Yes | Modified | Search unique journal titles and non-serial content only. |
|  | Humanities and Social Sciences Collection | Yes | Full | None. |
| **Gale-Cengage** | Expanded Academic ASAP | Yes | Full | None. |
| **STANDALONE & OPEN ACCESS DATABASES** | Cambridge Journals Online | Yes | Modified | Search 4 unique journal titles in Law and Political Science collections and full search of Social Studies collection. |
|  | Directory of Open Access Journals | Yes | Full | None. |
|  | HeinOnline | Yes | Modified | Law Journals Online collection only. |
|  | JSTOR | Yes | Modified | Search unique titles across the Law, Political Science, Public Health, Public Policy, Social Work and Sociology collections only. The Criminal Justice collection had no unique content and so will be excluded from the search. Only 10% of content in this database have abstracts and a full-text search returns >250,000 results because of inability to construct complex search strings. Therefore, a modified search of the unique titles across these collections will be more pragmatic than a full search of the database. |
|  | Oxford Scholarship Online | Yes | Full | None. |
|  | Sage Journals Online and Archive (Sage Premier) | Yes | Modified | Search 5 unique journal titles and non-serial content only. |
|  | ScienceDirect | Yes | Full | None. |
|  | SCOPUS | Yes | Full | None. |
|  | SpringerLink | Yes | Full | Although this database has low uniqueness when combined with the full set of databases, a full search using only the policing search terms will be more pragmatic than a modified search on unique titles because of the restricted search functionality of this database. |
|  | Taylor & Francis Online | Yes | Modified | Although this database has low uniqueness when combined with the full set of databases, a full search using only the policing search terms will be more pragmatic than a modified search on unique titles because of the restricted search functionality of this database. |
|  | Wiley Online Library | Yes | Full | None. |
|  | California Commission on Peace Officer Standards & Training Library | No | Full | None. |
|  | Cochrane Library | No | Full | None. |
|  | CrimeSolutions.gov | No | Full | None. |
|  | Database of Abstracts of Reviews of Effectiveness (DARE) | No | Full | None. |
|  | FBI – The Fault (Reports and Publications) | No | Full | None. |
|  | Evidence-Based Policing Matrix | No | Full | None. |
|  | International Initiative for Impact Evaluation Database (3ie) | No | Full | None. |
|  | National Criminal Justice Reference Service | No | Full | None. |
|  | Safety Lit Database | No | Full | None. |
|  | Australian Institute of Criminology | No | Full | None. |
|  | Bureau of Police Research and Development (India) | No | Full | None. |
|  | Canadian Police Research Catalogue | No | Full | None. |
|  | Centre for Problem-Oriented Policing | No | Full | None. |
|  | College of Policing (including POLKA and Crime Reduction Toolkit) | No | Full | None. |
|  | European Police College (CEPOL) | No | Full | None. |
|  | Evidence for Policy and Practice Information and Coordinating Centre | No | Full | None. |
|  | National Research Institute of Police Science (Japanese) | No | Full | None. |
|  | Office of Community Oriented Policing Services | No | Full | None. |
|  | Police Executive Research Forum (US) | No | Full | None. |
|  | Police Foundation (US) | No | Full | None. |
|  | Tasmania Institute of Law Enforcement Studies (Australia) | No | Full | None. |
|  | Policing Online Information System (POLIS, Europe) | No | Full | None. |
|  | Scottish Institute for Policing Research | No | Full | None. |
|  | Centre of Excellence in Policing and Security (Australian, now archived) | No | Full | None. |

## Appendix B: GPD Systematic Compilation Strategy

### Inclusion Criteria

Each record captured by the GPD systematic search must satisfy all inclusion criteria to be included in the GPD: timeframe, intervention and research design. There are no restrictions applied to the types of outcomes, participants, settings or languages considered eligible for inclusion in the GPD.

#### Types of interventions

Each document must contain an impact evaluation of a policing intervention. Policing interventions are defined as some kind of a strategy, program, technique, approach, activity, campaign, training, directive, or funding/organisational change that involves police in some way (other agencies or organisations can be involved). Police involvement is broadly defined as:

- Police initiation, development or leadership
- Police are recipients of the intervention or the intervention is related, focused or targeted to police practices
- Delivery or implementation of the intervention by police

#### Types of study designs

The GPD includes quantitative impact evaluations of policing interventions that utilise randomised experimental (e.g., RCTs) or quasi-experimental evaluation designs with a valid comparison group that does not receive the intervention. The GPD includes designs where the comparison group receives ‘business-as-usual’ policing, no intervention or an alternative intervention (treatment-treatment designs).

The specific list of research designs included in the GPD are as follows:

- Systematic reviews with or without meta-analyses
- Cross-over designs
- Cost-benefit analyses
- Regression discontinuity designs
- Designs using multivariate controls (e.g., multiple regression)
- Matched control group designs with or without pre-intervention baseline measures (propensity or statistically matched)
- Unmatched control group designs with pre-post intervention measures which allow for difference-in-difference analysis
- Unmatched control group designs without pre-intervention measures where the control group has face validity
- Short interrupted time-series designs with control group (less than 25 pre- and 25 post-intervention observations)
- Long interrupted time-series designs with or without a control group (≥25 pre- and post-intervention observations)
- Raw unadjusted correlational designs where the variation in the level of the intervention is compared to the variation in the level of the outcome

The GPD excludes single group designs with pre- and post-intervention measures as these designs are highly subject to bias and threats to internal validity.

### Systematic Screening

To establish eligibility, records captured by the GPD search are progress through a series of systematic stages which are summarised in Figure 1, with additional detail provided in the following subsections.

All research staff working on the GPD undergo standardised training before beginning work within any of the stages detailed below. Staff then complete short training simulations to enable an assessment of their understanding of the GPD protocols and highlight any areas for additional training. In addition, random samples of each staff’s work are regularly cross-checked to ensure adherence to protocols. Disagreements about screening decisions between staff are mediated by either the project manager or GPD chief investigators.

#### Title and abstract screening

After removing duplicates, the title and abstract of records captured by the GPD systematic search is screened by trained research staff to identify potentially eligible research that satisfies the following criteria:

- Document is dated between 1950 – present
- Document is unique (i.e., not a duplicate)
- Document is about police or policing
- Document is an eligible document type (e.g., not a book review)

Records are excluded if the answer to any one of the criteria is unambiguously ‘No’, and will be classified as potentially eligible otherwise. Records classified as eligible at the title and abstract screening stage progress to full-text document retrieval and screening stages.

#### Full-text eligibility screening

Wherever possible, a full-text electronic version of an eligible record is imported into *SysReview* (review management software; Higginson & Neville, 2015). For records without an electronic version, a hardcopy of the record is located to enable full-text eligibility screening. The full-text of each document is screened to identify studies that satisfy the following criteria:

- Document is dated between 1950 – present
- Document is unique
- Document reports a quantitative statistical comparison
- Document reports on policing evaluation
- Document reports in a quantitative impact evaluation of a policing intervention
- Evaluation uses an eligible research design

| **SYSTEMATIC SEARCH OF PUBLISHED & UNPUBLISHED LITERATURE** |
| --- |
|  |
| **EXPORT SEARCH RESULTS**   - Bibliographic data and abstracts exported into EndNote - Data cleaned and duplicate records removed |
|  |
| **IMPORT SEARCH RESULTS INTO *SYSREVIEW*** |
|  |
| **SCREEN TITLES AND ABSTRACTS FOR ELIGIBILITY**   1. Not a duplicate document? 2. Between 1950 – present? 3. About police or policing? 4. Eligible document type?   ***If not clearly excluded on any criteria…*** |
|  |
| **DOCUMENT RETRIEVAL**   - Retrieve electronic and hard copies of all eligible documents - Attach electronic versions to records in *SysReview* |
|  |
| **SCREEN FULL-TEXT OF DOCUMENTS**  **FOR FINAL ELIGIBILITY**   1. Not a duplicate document? 2. Between 1950 – present? 3. Quantitative statistical comparison? 4. Policing intervention? 5. Quantitative impact evaluation? 6. Eligible research design?   ***If ‘Yes’ to all…*** |
|  |
| **CATEGORISE ELIGIBLE DOCUMENTS**   1. Research design 2. Intervention location 3. Publication date 4. Problem targeted 5. Evaluation outcome measure(s) 6. Type of policing intervention |
|  |
| **GLOBAL POLICING DATABASE (GPD)**  Web-based  Searchable  Updated biennially |

*Figure 1.* GPD systematic compilation process

## Appendix D: Full-Text Coding Form^[[1]](#footnote-1)^

**General Study Details**

1. Study ID [textbox]

2. Report ID [textbox]*

3. What type of document is this study? [*dropdown menu*]

- 1. Peer-reviewed journal article
  2. Book chapter
  3. Dissertation
  4. Conference presentation
  5. Government report, technical report, or working paper
  6. Other (specify in textbox)

4. In what country was the intervention implemented? [*textbox*]

5. If the evaluation and/or intervention was funded, record the funding source. [*textbox*]

*SysReview allows for multiple reports of a single study to be included in the one full-text coding record form. Each report is nested within the overall study record and the Report ID will consist of the Study ID followed by a unique alphabetical code (e.g., 1234_a, 1234_b…).

**Participants**

1. Who are the participants? [*checkboxes*]
   1. Citizens
   2. Practitioners (specify in textbox)
   3. Micro places (specify in textbox)
   4. Macro places (specify in textbox)
   5. Other (specify in textbox)
2. How were participants recruited? [*textbox*]
3. What were the eligibility criteria for inclusion in the study? [*textbox*]
4. Describe sample attrition. [*textboxes*]

| **Number of Participants** | **Treatment** | **Comparison** | **Total** |
| --- | --- | --- | --- |
| Referred to study |  |  |  |
| Consented |  |  |  |
| Assigned |  |  |  |
| Began intervention |  |  |  |
| Completed intervention |  |  |  |
| Completed follow-up 1 |  |  |  |
| Completed follow-up 2 (if applicable) |  |  |  |

1. Describe the characteristics of the sample. [*textboxes*]

| **Number of Participants** | **Treatment** | **Comparison** | **Total** |
| --- | --- | --- | --- |
| Age (M, SD, range) |  |  |  |
| Gender (% female) |  |  |  |
| Ethnicity (proportions) |  |  |  |
| Socioeconomic status (proportions) |  |  |  |

1. Record any other pertinent sample information for both the treatment and comparison groups. [*textbox*].

**General Methodological Details and Nature of Comparisons**

1. What is the nature of the comparisons for this study? [*dropdown menu*]
   1. Single intervention contrasted with single comparison condition
   2. Multiple interventions against a single comparison condition
   3. Within one group over time
   4. Other (specify in textbox)
2. General research design classification [*dropdown menu*]
   1. Randomised controlled trial
   2. Quasi-randomised controlled trial
   3. Non-randomised controlled trial (e.g., interrupted time-series, matched control group design)
   4. Other (specify in textbox)
3. What type of comparison condition was used? [*dropdown menu*]
   1. No treatment
   2. Treatment-as-usual (specify in textbox)
   3. Alternative treatment (specify in textbox)
   4. Waitlist control
   5. Other (specify in textbox)
4. How were treatment and comparison groups formed? [*dropdown menu*]
   1. Random allocation
   2. Matching (specify matching method and matching variables in textbox)
   3. On basis of score on a specific measure (e.g., diagnosis, specify in textbox)
   4. Self-selection
   5. Other (specify in textbox)
   6. Unclear
5. What was the unit of allocation? [*dropdown menu*]
6. Participant
7. Dyads
8. Family
9. Service site
10. Other (specify in textbox)
11. Unclear
12. If participants were randomly allocated to conditions, how was this implemented? [*dropdown menu*]
13. Simple
14. Yoked pairs
15. Cluster (specify cluster in textbox)
16. Block/stratified (specify variables in textbox)
17. Matched pairs (specify matching variables in textbox)
18. Other (specify in textbox)
19. Unclear
20. Not applicable
21. Who executed the randomisation? [*dropdown menu*]
22. Researchers
23. Practitioners
24. Other (specify in textbox)
25. Unclear
26. If applicable, was randomisation equivalent across intervention sites? [*dropdown menu*]
27. Yes
28. No
29. Unclear
30. No applicable
31. Was group equivalence assessed? [*dropdown menu*]
32. Yes (specify how this was done in textbox)
33. No
34. Unclear
35. Not applicable
36. Were the treatment and comparison groups equivalent at baseline? [*dropdown menu*]
37. Yes
38. No (specify differences)
39. Unsure
40. Not applicable
41. Are there any differences between participants who completed versus did not complete the treatment? [*dropdown menu*]
42. Yes (specify differences)
43. No
44. Unsure
45. Not applicable
46. What was the unit of analysis? [*dropdown menu*]
47. Participant
48. Dyads
49. Family
50. Service site
51. Other (specify in textbox)
52. Unclear

**Intervention Details**

- 1. What is the name of the intervention(s), as reported by study authors? [*textbox*]
  2. What settings were used during the intervention(s) (e.g., community, institutions etc)? [*textbox*]
  3. When was the intervention conducted (e.g., year)? [*textbox*]
  4. Describe the intervention provided to participants, ensuring you record the specific components or materials implemented and the mode of implementation. [*textbox*]
  5. Describe the duration of the entire intervention. If reported, describe the minimum, maximum, mean and standard deviation for intervention duration. [*textbox*]
  6. Describe the intensity of the intervention (e.g., frequency of contacts and length of contacts). If reported, describe the minimum, maximum, mean and standard deviation for intervention intensity. [*textbox*]
  7. Who implemented the intervention? [*textbox*]
  8. Was there more than one intervention site? [*dropdown menu*]
  9. Yes (specify number of sites in textbox)
  10. No
  11. Unclear
  12. Was treatment integrity monitored? [*dropdown menu*]
  13. Yes (specify in textbox)
  14. No
  15. Unclear
  16. Were there any issues with fidelity? [*dropdown menu*]

1. Yes (specify in textbox)
2. No
3. Unclear
   1. Did the authors report cost-benefit data? [*dropdown menu*]
4. Yes (specify in textbox)
5. No
6. Unclear

**Outcome(s) Measurement***

*To be completed for each eligible outcome within a study (or group of reports for a study). To add another outcome, click the ‘Add another outcome’ button located at the bottom of the screen.

- 1. What is the outcome being measured? [*textbox*]
  2. How was the outcome data gathered? [*dropdown menu*]
  3. Self-report
  4. Observation
  5. Official source
  6. Interview
  7. Other (specify in textbox)
  8. What are the psychometric properties of the measurement tool (e.g., reliability, validity, diagnostic thresholds, what higher /lower values mean)? [*textbox*]
  9. Who was the respondent/participant? [*textbox*]
  10. At what time-point(s) was the outcome measured? [*textbox*]
  11. Were data collected in the same manner for the treatment and comparison conditions? [*dropdown menu*]
  12. Yes
  13. No (specify in textbox)
  14. Unclear
  15. Which condition does the raw difference/effect favour (ignore statistical significance)? [*dropdown menu*]
  16. Experimental condition
  17. Comparison condition
  18. Neither condition (no difference)
  19. Unclear
  20. In which direction did the outcome change? [*dropdown menu*]
  21. Positive
  22. Negative
  23. Mixed (specify in textbox)
  24. Unclear
  25. Were there statistically significant differences for this outcome? [*dropdown menu*]
  26. Yes
  27. No
  28. Not tested
  29. Unclear
  30. What were the study author(s)’ conclusions about this outcome? [*textbox*]

**Effect Size Data***

*To be completed for each eligible outcome within a study (or group of reports for a study). To add another outcome, click the ‘Add another outcome’ button located at the bottom of the screen.

- 1. On what page number is the effect size data reported? [*textbox*]
  2. What type of effect size is being coded? [*dropdown menu*]
  3. Post-intervention only (first point of measurement after intervention)
  4. Baseline and post-intervention or pre-test measure prior to intervention)
  5. Follow-up (subsequent point of measurement after first post-test)
  6. What is the timeframe captured for the measure?
  7. Minimum [*textbox*]
  8. Maximum [*textbox*]
  9. Mean [*textbox*]
  10. Same for all participants (i.e., fixed) [*textbox*]
  11. How was the effect size obtained for this outcome? [dropdown menu]
  12. Reported in document → Go to Question 5
  13. Calculated by user → Go to Question 6
  14. Identify the type of effect size reported for this outcome and enter the required data for that effect size in the text boxes provided. [*textboxes*]

**
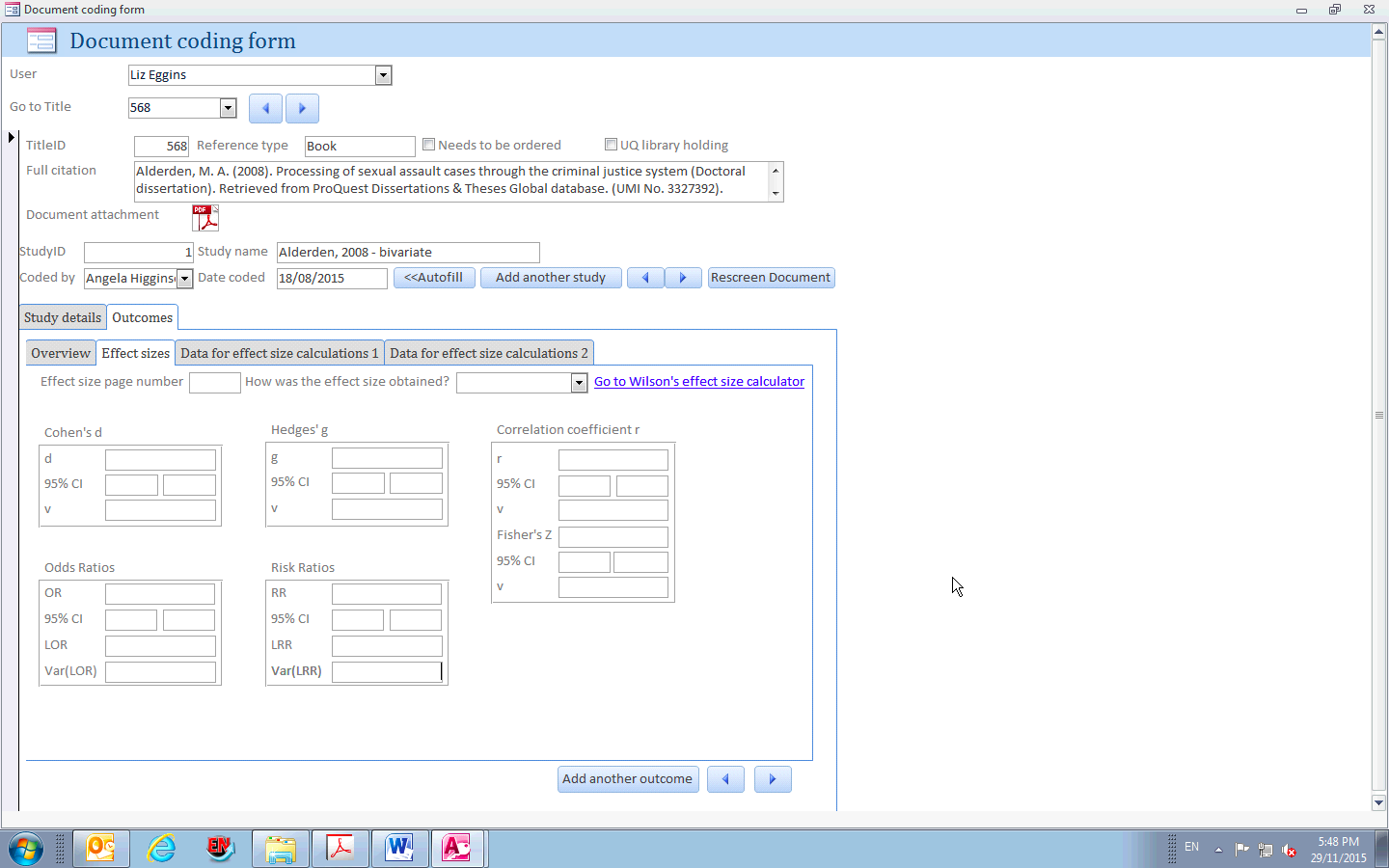
**

- 1. Enter the appropriate data in the relevant ‘Data for effect size calculations’ tabs (see below). The data entered will depend on what is reported in the document. If none of the circumstances in the tabs reflect the data in the document, follow the link to David Wilson’s online effect size calculator to calculate an effect size. You can enter the data in the ‘Data for effect size calculations 2’ tab in the ‘Other information’ textbox. [*textboxes*]


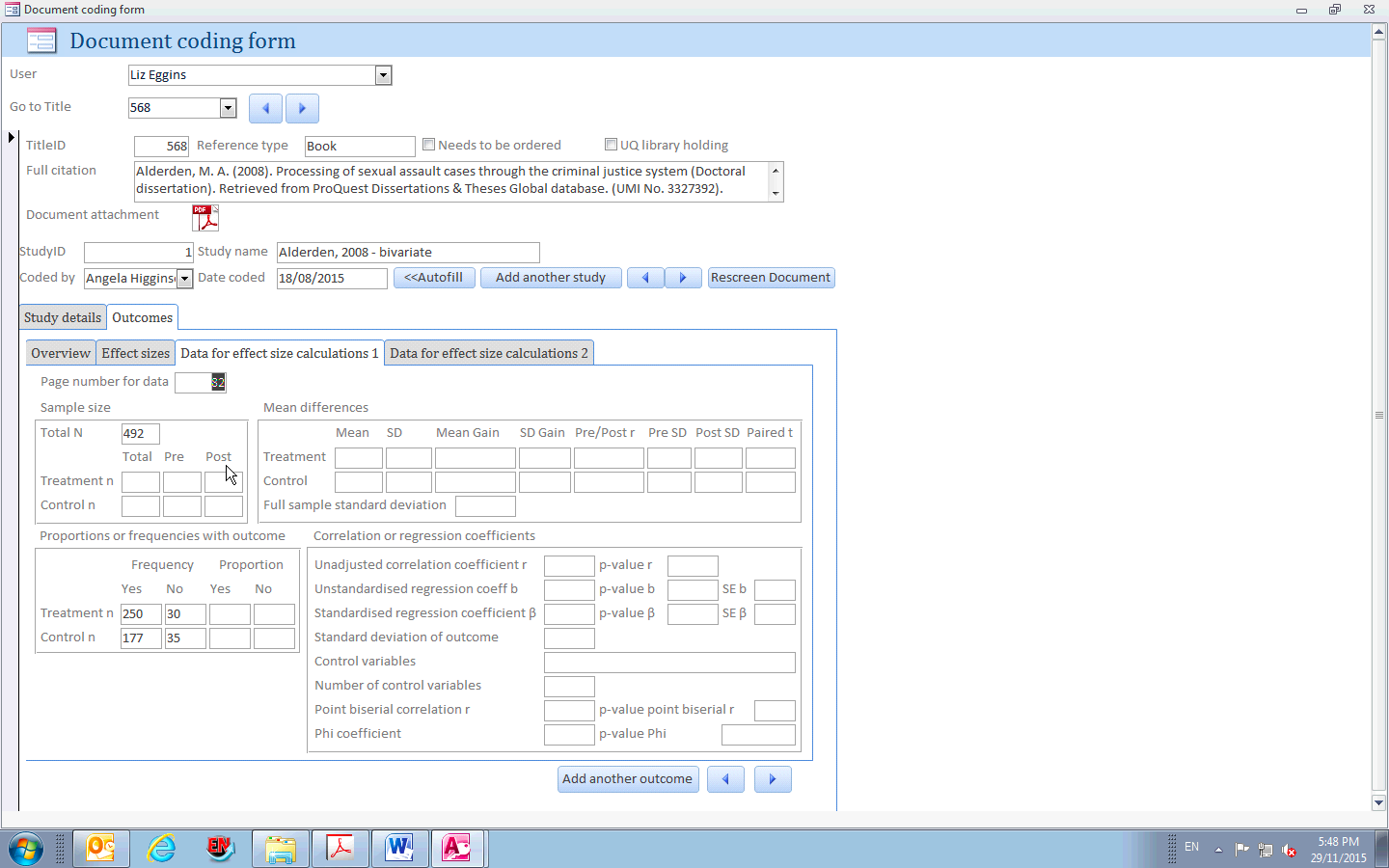


**
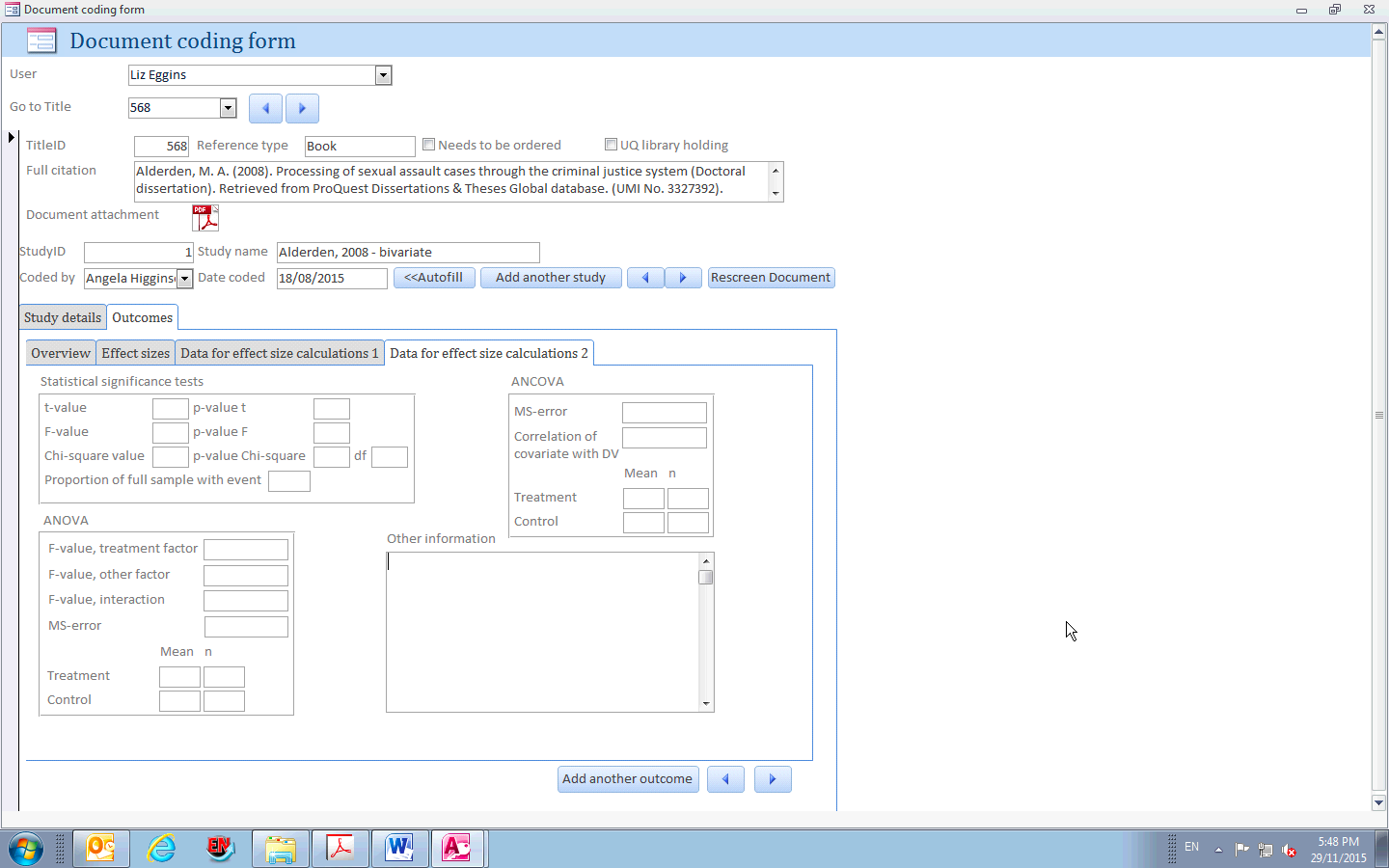
**

1. This form has been informed by published coding forms (e.g., Littel et al., 2008; Mazerolle, Higginson, & Eggins, in press; Mitchell, Wilson, Eggers, & MacKenzie, 2012). [↑](#footnote-ref-1)
